# Flight of the dragonflies and damselflies

**DOI:** 10.1098/rstb.2015.0389

**Published:** 2016-09-26

**Authors:** Richard J. Bomphrey, Toshiyuki Nakata, Per Henningsson, Huai-Ti Lin

**Affiliations:** 1 Structure and Motion Laboratory, Department of Comparative Biomedical Sciences, Royal Veterinary College, North Mymms, Hatfield AL9 7TA, UK; 2 Graduate School of Engineering, Chiba University, 1-33, Yayoi-cho, Inage-ku, Chiba-shi, Chiba 263-8522, Japan; 3 Department of Biology, Lund University, Ecology Building, 223 62 Lund, Sweden; 4 Howard Hughes Medical Institute, Janelia Research Campus, 19700 Helix Drive, Ashburn, VA 20147, USA

**Keywords:** Odonata, flight, biomechanics, aerodynamics, prey capture, performance

## Abstract

This work is a synthesis of our current understanding of the mechanics, aerodynamics and visually mediated control of dragonfly and damselfly flight, with the addition of new experimental and computational data in several key areas. These are: the diversity of dragonfly wing morphologies, the aerodynamics of gliding flight, force generation in flapping flight, aerodynamic efficiency, comparative flight performance and pursuit strategies during predatory and territorial flights. New data are set in context by brief reviews covering anatomy at several scales, insect aerodynamics, neuromechanics and behaviour. We achieve a new perspective by means of a diverse range of techniques, including laser-line mapping of wing topographies, computational fluid dynamics simulations of finely detailed wing geometries, quantitative imaging using particle image velocimetry of on-wing and wake flow patterns, classical aerodynamic theory, photography in the field, infrared motion capture and multi-camera optical tracking of free flight trajectories in laboratory environments. Our comprehensive approach enables a novel synthesis of datasets and subfields that integrates many aspects of flight from the neurobiology of the compound eye, through the aeromechanical interface with the surrounding fluid, to flight performance under cruising and higher-energy behavioural modes.

This article is part of the themed issue ‘Moving in a moving medium: new perspectives on flight’.

## 1. Introduction

The early diversification of insects is still under discussion but it is clear that the Odonata, including modern dragonflies (Anisoptera) and damselflies (Zygoptera), are derived from Palaeopterans that also included the earliest fossil fliers from the Late Carboniferous. One of the Meganisoptera grew to a wingspan of approximately 70 cm and resembled a modern dragonfly in many respects, including having a broader hindwing than forewing, a broad thorax thought to contain powerful flight muscles, large mandibles and spiny legs that make Odonata such effective predators [1,2]. Extant Odonata display impressive diversity, not least in size. The East Asian dragonfly *Nannophya pygmaea* has a wingspan of just 20 mm, whereas the forest giant damselfly, *Megaloprepus caerulatus* with a wingspan an order of magnitude higher, feeds by plucking orb weaving spiders from their webs in Central and South America. Early evolutionary history also means that Odonata can be found on every continent except Antarctica. In total, 7500 species of Odonata are known with 60 new African species described in 2015 [3]. The evolutionary success of this group despite relatively minor changes in anatomy in more than 300 million years makes their mechanical, physiological and behavioural flight strategies worthy of investigation in the context of both biology and engineering.

Adulthood is a relatively short portion of the Odonatan life cycle in comparison with their longer aquatic juvenile stage but it is plainly an important one. As adults, survivorship may be dependent on effective commuting, flight performance during hawking (continuous prey seeking on the wing) or darting foraging, prey recognition, targeting, interception and capture, predator evasion and, in some species, fuel economy and navigation during migration flights. Fecundity relies on successful conspecific recognition, courtship, copulation, successful oviposition and in many cases, the guarding of mates either by close patrols or tandem flights. Migration is also a big challenge for some dragonflies to exploit seasonal resources. Common green darners (*Anax junius*) have been observed [4] and tagged with radio transmitters [5] in the Midwest and Eastern United States. Their migratory guidance appears to be correlated with linear features in the terrain below, therefore requiring visual cues for navigation. On the other hand, globe skimmers' (*Pantala flavescens*) epic migration across the Indian Ocean is driven by strong, high-altitude winds that are associated with the intertropical convergence zone [6,7]. During these flights, there will be little opportunity to forage, so flight should be tuned for the minimal cost of transport, with high-energy aerobatic manoeuvres limited to evading hawks and other predators that follow convergent migration routes [6]. Such epic journeys are particularly impressive when bearing in mind these intercontinental dragonflies typically weigh on the order of 2 g.

## 2. Wing musculoskeletal architecture

The phylogenetic relationship between the Odonata, Ephemeroptera and the Neoptera remains controversial, and dragonflies have been crucial in efforts to determine the origin of the flight apparatus and wing folding mechanism that separate the Palaeoptera from the Neoptera. It remains a challenge to unambiguously determine the homologous structures amongst dragonflies and other Pterygota, particularly the complex muscle arrangement. Büsse and Hörnschemeyer investigated Libellulids, Aeschnids and Cordulegasterids, identifying 71 muscles in the thorax, seven of which had no homologous muscle in the Neopteran thorax [8]. Many of these muscles insert on the radial veins, giving active control over the angle of attack, camber, twist, amplitude and frequency of each of the four wings independently. Regional positional control of the wing is enhanced further by passively prescribed motions governed by the wing architecture, including vein curvature, vein cross sections that promote torsion but resist bending [9], flexible resilin vein junctions [10], the arculus trailing edge depressor [11], the nodus [12], the pterostigma inertial regulator of wing pitch [13] and Arnold flow in the veins [14] as a regulator of wing mass [15].

The wings are hierarchical structures [16] with functionally significant detail from the cellular level to the architectural level of the wing vein patterning. There is a rich adornment of spines and hairs that are sensitive not only to the flow direction and speed but can also influence the fluid dynamics directly as air passes over the wing, encouraging the transition from laminar to turbulent flow in the boundary layer. A cross section through the leading edge of *Aeshnid* dragonflies reveals a T shape, composed of three rows of serrations thought to act as another type of flow control device, called turbulators [17]. At the larger scale, the attractive grid of wing veins that support the membrane are likely to act (in a similar way to those in the hindwing of desert locusts [18]) as a rip-stop device, protecting the wing from damage during collisions by improving fracture toughness. The planform of both the fore- and hindwings has been shown, using phylogenetically controlled geometric morphometrics methods, to correlate with long-distance migration in the Anisoptera [19]. The planform will have an influence on the aerodynamic and inertial characteristics of the wings, but the nature of these interactions is yet to be resolved fully.

## 3. Gliding flight aerodynamics: corrugations and tandem wings

Dragonfly wings, in common with those of other insects, are not smooth surfaces but have distinct corrugations [17]. These corrugations define the stressed skin structure composed of girder-like veins and thin cuticle membrane. Such complex geometry has been a feature of insect wings since the Palaeozoic [11,12,20], providing sophisticated mechanical advantages for resisting longitudinal bending [20–23] while facilitating wing camber and torsion [24], and enabling predictable, beneficial buckling, both within the normal wing stroke cycle and in response to sudden loads [21]. The aerodynamic effect of corrugations has been investigated largely in just two dimensions, using physical [25–27] and computational models [28]. It has been found that the incident flow separates at the ridges, enveloping recirculating eddies that might play a role in reducing skin friction drag or modulating the lift coefficient (summarized in [29]). Three-dimensional models of insect wing corrugations have been limited to extrusions of chord profiles [25,26,30–32] that are often based on a very limited set of measurements from a single wing of dried specimens, overlooking the consequences of spanwise variation in corrugation pattern, curvature of the ridges and valleys within the plane of the wing membrane, spanwise twist, three-dimensional aerodynamic effects, individual variation and interspecies diversity.

**Figure 1.**
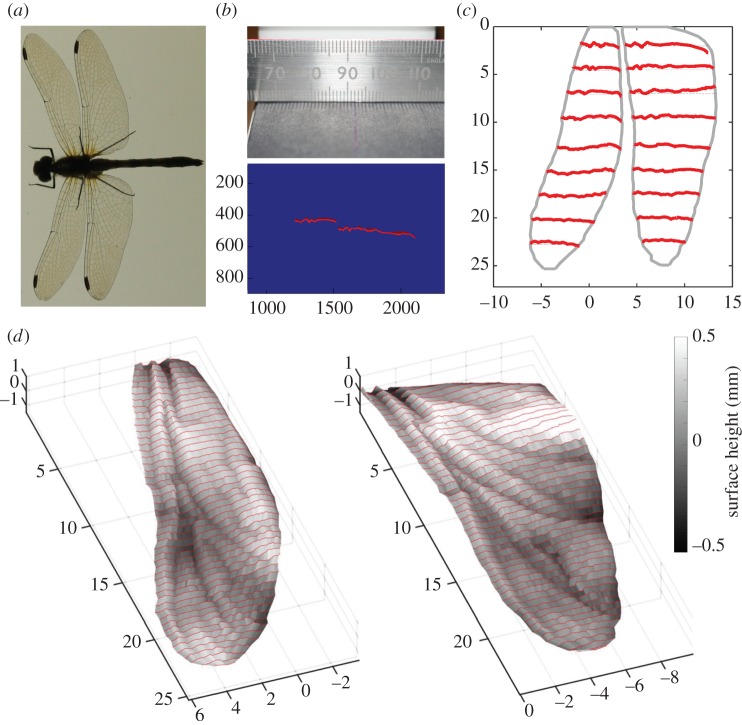
Determining the three-dimensional geometry of dragonfly wings. The common darter (*Sympetrum sanguineum*) is (*a*) photographed on a lightbox before being attached to a micromanipulator and traversed in millimetre intervals through a vertical laser light sheet parallel with the sagittal plane. The bright lines reflecting from the wings are photographed from an axis near perpendicular to the sagittal plane and (*b*) the pixel positions are converted to chordwise profiles by camera calibration and corrected for perspective. This yields many chord profiles at high resolution, some of which are shown in (*c*), that can be used to create surfaces (*d*) demonstrating the complex three-dimensional geometry of the wings and which are suitable for CFD analysis.

Here, we used a scanning laser projection method to reconstruct three-dimensional wing geometries by photographing cross sections illuminated by a laser line generator and traversing subjects through a calibrated plane in millimetre intervals using a micromanipulator. The images were thresholded to isolate the chord profile at each spanwise station; a schematic of the protocol is shown in figure 1. We provide detailed three-dimensional wing geometries of 52 Anisopteran individuals comprising 17 species (electronic supplementary material, figure S1 and data) but focus now on the ruddy darter (*Sympetrum sanguineum*), performing computational fluid dynamics (CFD) analyses of gliding flight using a versatile low Reynolds number aerodynamic simulator [33]. Corrugation pattern and amplitude vary greatly along the span and their contribution to aerodynamic performance was evaluated by comparing the full-fidelity model with artificial wing shapes.

**Figure 2.**
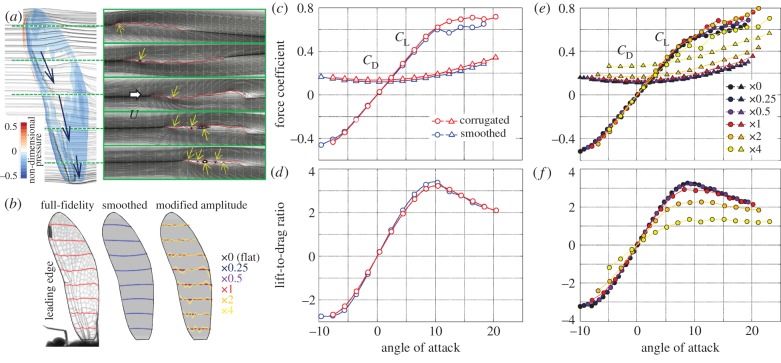
The effect of wing corrugation on the gliding aerodynamics of a dragonfly forewing at *Re* = 730 [33]. The simulations are performed with a local forewing grid (301×321×21) and a larger global grid (301×321×21; 15 times mean chord length to the outer boundary). The non-dimensional time step is set to be 0.01. (*a*) Three-dimensional and two-dimensional streamlines around the forewing of *Sympetrum sanguineum*. The vortices in the valley highlighted by yellow arrows help to form a smooth envelope. (*b*) Selected cross sections of the full-fidelity wing, smoothed wing and the wings with modified amplitude. (*c*) Coefficients of lift and drag and (*d*) the lift-to-drag ratio for the full-fidelity and smoothed wing models (angle of attack is defined relative to the zero-lift angle). The coefficients are obtained after convergence of the lift and drag (at least 10 convective time steps) to exclude transient effects. (*e,f*) Aerodynamic performance of the exaggerated and reduced corrugation models.

Two-dimensional streamlines at five spanwise positions are shown in figure 2*a*, supporting the notion that vortices form in the valleys with the streamlines defining a smoother envelope [31]. Interestingly, our three-dimensional method also reveals the development of tip-to-root spanwise flows within the core of vortices in the deep valleys close to the leading edge during gliding flight. As expected, the general pattern is for low pressures to occur in the valleys and higher pressures to occur on the forward facing surfaces. To assess, quantitatively, the aerodynamic impact of corrugated chord profiles, we created a smoothed wing model by fitting quadric curves through each measured cross section (figure 2*b*). The force coefficient comparisons of smoothed and full-fidelity, corrugated wings are shown in figure 2*c,d*. The corrugated wing generates marginally higher force coefficients than the smoothed wing at all angles of attack up to 10°, whereupon the corrugated wing performs better owing to more gradual stall characteristics (figure 2*c*). This angle of attack may be higher than dragonflies naturally use when gliding, but this feature could improve stability during flapping flight. The maximum lift-to-drag ratio is slightly lower for the corrugated wings (3.38 and 3.23 at 10.4°; figure 2*d*).

To investigate the effects of corrugation further, we performed CFD simulations on wings with exaggerated or reduced corrugation amplitude. Subtracting the smoothed surface from the full-fidelity model removed the effects of twist, camber and bending, leaving a planar wing with corrugated relief. We find that varying the corrugation amplitude has little effect on lift generation at angles of attack less than 5° but, at higher angles, lift force decreases when the amplitude is reduced *or* enhanced, i.e. the naturalistic profile performs better than flat or highly corrugated profiles. Drag, however, increases monotonically with corrugation (figure 2*e*). The result is a diminishing lift-to-drag ratio with increasing corrugation depth. Notably, the naturalistic corrugation depth does not give rise to the dramatic decrease in lift-to-drag ratio we observe for the large amplitude corrugations (figure 2*f*). As such, natural-scale corrugations increase resistance to bending loads without greatly increasing material volume or compromising torsional stiffness [21–23,34], but we conclude that this is not offset by a substantial aerodynamic cost, and may even lead to greater aerodynamic efficiency by enabling higher aspect ratio geometries.

**Figure 3.**
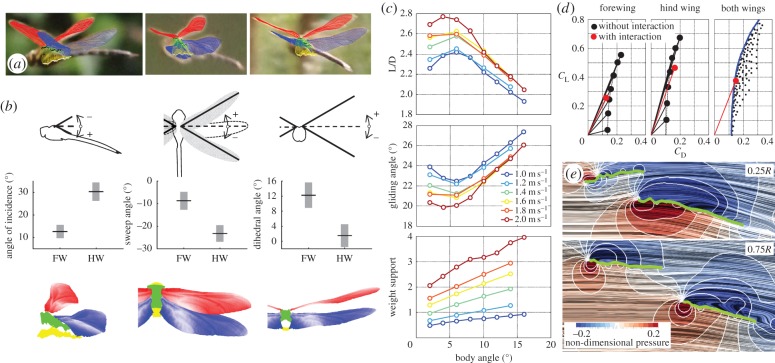
Wing angles and aerodynamic performance of a gliding brown hawker dragonfly, *Aeshna grandis*. (*a*) Three-dimensional models of the forewing (red), hindwing (blue), and the upper (green) and lower (yellow) surfaces of the thorax fitted to photographs of a gliding dragonfly taken in the field. (*b*) Definition of angle of attack, sweep angle and dihedral angle, and the mean ± s.d. of the fitted wing angles (*n* = 32). Side, top and back views of the mean wing position are shown in the lower panel. (*c*) Lift-to-drag ratio, glide angle and weight support of the dragonfly model at multiple body angles and speeds (fore- and hindwing grids: 301×321×61, global body grid: 151×201×91). (*d*) Lift and drag coefficient polars of the fore- and hindwings with (red) or without (black) aerodynamic interactions at 1.4 m s^−1^. The body angle is set at 2° to match lift with the weight measured from specimens caught at the same location. The blue line indicates the performance limit of the fore- and hindwings combined without aerodynamic interactions. (*e*) The two-dimensional flow structure shown by line integral convolution (LIC) streamlines and pressure distribution contours around the fore- and hindwings at 25% and 75% of wing length. The positive and negative pressure regions of each wing connect with each other, revealing an aerodynamic interaction between the ipsilateral wing pairs.

In gliding flight, the fore- and hindwings do not operate independently but interact with one another. We manually fitted our measured wing planforms to 32 photographs of gliding *Aeshna grandis* taken in the field on a windless day to determine the angle of attack, sweep and dihedral angles of the wings relative to the body and camera (figure 3*a,b*). The absolute angles and the speed of flight remained unknown, so we performed simulations at six speeds between 1 and 2 ms^−1^, with body angles ranging from 2° to 16°. Multiple solutions were found that could support the body weight of captured conspecifics, between a body angle of 2° travelling with an airspeed of 1.4 ms^−1^ and a body angle of 1° at 1 ms^−1^ (figure 3*c*). At these values, we predict modest glide angles of 22–27°, comfortably within the range observed previously for *Sympetrum sanguineum* [35]. Using the lower body angle values, we calculated lift and drag polars for the fore- and hindwings with or without their contralateral partner (figure 3*d*). The forewing sits in a region of positive pressure generated by the hindwing and therefore experiences reduced drag; conversely, the hindwing suffers higher drag owing to the forewing (figure 3*d,e*). To explore this relationship further, we defined a limiting envelope (figure 3*d*: blue line) of fore- and hindwings without aerodynamic interaction based on multiple possible combinations of lift and drag of each wing (figure 3*d*, black dots). Combined aerodynamic performance is relatively good, especially in terms of the low drag, as a consequence of the wings' high aspect ratios. Although it is not desirable to place the two wings too close together (because the effective aspect ratio decreases), *A. grandis* keeps the performance of each wing high by trimming the wing angles to glide efficiently (red dot in figure 3*d*). In conventional, fixed-wing aircraft, high aspect ratio wings achieve better lift-to-drag ratios at the cost of manoeuvrability. In §4, we see how the Odonata overcome this trade-off by operating their four wings independently, achieving excellent flight performance.

## 4. Flapping flight aerodynamics

The flight style of modern Odonata is likely to be similar to that of Palaeozoic insects because of the striking morphological similarities of the flight apparatus and other features that suggest a dependence on aerial predation—for example, having spines on the forelegs. Despite the retention of an ancestral-like state, having four independently driven flapping wings puts the Odonata in the minority of extant insects. They have the ability to modify the phase of their wing strokes, and the aerodynamic consequences of doing so has been examined in some detail. The consensus is that counter-stroking is used during cruising flight, whereas the wings operate in-phase during high acceleration manoeuvres but at the expense of power economy [36–42]. In common with many insects, the Odonata are incapable of supporting body weight using the sum of their four wings' maximal aerodynamic force coefficients under steady-state conditions [43]. Consequently, they use flow patterns associated with remarkably high lift force coefficients, where the sharp leading edge of the wing causes the airflow to separate from the surface and reattach further back along the chord [44–52].

Thomas *et al.* [46] filmed freely flying Anisoptera flapping their wings most commonly out of phase, with a leading-edge vortex on the forewing and attached flow on the hindwing. When flapping in-phase, they exhibited separated flow at the leading edge of the forewing, creating a separation bubble defined by an enclosing streamline that reattached on the hindwing, delineating a very large leading-edge vortex over the wings as they acted as a single aerodynamic surface. This flow topology is likely to be associated with very large lift force coefficients [46]. The energetic consequences of the interaction of the fore- and hindwings are still controversial. Lan & Sun [53] showed that flapping in phase can enhance vertical and total force, whereas a 90° phase shift enhances horizontal (thrust) force at the expense of total force. Under certain kinematic conditions, counter-stroking minimizes power requirement, because each wing travels upwards in the upwash of the other, whereas in-phase kinematics maximize the force produced [54]. Conversely, while some simulations have shown that forewing–hindwing interaction reduces force generation across a range of flight speeds [55], economy could be enhanced. This happens either by reducing wasteful swirl in the wake through the interaction of the hindwing with the wake of the forewing [56], or by tuning the hindwing kinematics to pass near to the leading-edge vortex shed from the forewing, harvesting energy from the wake in a beneficial manner [46].

Aerodynamic computational or physical models of flapping flight rely heavily on the quality of morphological and kinematic data. The earliest dragonfly kinematics were described by Magnan [57] and Chadwick [58], who both used high-speed cinematography to determine frequencies and amplitudes. Other optical methods, such as stroboscopes, have been used latterly to acquire slightly more quantitative data [59]. In recent times, kinematics have been measured in increasing detail using a variety of methods from simple high-speed video [41], to projected comb-fringe techniques combined with natural landmarks on the wing used to estimate twist and camber [60]. Automated surface acquisition has also been developed to estimate twist and camber from the residuals of a fitted flat surface [61]. Kinematic data have been used to inform numerous physical and computational models where real or artificial wings are driven in their naturalistic configuration [56,62–68] or in parameter sweeps around key flight modes, such as hovering. For example, Young *et al.* [69] showed that force economy was enhanced under the observed values of flapping amplitude for *Aeshna juncea*.

Richer kinematic data have also elucidated the importance of flapping with a stroke plane that is inclined relative to the ground. With inclined stroke planes, the lift-to-drag ratio fails as a simple measure of efficiency, because aerodynamic drag, rather than lift, is used to support up to three quarters of the insect's weight [70]. Furthermore, the mechanical power required to pitch the wing in readiness for the next half stroke is reduced, because the added mass of air entrained by the wing is sufficient to rotate the wing around its long axis. Because wing rotation is largely passive, the musculature used to control the wing pitch is likely to be primarily used for tuning angle of attack, rather than being the primary driver of the wings' attitude [71].

Here, we measured the flow fields directly using time-resolved stereo particle image velocimetry (stereo-PIV [72]) during free flight. In so doing, we circumvented the difficulties of accurately acquiring kinematics, simulating flows and then providing validation for those simulations. Our goal was to verify the flow patterns observed qualitatively by Thomas *et al.* [46] using stereo-PIV to give an instantaneous measurement of the flow field [44,73]. Using the resultant velocity field, we aimed to calculate flow derivatives and test the importance of the leading-edge vortex's contribution to weight support in free flight. A secondary objective was to measure spanwise flow along the vortex core axis. Several studies cite the draining of vorticity into the wing tip vortex by means of axial flow as being crucial for leading-edge vortex stability throughout the half-stroke, whereas others have observed the phenomenon but questioned its importance.

Darters, *Sympetrum striolatum*, and hawkers, *Aeshna mixta*, were caught in the field and transported to the laboratory in envelopes to prevent wing damage. There, they were put close to ice until quiescent, then placed on a perch in the test section of a wind tunnel [46] parallel with a longitudinal (streamwise) vertical sheet of pulsing laser light directed onto the fore and hindwings (cf. [44,73]). The laser was activated and they launched from the perch after a period of warming, during which the subjects often fluttered their wings with shallow amplitude to warm the flight motor. The field of view was sufficiently large to capture several wingbeats after take-off, and the subjects were more or less aligned with the freestream with their wings entering the light sheet on each stroke. Flow fields were processed with respect to the freestream with the leading-edge vortex core manually identified at each time step, if present. These digitized points were used to objectively determine the vortex core diameter, axial velocities, tangential velocities and circulation. The diameters were determined from inflection points in the velocity profiles along radii normal and parallel to the wing chord; these points were also used to calculate tangential velocities. Circulation was calculated as *Γ* = *πdv* (where *d* is the mean diameter of the core and *v* is the mean tangential velocity at the edge of the core). The sectional lift attributable to the leading-edge vortex is calculated as *L'* = *ρUΓ* (where *ρ* is air density, 1.225 kg m^–3^ and *U* is the effective wing velocity) [74]. We measured the position of the wing cross sections by image analysis (thresholding the bright portion of the wing struck by the light sheet) and manually digitized the position of the wing hinges and wing tips in each frame to determine the spanwise location of the measurement plane. Despite operating at 1 kHz, sufficient for time-resolved data (where the acquisition frequency is high in relation to the wingbeat frequency), the specific protocol and apparatus limited our analyses to portions of the wing stroke cycle where the wing was broadly horizontal. If the wing tip was elevated much higher, then the wing itself obscured the flow over its upper surface; much lower and the background behind the flow over the wing became dominated by the body.

**Figure 4.**
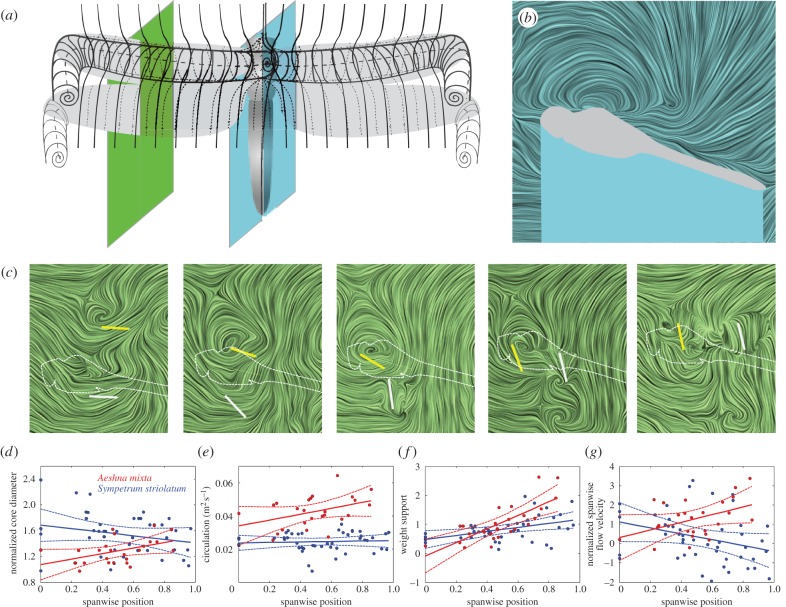
Flapping wing aerodynamics of a dragonfly with a leading-edge vortex over the forewings and thorax and attached flow over the hindwings. (*a*) Topology of the leading-edge vortex of dragonfly as described by Thomas *et al*. [46]; (*b*) cross section of the flow at the centreline of the body measured by PIV, with instantaneous streamlines visualized by LIC; (*c*) time-course of the measured flow field around the fore- (yellow) and hindwings (grey) with the laser sheet incident at approximately 45% of the wing's length from hinge to tip. The leading-edge vortex on the forewing is observed clearly, while the flow remains attached to the hindwing. (*d–g*) Spanwise distribution of (*d*) the core diameter of the leading-edge vortex normalized by the mean chord length (*A. mixta*, *AM*: *p* < 0.05, *R*^2^ = 0.22, *S. striolatum*, *SS*: *p* = 0.2, *R*^2^ = 0.06), (*e*) the circulation of the leading-edge vortex (*AM*: *p* < 0.05, *R*^2^ = 0.16, *SS*: *p* = 0.7, *R*^2^ = 0.004), (*f*) the contribution to weight support by the leading-edge vortex, defined by the ratio of the weight and the lift based on the sectional lift multiplied by the wing length (*AM*: *p* < 0.005, *R*^2^ = 0.57, *SS*: *p* < 0.005, *R*^2^ = 0.21), and (*g*) spanwise flow velocity normalized by the tangential velocity of the leading-edge vortex (*AM*: *p* < 0.05, *R*^2^ = 0.19, *SS*: *p* < 0.05, *R*^2^ = 0.10). The flow data in (*b*) and (*c*) are from the sequence of *Sympetrum striolatum*, whereas (*d–g*) are from *Aeshna mixta* (red) and *Sympetrum striolatum* (blue).

Qualitatively, our visualizations confirm the description of the flow topology shown by Bomphrey *et al.* [45] and described in detail by Thomas *et al.* [46], where counter-stroking kinematics lead to a cylindrical leading-edge vortex spanning the thorax from forewing tip to forewing tip and the hindwing exhibits attached flow (figure 4*a*). To this pattern, we can add quantitative data from 69 recordings of four *Sympetrum striolatum*, and two *Aeshna mixta* individuals, enabling the calculation of leading-edge vortex circulation and hence its contribution to weight support. For both species, we find that the core diameter is substantially greater than the mean chord length of the forewings at all spanwise positions from the centreline (figure 4*b,d*) to the wing tips (figure 4*c,d*). The dataset comprises a range of flight behaviours (side slip angles, vertical accelerations, etc.) and are, consequently, somewhat noisy. Nevertheless, it can be seen that the diameter (figure 4*d*) and circulation (figure 4*e*) increase from root to tip in *Aeshna* but not in *Sympetrum*. The spanwise contribution to weight support (figure 4*f*) increases from root to tip in both species, but more markedly for *Aeshna*. Both species are approximately capable of supporting their weight by the contribution of the forewing leading edge vortex alone; mean normalized weight support is L/W = 0.82 for *Sympetrum* and L/W = 1.04 for *Aeshna*. Spanwise flow along the axis of the leading-edge vortex core has been discussed extensively in recent times [46,48,50,73,75–86]. Our measurements show that axial velocities can be quite strong in either direction (figure 4*g*), at least during slow forward flight, and confirm that axial flow is not, therefore, an essential prerequisite of vortex stability during the period of a single half stroke [46,73,75].

## 5. Estimates of span efficiency from wake measurements

Quantitative flow visualizations can also be used to estimate the efficiency with which lift is generated. The span efficiency is the ratio of the power required to generate lift under ideal aerodynamic loading conditions on the wing to the power required in reality: the ideal power divided by the induced power. It can be measured empirically as the deviation of the downwash velocity profile behind the wings from the theoretical ideal of an even distribution across the span [87,88]. Several insects, birds and bats have been assessed using transverse PIV measurement of the wake during wind tunnel experiments (reviewed in [44]). Because (i) the downwash velocity is dependent on the spanwise lift distribution, (ii) lift is proportional to the product of the lift coefficient and its velocity, and (iii) the velocity of root flapping wings increases linearly with distance from the wing hinge, we can hypothesize that flapping wings can improve span efficiency if the wing is broad at the root and tapers towards the tip. Under those conditions, the diminishing chord length counteracts the increase in local velocity, acting to equalize the loading distribution along the wing. Anisoptera have wing planforms consistent with this hypothesis (essentially outward pointing triangles); however, Zygoptera have wing shapes that are petiolate, with chord lengths that lengthen towards the wing tip. Consequently, the Zygoptera are predicted to perform less well than the Anisoptera in terms of span efficiency, because there will be little lift generated proximally and considerable lift generated distally, whereas the Anisopotera will generate lift with more consistent magnitude across the span. We can test this simple prediction by correlating span efficiency with taper ratio, the ratio of chord lengths at the 20% and 80% (semi) wing radius, where Zygoptera ratios are less than unity but Anisoptera are greater.

Here, we report span efficiencies for six species of Odonata (three hawkers, one darter and two damselflies). These are the first insects to be assessed for span efficiency during free flight. Individuals were chilled near ice until quiescent for varying lengths of time depending on size. They were allowed to perch in the wind tunnel upstream of the transverse PIV laser plane at distance that prevented the abdomen from touching the light sheet during take-off (25–90 mm). Once the individuals had warmed their flight motor by shivering, they took off into the light headwind and PIV measurements were acquired by post-triggering cameras operating at 1 kHz following the protocol of Henningsson & Bomphrey [89]. The wind tunnel speed was set according to the species-specific preferred flight speed, as measured in our standardized indoor arena (§6). We recorded post-take-off flight sequences from 24 individuals: *Anax imperator* (*n* = 1), *A. grandis* (*n* = 1), *Aeshna mixta* (*n* = 3), *Sympetrum striolatum* (*n* = 5); *Calopteryx splendens* (*n* = 7) and *Enallagma cyathigerum* (*n* = 7). From 212 recorded sequences, 73 were processed, with the remainder discarded owing to highly asymmetric flight paths, wing injuries or the subject passing through the light sheet. In summary, hindwing tip vortices were identified manually, downwash profiles were extracted between these, and span efficiency was calculated for 8629 vector fields with sequences typically lasting several wingbeats within 118 ± 41 images (and hence milliseconds).

**Figure 5.**
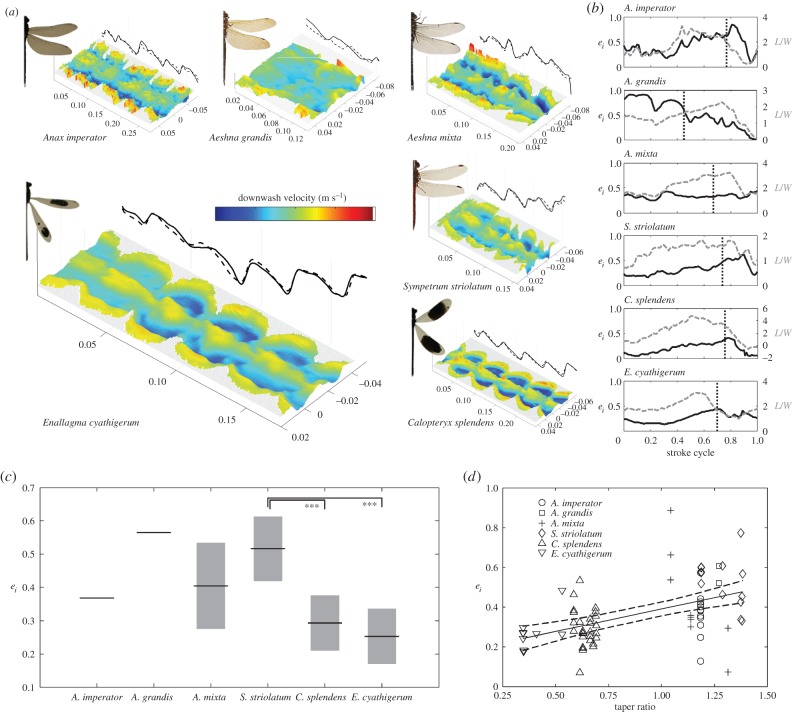
The wake behind six species of Odonata and their span efficiency. (*a*) Example sequences of the time-resolved induced downwash of each of the six species. Both the relief and colour represent downwash velocity, with shades in blue/cyan representing downward velocities corresponding to positive lift and shades in red/yellow upward velocities corresponding to negative lift. The ranges of the colour bar are scaled to [−1 1] (m s^−1^) for the Zygoptera and [−2 2] for the Anisoptera. Note the substantial upwash from wing root vortices close to the centreline of the petiolated Zygoptera, but a more consistent downwash profile across the span in the Anisoptera. Solid and broken lines projected onto the far side of the plot show the vertical excursion of the two tip vortices from hindwing and thus the wing stroke cycle. (*b*) Time series of the span efficiency (black) and weight support (grey) through the ensemble-averaged wingbeat of each species beginning at pronation of the hindwings. Vertical dotted lines show the transition from downstroke to upstroke. (*c*) The span efficiency of each species. Boxes show median values with 95% confidence intervals. *Post hoc* pairwise ANOVA under Tukey criterion shows difference between *Sympetrum striolatum* and two of the Zygoptera are significant (*p* < 0.001). (*d*) The taper ratio is positively correlated with span efficiency (*p* < 0.001, *R*^2^ = 0.24). Solid and dashed lines show the least-squares regression slope with 95% confidence intervals.

Figure 5*a* shows a time series of transects through the downwash at 1 ms intervals for representative examples of each species. The colour and relief show the magnitude of the downwash velocity behind the trailing edges of the hindwings, black solid and dashed lines show the vertical excursion of the undulating left and right hindwing tip vortices throughout the sequence. Calculated weight support [89] throughout the wingbeat reveals a mean normalized weight support across all species of L/W = 1.86 ± 0.84, reflecting net upward forces in flight that occur shortly after take-off. Ensemble-averaged temporal variation in span efficiencies are shown in figure 5*b*, with mean values ranging from *e_i_* = 0.24–0.56 (figure 5*c*), slightly lower than previously reported for hawkmoths [89] or locusts [87]. Following previous work and hypotheses based on first principles, we tested the wing taper ratio, normalized lift (calculated lift/weight), wing loading and advance ratio in a multiple variable linear regression (IBM SPSS Statistics v. 22) with span efficiency as the dependent variable. In contrast to hawkmoths, Odonatan span efficiencies are not correlated positively with normalized lift or negatively with advance ratio [89], nor is there a significant relationship with wing loading. However, as predicted, the Zygoptera have the lowest span efficiencies, and span efficiency is strongly correlated with taper ratio (figure 5*d*: *B* = 0.23, *t* = 4.76, *p* < 0.001 after sequential removal of aspect ratio (*p* = 0.78), wing length (*p* = 0.78), advance ratio (*p* = 0.42), wing loading (*p* = 0.07), weight support (*p* = 0.08) and mass (*p* = 0.11) from the model), confirming the relationship between wing planform and aerodynamic efficiency during flapping flight.

The Zygoptera showed a mean *e_i_* = 0.36, whereas the Anisoptera showed a mean *e_i_* = 0.45 across all wingbeats, sequences, individuals and species. These values mean that, for the dragonflies to fly, they must generate 221% of the power that would be necessary to produce the same lift with perfect aerodynamic efficiency (i.e. from the ideal ‘actuator disc’ or ‘lifting line’). Damselflies, on the other hand, operating with wing shapes that are less efficient in terms of span efficiency must generate 275% of the power that would be required under ideal conditions. This result returns to an overarching question of why insect wing shapes are so variable, and there is a distinct lack of convergence on an optimal solution from the standpoint of aerodynamics. Clearly, there are both adaptive and non-adaptive factors that contribute to wing shape, only some of which will have any aerodynamic or mechanical relevance [90]. One possible benefit of the Zygopteran planform might be the movement of the centre of pressure away from the centre of mass. Thus, for the same wing area and wing mass, the wing beat frequency could be reduced, whereas the torques around the body become stronger. Alternatively, the number of chord lengths swept by the most aerodynamically important regions of the wing could be increased, changing the flow characteristics and the time history of force generation [82,84,85,91,92], expanding the kinematic envelope available for manoeuvres. These speculations await rigorous testing.

In our quantitative longitudinal and transverse flow visualizations described in this section and §4, our technique of choice was stereo-PIV. We chose stereo-PIV because it is fast to set up (important if you wish to fly the same experimental subjects in both configurations), quick to process and simpler to analyse. We were confident that the acquisition frequency was sufficiently high that we would not miss major flow features and that the gap between the subjects and our measurement plane was sufficiently small that major deformations of the wake would be minimal. In future work, however, we expect that a fully volumetric approach to fluid measurements will provide the most comprehensive datasets. Volumetric or tomographic PIV (tomo-PIV) has been used recently to measure the wakes of insects in tethered flight with promising results [93,94], but the technique is yet to be applied to the fluid mechanics of free flight.

## 6. Flight performance and behaviour

Extensive musculature, complex wing architecture and aerodynamic mechanisms combine to propel insects along three-dimensional trajectories through space. Extreme manoeuvrability and agility, high top speeds and hovering flight are all signature behaviours in the repertoire of the Odonata. Field measurements are challenging to acquire and, whereas a small number of field studies covering multiple species do exist [39,95], the majority have been limited to wind tunnel experiments [40], laboratory environments [35] or controlled naturalistic environments where the subjects are sometimes coerced into hunting flight in the hope of soliciting near maximal performance [96–98]. Species diversity is often limited in these experiments.

It might be reasonable to assume that predatory flights will elicit near maximal performance, but this depends on the performance capabilities of the prey and it is quite possible that prey capture is relatively undemanding in comparison with migration, avoiding predation by birds, mate guarding or territorial battles with conspecifics. To provide standardized baseline data and to give suitable wind tunnel speeds for our aerodynamic measurements, we tracked nine species in a large 2 × 3 × 1.5 m flight arena, painted white around three sides, using calibrated stereo-cameras (following the protocol detailed previously [89,90]). Three-dimensional positional data acquired at 500 Hz were used to fit a quintic spline with a smoothing parameter based on autocorrelation of the residuals [99]. We do not expect this exploratory behaviour to exhibit the full repertoire of each species. In fact, it is clear that it will not because we observed very little hovering flight and the maximal speeds we recorded are below those reported in the wild. Nevertheless, the standardization of our method is useful for benchmarking a conservative flight performance envelope. Moreover, the modal speeds we observed are indeed the preferred speeds at which the dragonfly and damselflies chose to fly within that well-defined and repeatable setting. Here, we use these metrics to highlight coarse interspecies variability and provide data for future investigations into comparative flight performance.

**Figure 6.**
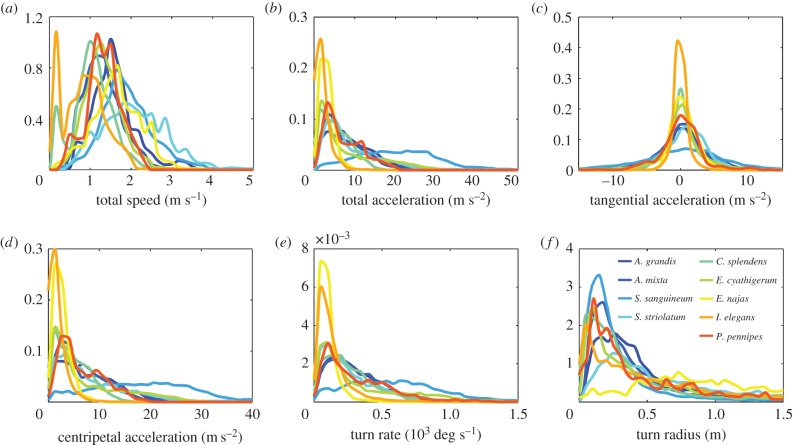
Flight performance in nine species of British Odonata flying in an indoor arena measuring 2×3×1.5 m. (*a*) Total speed; (*b*) total acceleration; (*c*) tangential acceleration; (*d*) centripetal acceleration; (*e*) turn rate and (*f*) turn radius.

Histograms characterizing the flight performance characteristics of nine British species are presented in figure 6. The Zygoptera tended to fly more slowly than the Anisoptera (*t*-test; *p* = 0.014; Anisoptera mean = 1.81 ± 0.29 m s^−1^; Zygoptera mean = 1.16 ± 0.31 m s^−1^), particularly the blue-tailed damselfly (*Ischnura elegans*) and the banded demoiselle (*Calopteryx splendens*), but the majority of species preferred to fly at between 1 and 2 m s^−1^ (figure 6*a*). Observed accelerations were relatively modest, with only the ruddy darter (*Sympetrum sanguineum*) frequently accelerating over 3*g* during turns (figure 6*b–d*). Turn rates (based on the trajectory of the individuals' centroid as opposed to rotations of the body axis) were typically 170 ± 110 deg s^−1^ although rates of 1000 deg s^−1^ were not uncommon in several species (figure 6*e*). Animals that are capable of hovering flight can show infinitely small turn radii; however, the modal turn radii that we observed were 0.29 ± 0.16 m as the subjects explored the arena (figure 6*f*).

## 7. Predatory and conspecific pursuit flight

Odonata are known for the exceptional flight performance that enables their predatory lifestyle. While many Zygoptera pluck their prey from solid substrates, the Anisoptera exclusively intercept flying insects on the wing. The Anisoptera can be further categorized into two types according to the foraging styles [100]: perchers and hawkers (or sometimes ‘fliers’). The medium/small perchers scan the sky for potential prey and ambush any flying insects within range. The generally larger hawkers patrol an aerial territory and initiate prey pursuits when appropriate prey are identified. In this section, we focus on the perchers, which are more convenient to study owing to their short-range pursuits and well-defined initial conditions. Depending on the species, perchers favour different perch locations and prey size when hunting [101]. Once an appropriate prey is spotted, the dragonfly launches itself into the air with acceleration of 1.52 ± 0.4*g* for *Libellula cyanea* [98], reaching a maximum speed of 2.28 ± 0.46 m s^−1^. Similarly, the slightly smaller *Plathemus lydia* accelerates at 1.25 ± 0.38*g* and reaches maximum speed of 2.15 ± 0.39 m s^−1^ (statistics from free foraging data in reference [96]). Most prey are acquired within 60 cm range [102], and we rarely observe evasive manoeuvres from the prey, because the dragonfly always approaches from the prey's visual blind spot (behind and below). During pursuit, the dragonfly can produce large lateral accelerations of 2.00 ± 0.57*g* and achieve tight turns with radius of curvature as small as 4.1 ± 2.4 cm [98]. Such capabilities exceed the flight performance of the typical prey [90], meaning that prey capture is predominantly a sensory challenge rather than an aerobatic dogfight.

**Figure 7.**
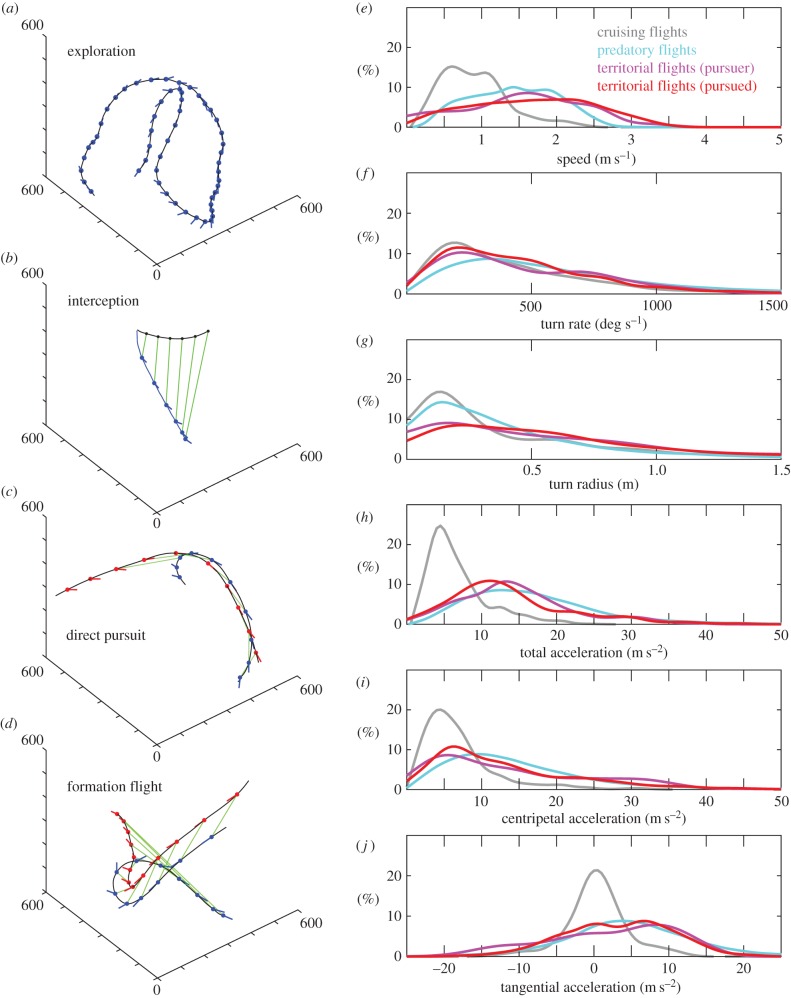
Flight performance during cruising, hunting and territorial flights. (*a*) Percher dragonfly *Plathemus lydia* performs low altitude cruising flight typical to territorial patrol and landscape exploration. These flights have an average speed<1 m s^−1^. (*b*) Predatory flights are represented by a characteristic interception trajectory with representative waypoints at 50 ms intervals. The dragonfly maintains position directly below the prey and achieves interception by increasing altitude. (*c*) During a territorial dispute, the pursuing dragonfly follows almost the exact same flight trajectory as the pursued dragonfly, separated by approximately 50 ms (green baselines connecting the waypoints at 50 ms intervals). (*d*) In other instances, territorial flights resemble formation flight, with the pursuing dragonfly escorting the pursued dragonfly on the side. (*e*) The speed distributions of different flight modes show that both predatory and territorial flights require significantly higher flight speed than typical cruising flights. The highest speeds we observed occurred during conspecific chases, with average speeds during these events of 1.60 m s^−1^ and the maximum reaching 3.57 m s^−1^. (*f*) Territorial flights share a similar turn rate distribution with cruising flights, but the modal rate doubles during predatory flights. (*g*) Predatory flights also require tighter turns compared with territorial flights. Territorial flights typically have larger turn radius, consistent with more direct flights out of the territory. (*h*) Predatory and territorial flights also require higher accelerations than cruising flights. (*i*) Such increase of total acceleration can be attributed to the overall increase of centripetal acceleration for turning. (*j*) Given the nature of aerial pursuit, the tangential acceleration also shifts from symmetric distribution as in cruising flights to predominately forward acceleration during predatory and territorial flights.

Here, we present new data from the indoor dragonfly flight arena at the Howard Hughes Medical Institute Janelia Research Campus showing quantitative differences in flight performance during cruising, predatory and territorial escort flights (figure 7). Experiments and kinematics data acquisition were performed, using the protocol described recently [96]. To summarize, freshly emerged wild dragonflies were kept in a custom dragonfly arena (5.5 × 4.3 × 4.6 m) with naturalistic lighting, temperature, humidity, visual texture and a large number of fruit flies. The dragonflies live and forage freely in this room for up to two weeks. A miniature carbon fibre frame of three-dimensional tracking markers was mounted on selected dragonflies to allow precise reconstruction of the flight path and body orientation. During typical exploratory cruising flight, *Plathemus lydia* follows sinuous and relatively slow flight paths (figure 7*a*). During predatory flights, it exhibits the short characteristic interception trajectory (figure 7*b*). When engaging in territorial defence, the pursuer sometimes adopts a direct pursuit strategy which closely matches the flight trajectory of the intruder (figure 7*c*). At other times, the trajectories resemble formation flight (figure 7*d*). The exact goal of the territorial chase is still under investigation, but the chase is usually aborted as soon as the conspecific leaves the territory. Unsurprisingly, the observed performance envelope expands during prey interception and territorial flights. From the speed distribution in figure 7*e*, it is immediately clear that territorial flight ranks as the most demanding task (pursuer and pursued combined mean 1.60 ± 0.81 m s^−1^; maximum 3.57 m s^−1^), prey interception flights are the second most demanding (mean 1.39 ± 0.52 m s^−1^; maximum 2.44 m s^−1^), and cruising flights are the most leisurely (mean 0.98 ± 0.43 m s^−1^; maximum 2.41 m s^−1^). However, intercepting small prey still requires more frequent tight turns than in territorial flights as the turn rate is slightly greater and the turn radius slightly shorter (figure 7*f,g*). This difference is reflected in the acceleration distribution in subtle ways. For instance, territorial flights involve slightly less total acceleration between 20 and 30 m s^−2^ but they do push the dragonfly to similar maximum acceleration over 40 m s^−2^ (figure 7*h*). During prey interception flight, the centripetal acceleration is always non-zero (figure 7*i*), whereas in territorial flight, we observed almost straight sections of trajectories with zero centripetal acceleration (figure 7*i*). In general, during territorial flight, the pursued dragonfly tends to have smaller total acceleration but higher centripetal acceleration. While tangential acceleration is symmetric and tightly clustered around zero for cruising flight (indicating equal amounts of modest acceleration and deceleration), both prey interception and territorial flights require more substantial accelerations (figure 7*j*).

## 8. Prey interception and target foveation

The percher dragonflies have impressive prey capture success rates from 83% to 97% [98,103] as observed in the field and in the greenhouse laboratory environment. One key to efficient prey capture is the aerial interception strategy. Instead of tracking the observed location of the target such as houseflies [104] and tiger beetles [105], dragonflies intercept prey at the expected future location [103,106] (figure 7*b*). The flight trajectories resemble the implementation of proportional navigation in which the target retinal position is maintained constant [106]. Recent detailed trajectory analyses add amendments to this description [96]. For instance, even though dragonflies can fly sideways and backwards, biomechanical constraints only allow the dragonfly to fly at maximum speed in the forward direction. As a result, the dragonfly invariably reorients itself early in the predatory flight, regardless of the interception strategy. Through analysing hundreds of independent prey capture events, it was concluded that the interception trajectories could resemble proportional navigation just as well as many other guidance strategies such as parallel navigation. In fact, the dragonfly appears simply to align its body to the prey flight direction and keep the target within an approximately 50° cone directly overhead [96]. This interception strategy simplifies the task to two-dimensional tracking in the zenith direction and the dragonfly must only increase its altitude to achieve interception.

Prior to prey pursuits, *Plathemus lydia* dragonflies often perform a rapid head movement to centre the target in its dorsal fovea [103]. It was proposed that such head movement, together with some thorax translation, produces sufficient motion parallax for target distance estimation [103,107]. However, the fact that some pursuits were not preceded by significant head movement [103] and that the head movements produce little translation means that parallax target ranging is questionable. Instead, this head movement has a pure foveation function and is triggered as the target enters a specific visual receptive field (H-T. Lin 2013, unpublished data). Foveation is maintained during pursuit flight [108] with minimal time lag (approx. 4 ms), signifying the presence of predictive control [96]. Further analyses of the three-dimensional head orientation during pursuit revealed that such predictive control cancels prey drifts owing not just to the dragonfly's in-flight body rotations, but also the prey drift owing to relative translation. This suggests that the dragonflies not only have a forward model of their own flight manoeuvres, but also a prey state estimator that extrapolates prey motion relative to self-motion during pursuit [96]. These internal models perhaps dominate the entire prey interception event, which typically lasts no more than 400 ms: a blink of a human eye.

## 9. Structure of the compound eye and target detecting neurons

Prey interception is a visually guided behaviour and the Odonata have among the best visual acuities of all the arthropods. At the centre of the dorsal fovea, the nominal angular resolution (interommatidial angle) can be 0.24° for the dragonfly [109], 20 times better than the fruit fly (approx. 5° [110]) and 10 times better than the mouse (0.49 cycles per degree) [111]. Indeed, Anisopterans such as *Plathemus lydia* typically pursue prey occupying a visual angle from 0.18° to 0.82°. At the third visual neuropil, lobula, a class of neurons selectively responds to small moving targets [112]. These small target motion detectors (STMDs) give peak responses to targets occupying less than 3° (1–2 ommatidia in most part of the compound eye) and exhibit direction selectivity. Their output structure overlaps with the input structure of the target selective descending neurons (TSDNs) which carry target movement information from the visual system in the head, through the neck, to the thorax [113]. Although the direct connection between STMDs and TSDNs is yet to be demonstrated, TSDNs encode qualitatively similar visual information as STMDs except, perhaps, with higher specificity. Indeed, the eight pairs of identified TSDNs can precisely encode target positions [114] and target directions via population coding [115]. TSDNs are the largest neurons passing through the pin-size neck joint of the dragonfly. Indirect evidence shows that these giant neurons drive the wing steering muscles [116]. Recent anatomical evidence suggests that TSDNs wrap around the output structure of the wing motor neurons and also form possible connections to the neck motor units (I. Siwanowicz 2015, personal communication). In summary, the target information is probably computed at the lamina–medulla level and integrated in STMDs in the lobula. TSDNs then relay the key target parameters to the wing and neck motor systems to coordinate the motor activities necessary to initiate and execute prey pursuit behaviour. Ongoing effort uses an ultra-light neural telemetry system to monitor TSDNs and flight motor units during prey interception. By integrating these neural data with our understanding of the flight kinematics and aerodynamics, we can start to tell the full story of sensory encoding, motor control, biomechanics and behavioural strategies.

## 10. Concluding remarks

We have shown the state of the art in Odonatan flight biomechanics by describing several recent experiments, each contextualized by a series of very brief reviews. The scope of contemporary experimental biomechanics is extremely wide ranging. In this work, we have presented data that could only be acquired using an extensive suite of equipment and methodologies, including a specialized wind tunnel, two free flight arenas, high-speed stereo-photogrammetry, a customized motion capture system and PIV apparatus. We have accurately measured the complex wing surface topographies by laser scanning many representatives from a museum collection and fitted those shapes to photographs taken in the field in order to ascertain the wing angles crucial to our gliding study. Empirical measurements and extensive computational simulations were evaluated within the frameworks of trajectory analysis, guidance and control, neurophysiology and aerodynamic theory.

We have identified that structural corrugations do not significantly impact the aerodynamic performance of dragonfly wings up to, and including, naturally occurring angles. Corrugations begin to incur substantial drag costs if the angles become too high, but natural corrugations can help to smooth stall characteristics at high angles of attack. We have determined the costs and benefits of ipsilateral wing aerodynamic interactions during gliding flight, and quantified the contribution that the forewing leading-edge vortex makes to weight support during typical flapping flight. Moving on from force generation, we have assessed the efficiency with which those forces are generated by measuring the span efficiency of six species, finding that wing planform is correlated with the induced power of flapping flight. As predicted from first principles, wings tapering from root to tip outperform petiolate wings by equalizing the downwash distribution across the span. Finally, we characterized the normal, exploratory flight performance of nine species in a flight arena, and have shown, for one species, how the performance envelope expands when operating in different flight modes: cruising, hunting and territorial chasing. By concentrating on the same species wherever possible, we have been able to offer a synthesis of the datasets, assessing our findings to deliver a coherent picture of the mechanics of flight in the Odonata.

Moving forward, there are several key areas in Odonata flight research that we predict will advance our understanding of unsteady aerodynamics, flight control, sensory integration and the evolution of flight. Flight is arduous, and a prerequisite of powered flight is energy management. The aerodynamics analyses have pointed to several features of flight economy, but these must be linked to the dragonfly's metabolic cost before we can draw any conclusions on flight strategies. This research direction would benefit from fresh input on the comparative physiology of flight muscle in a biomechanical and ecological context [117]. To characterize fully the aerodynamics of all the behavioural repertoire, we must exploit and develop new approaches that allow high-throughput, high-quality wing kinematics measurements [61,96,118,119]. Detailed characterization of the wing's powertrain has proven to be highly valuable for understanding the interplay of flight muscles and the wing hinge during flight [120]. Applying the same X-ray technique would be more challenging for Odonata. Instead, a combination of tethered flight and wireless recording of the flight motor and steering muscle activity would produce fruitful results.

Of course, to understand flight control, we must focus on sensorimotor transformation of the dragonfly as well as the functional morphology of the wing mechanics. To understand the sensory encoding of wing mechanosensors requires combining aerodynamics and wing mechanical properties. Currently, we do not yet have a suitable dragonfly wing model to characterize the deformation experienced by the mechanosensors, and nor do we have an adequate characterization of wing mechanosensor signals equivalent to those being described in moths [121]. To discover more about behavioural strategies, we must progress beyond the simple centre-of-mass trajectory analyses that have been performed predominately to date. The details of head angles, body orientation and posture often indicate the underlying mechanism of flight guidance and control. Additionally, the use of artificial targets with prescribed perturbation will allow us to disambiguate behavioural models by artificially eliciting predictable and repeatable flight responses. Finally, to generalize and validate flight strategies in the real world, field recordings are essential, although a reliable field data logger for Odonata is yet to be developed. Ancient dragonfly-like insects were the first animals to conquer the sky. Flight behaviour in extant species not only exemplifies the integration of aerodynamics, functional morphology and sensorimotor integration, it might very well hold the secrets to the origin of flight.

## Supplementary Material

Supplementary Figure 1: The three-dimensional wing surface topographies of 52 Anisopteran individuals collected by laser-scanning.

## Supplementary Material

Supplementary Data 1: Wing surface coordinates from 52 Anisopteran individuals collected by laser-scanning.

## Supplementary Material

Supplementary Data 2: Flight performance of nine species of British Odonata flying freely in an indoor arena.

## Supplementary Material

Supplementary Data 3: Grid convergence data.

## Supplementary Material

Supplementary Data 4: Leading-edge vortex raw data from Sympetrum striolatum and Aeshna mixta in support of figure 4d-g.

## Data accessibility

The datasets supporting this article have been uploaded as part of the electronic supplementary material.
