# Supplementary material for: Flight of the dragonflies and damselflies
Source: Philos Trans R Soc Lond B Biol Sci. 2016 Sep 26;371(1704):20150389. doi: 10.1098/rstb.2015.0389 (PMC4992713; doi:10.1098/rstb.2015.0389)
Supplement: Supplementary Figure 1: The three-dimensional wing surface topographies of 52 Anisopteran individuals collected by laser-scanning. [file rstb20150389supp1.pdf]

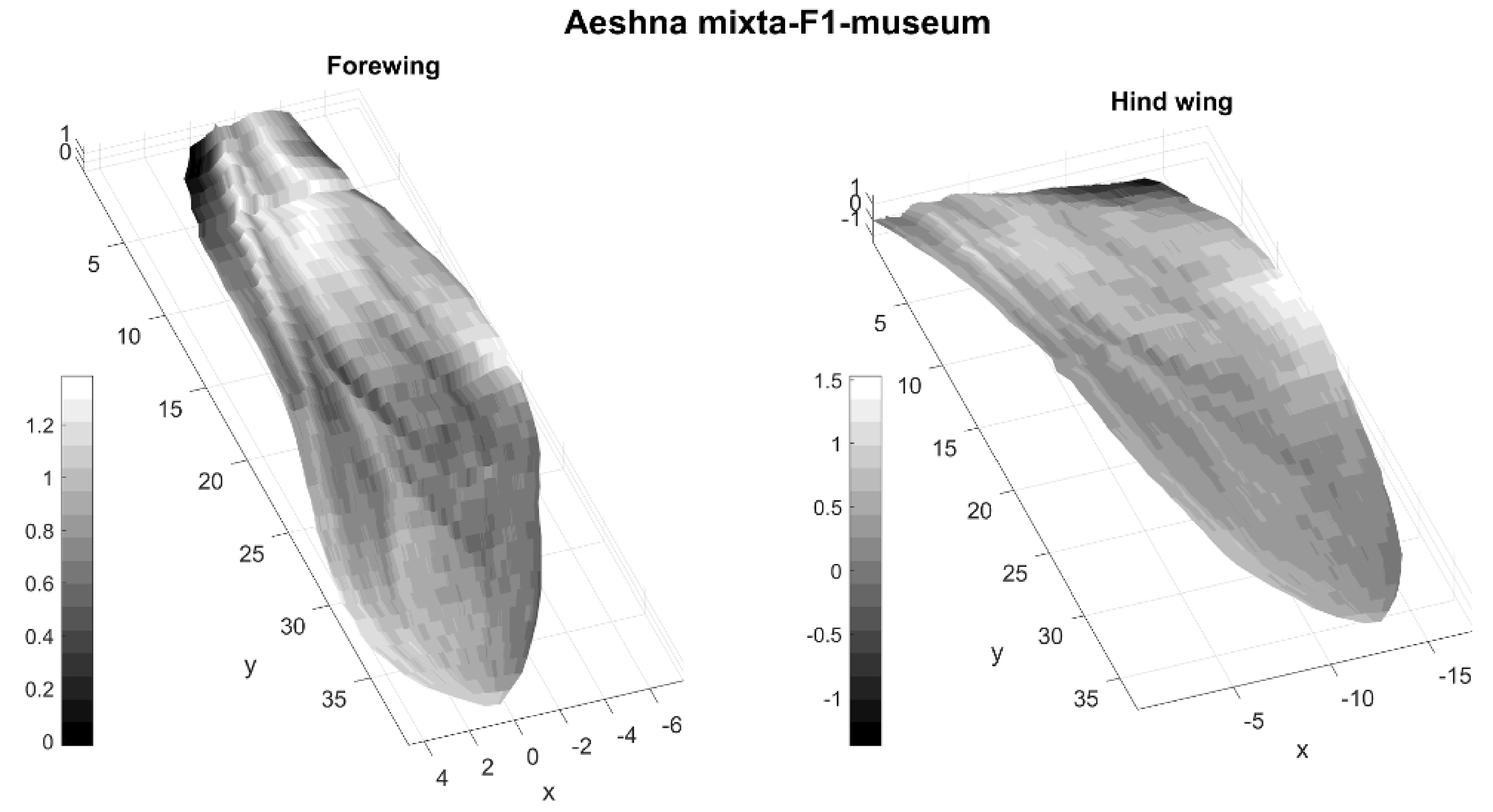
Aeshna mixta-F1-museum
Forewing
Hind wing
1
0
5
10
15
20
25
30
35
y
4
2
0
-2
-4
-6
x
1.2
1
0.8
0.6
0.4
0.2
0
1
0
-1
5
10
15
20
25
30
35
y
-5
-10
-15
x
1.5
1
0.5
0
-0.5
-1

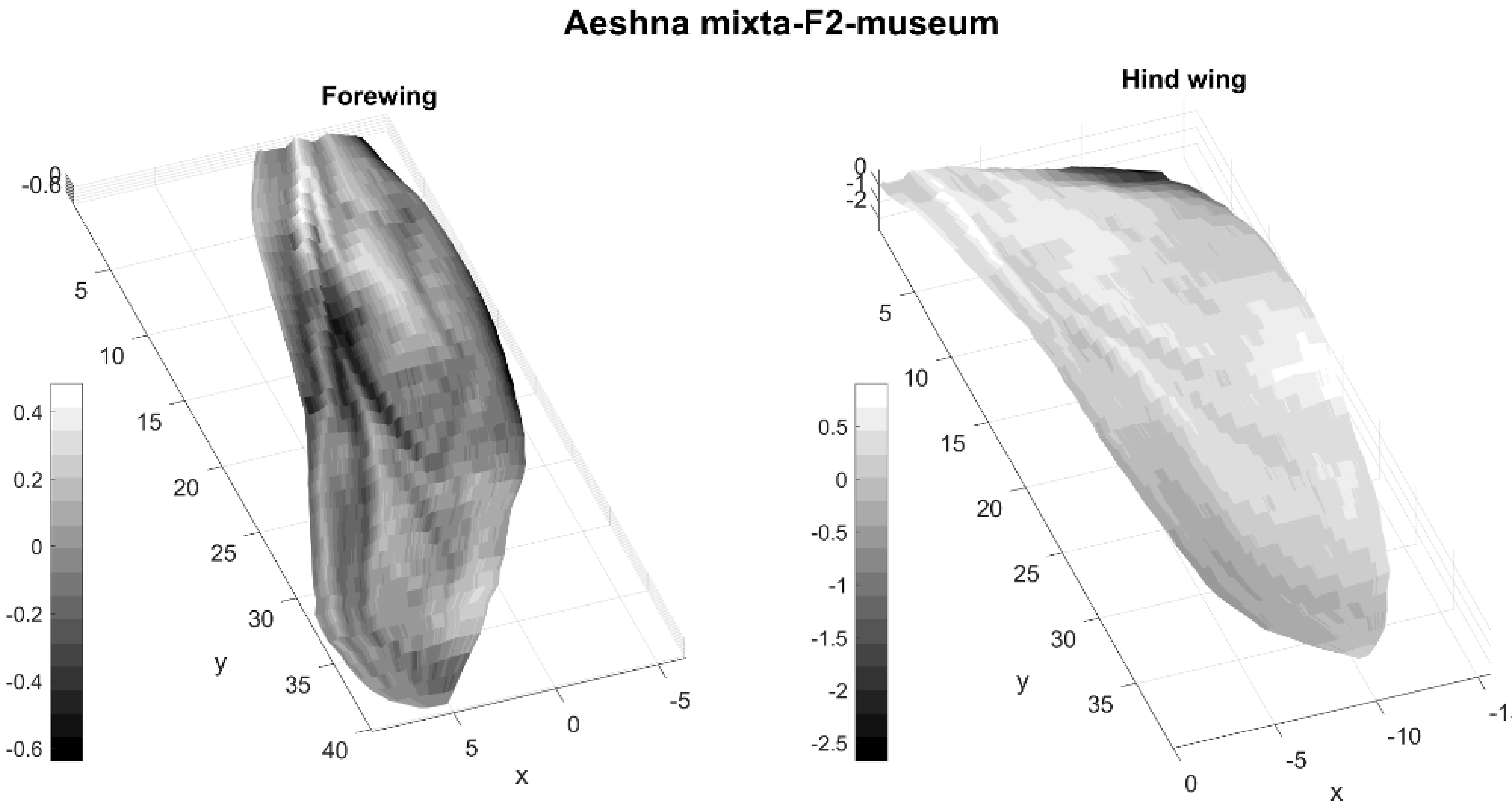
Aeshna mixta-F2-museum
Forewing
Hind wing
x
y

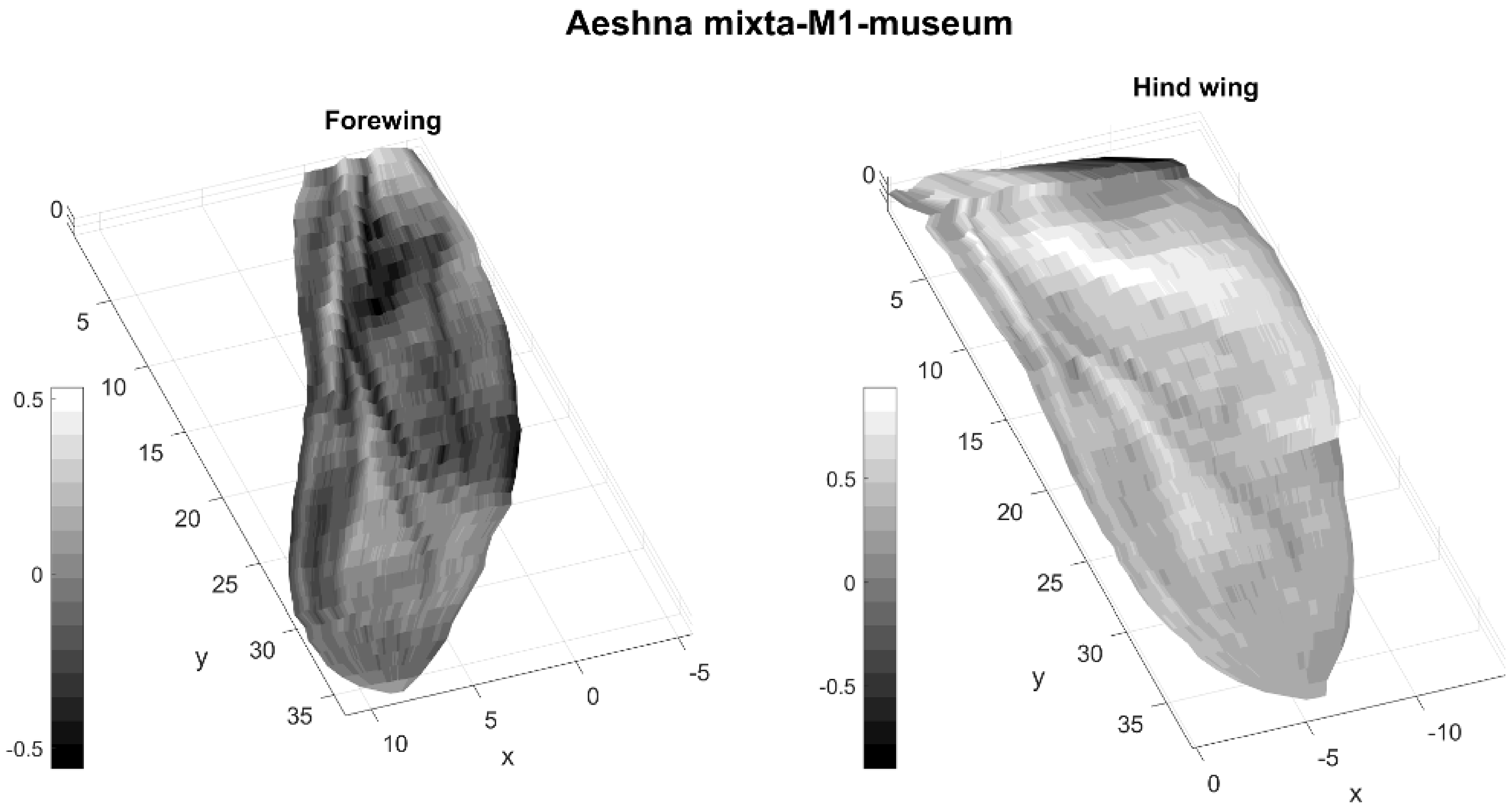

Aeshna mixta-M1-museum
Forewing
Hind wing
0.5
0
-0.5
0
5
10
15
20
25
30
35
y
10
5
0
-5
x
0.5
0
-0.5
0
5
10
15
20
25
30
35
y
0
-5
-10
x

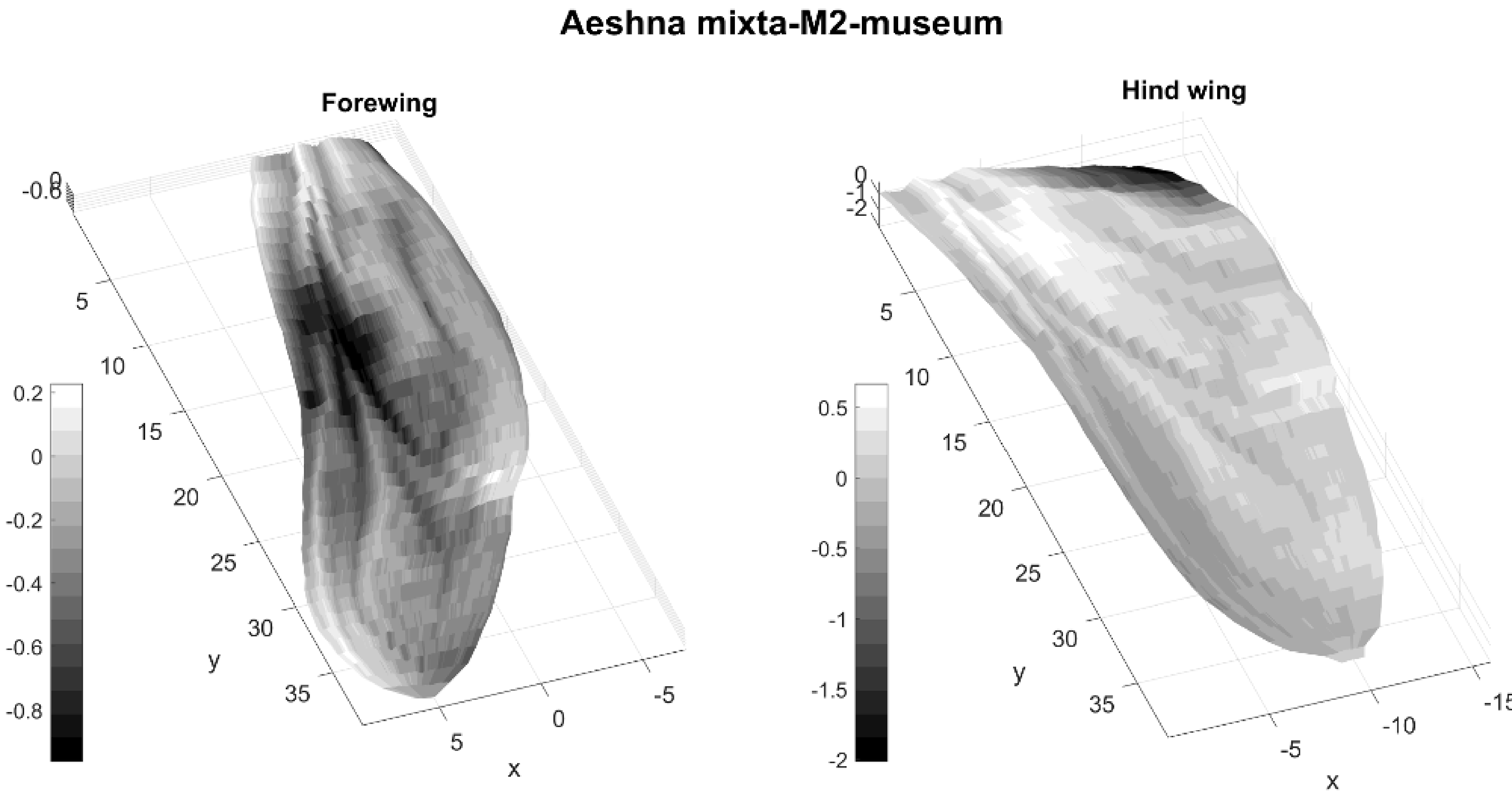

Aeshna mixta-M2-museum
Forewing
Hind wing
x
y

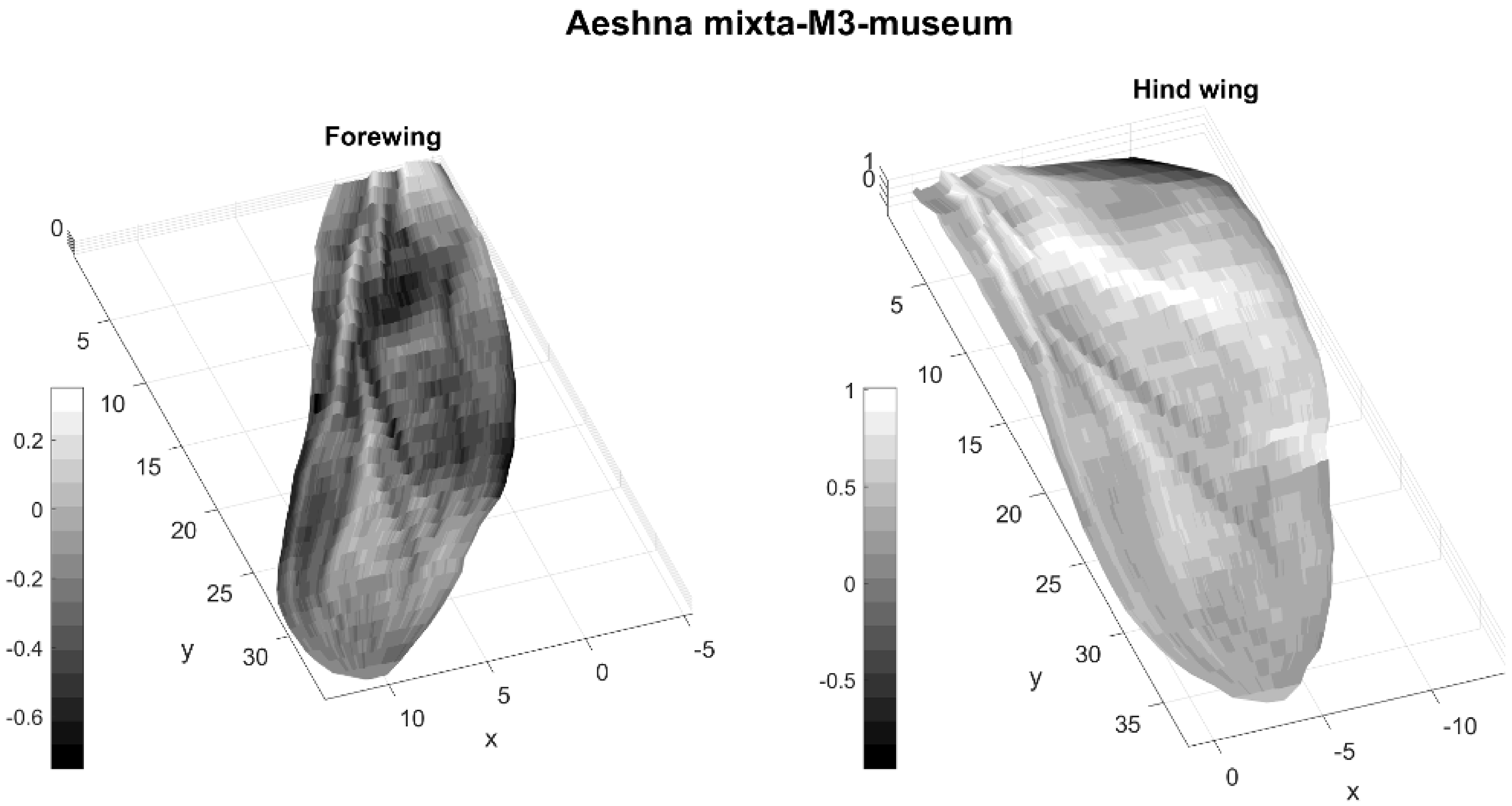

Aeshna mixta-M3-museum
Forewing
Hind wing
x
y

# Anax speratus-F1-museum

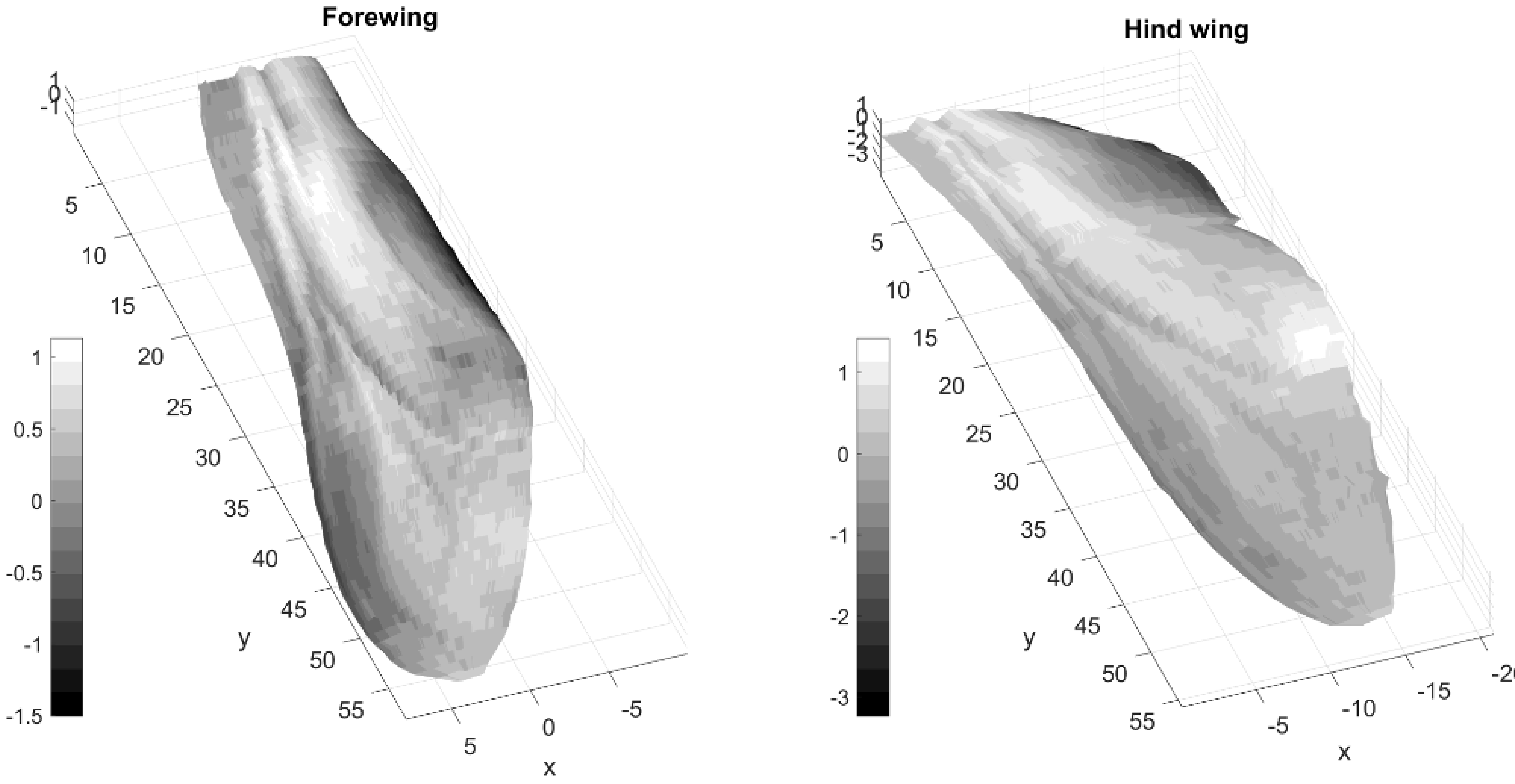

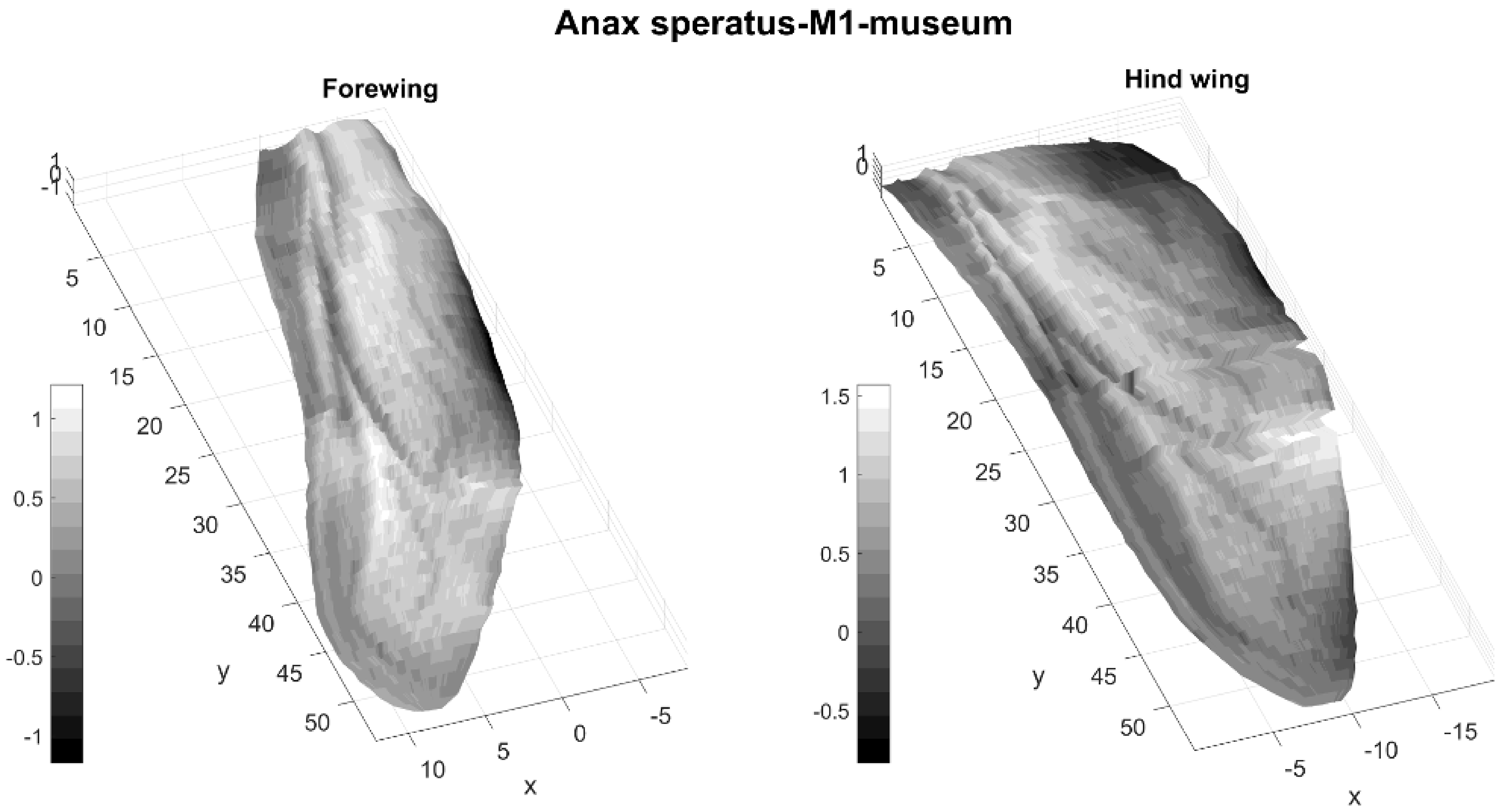

Anax speratus-M1-museum
Forewing
Hind wing
x
y

# Cordulia aenea-F1-museum

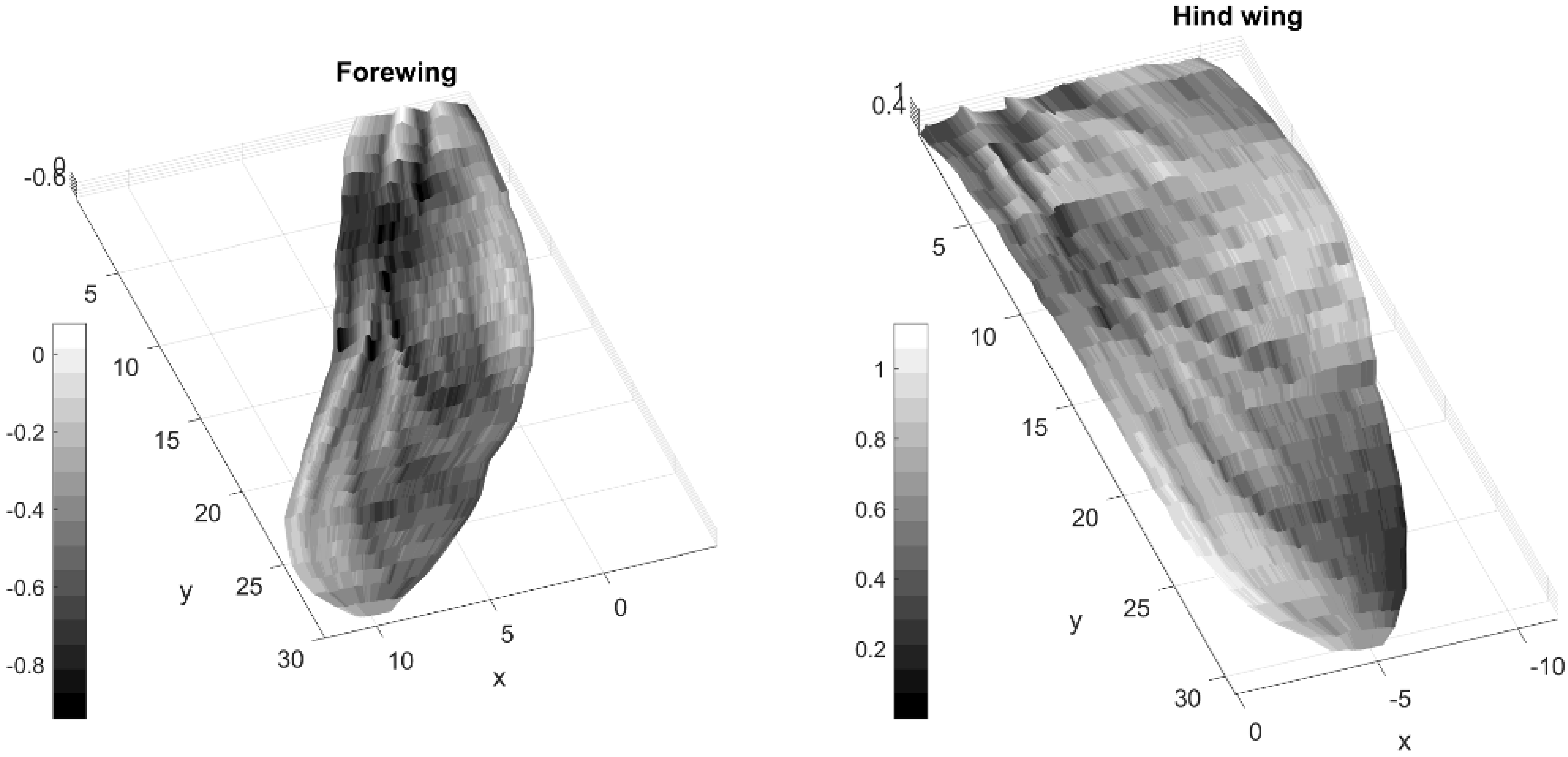

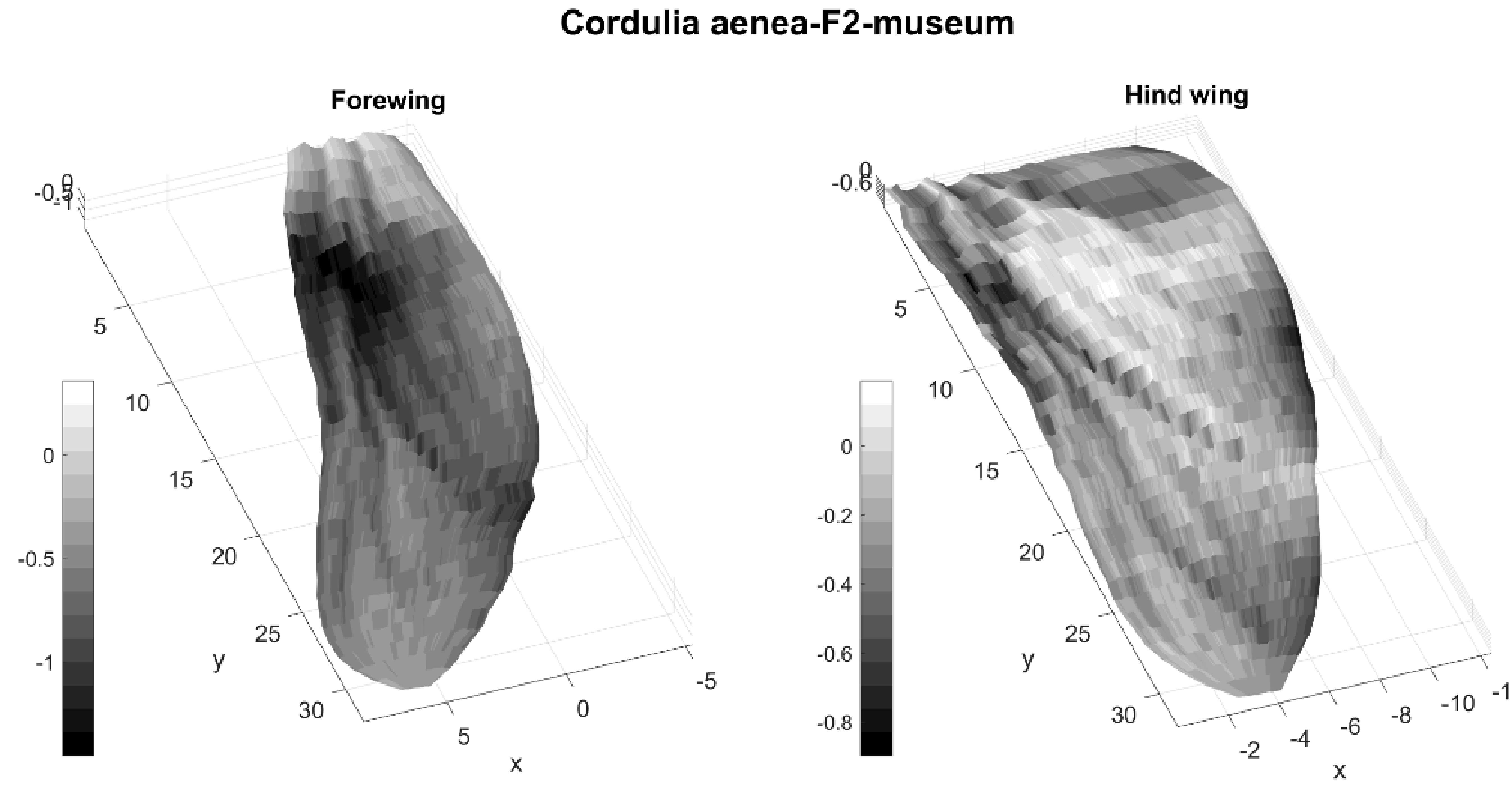
Cordulia aenea-F2-museum
Forewing
Hind wing
x
y

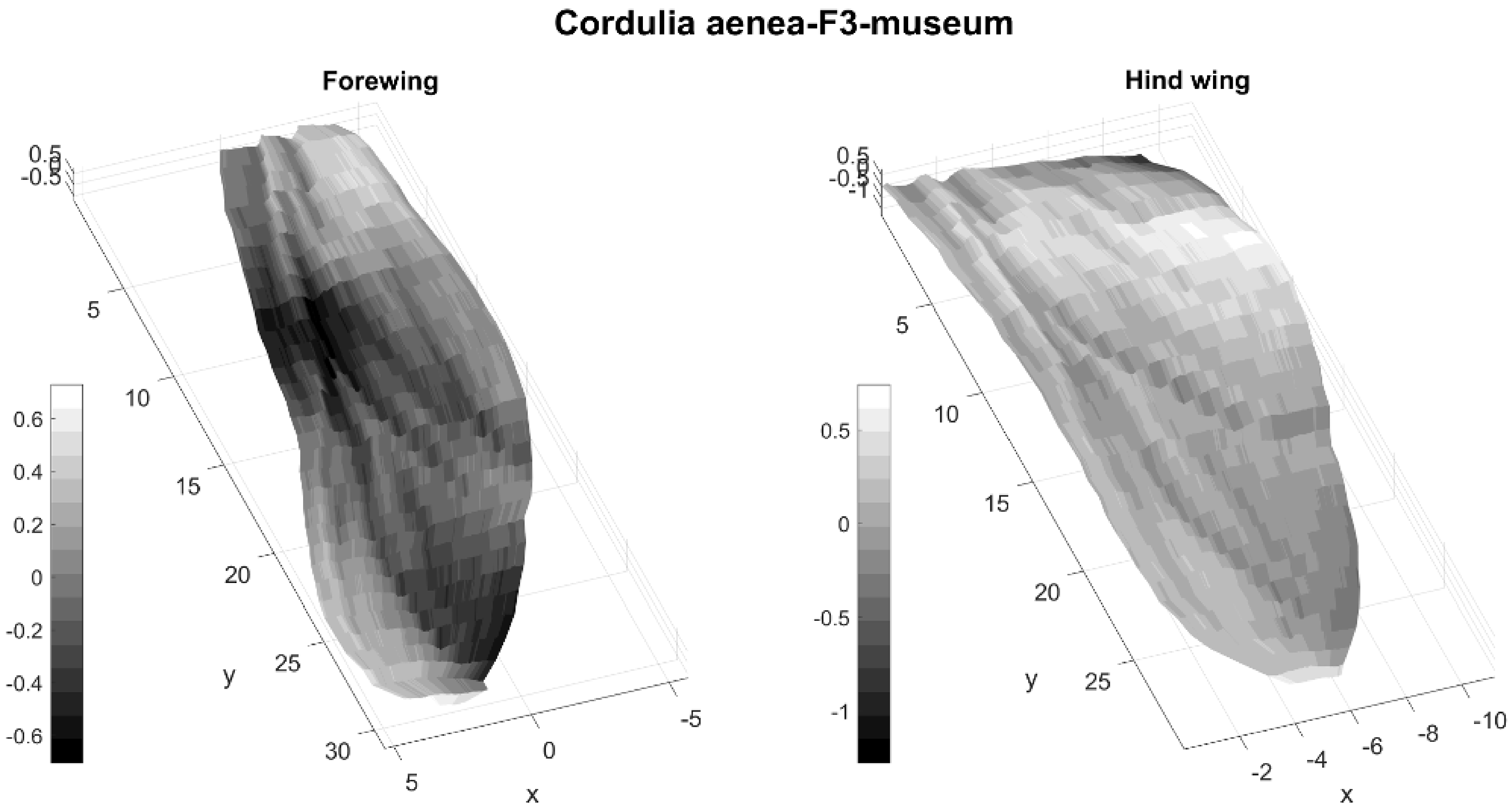
Cordulia aenea-F3-museum
Forewing
Hind wing
x
y

# Cordulia aenea-M1-museum

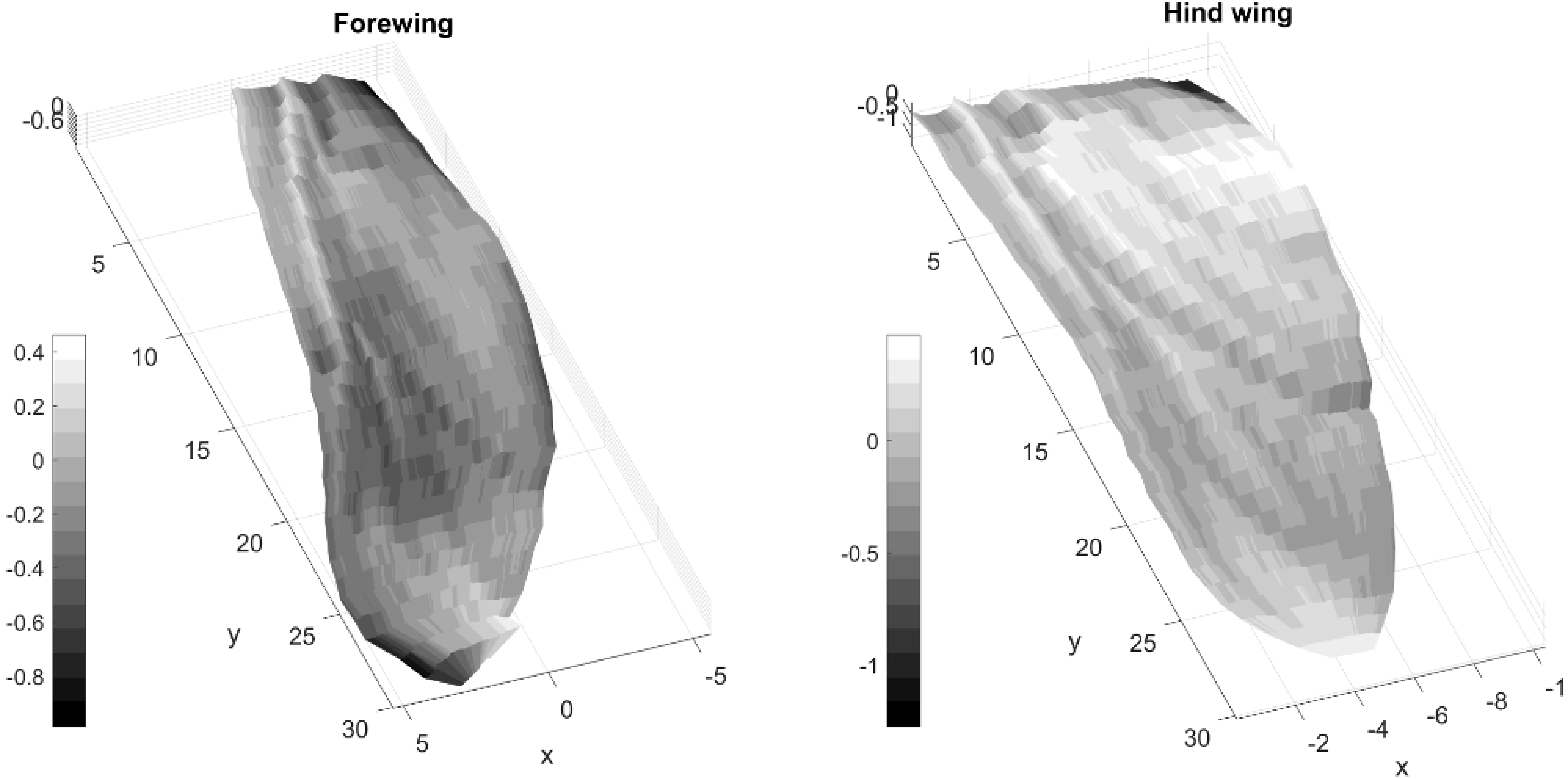

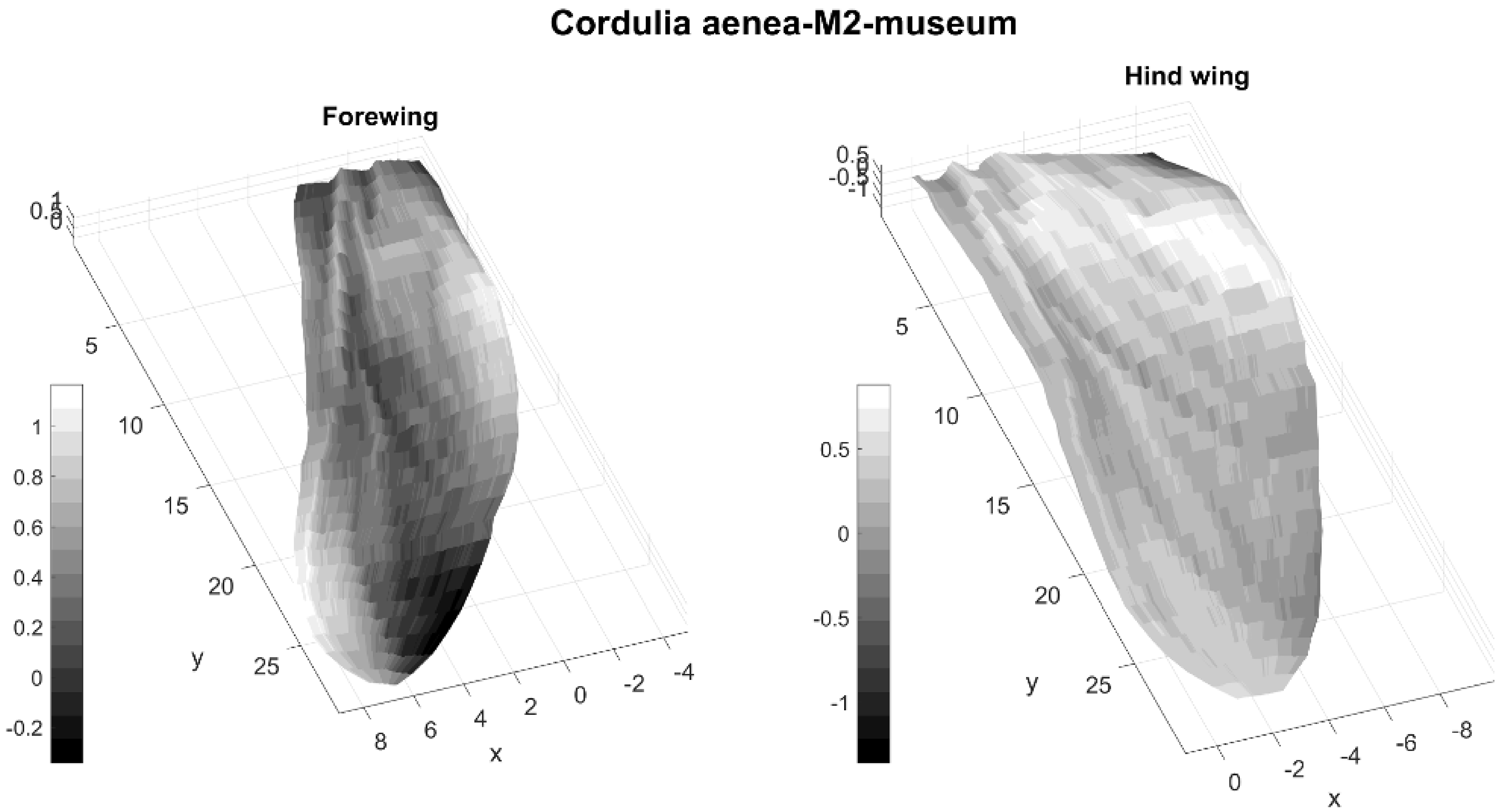
Cordulia aenea-M2-museum
Forewing
1
0.5
0
5
10
15
20
25
y
8
6
4
2
0
-2
-4
x
1
0.8
0.6
0.4
0.2
0
-0.2
Hind wing
0.5
0
-0.5
-1
5
10
15
20
25
y
0
-2
-4
-6
-8
x
0.5
0
-0.5
-1

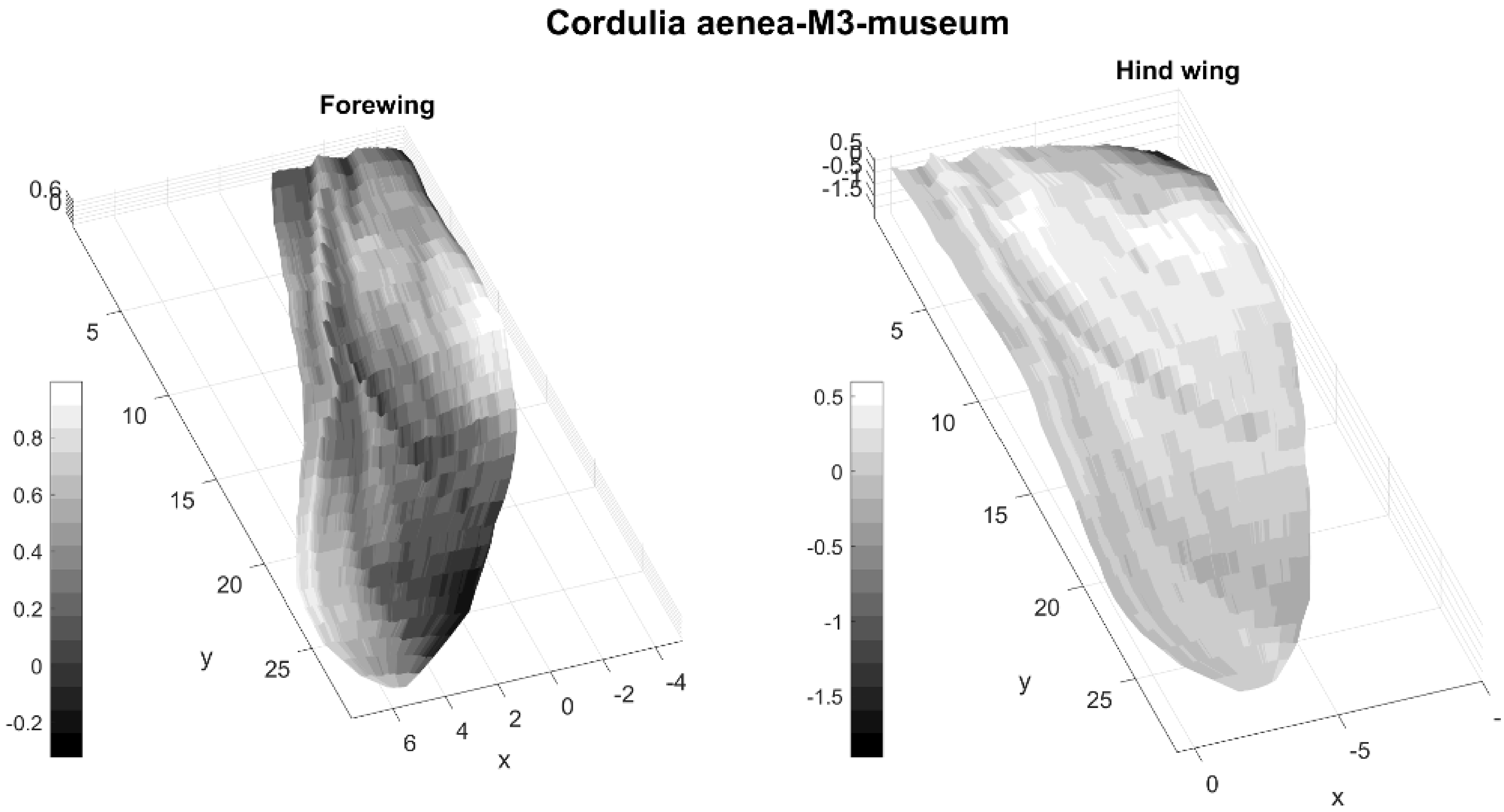

Cordulia aenea-M3-museum
Forewing
Hind wing
x
y

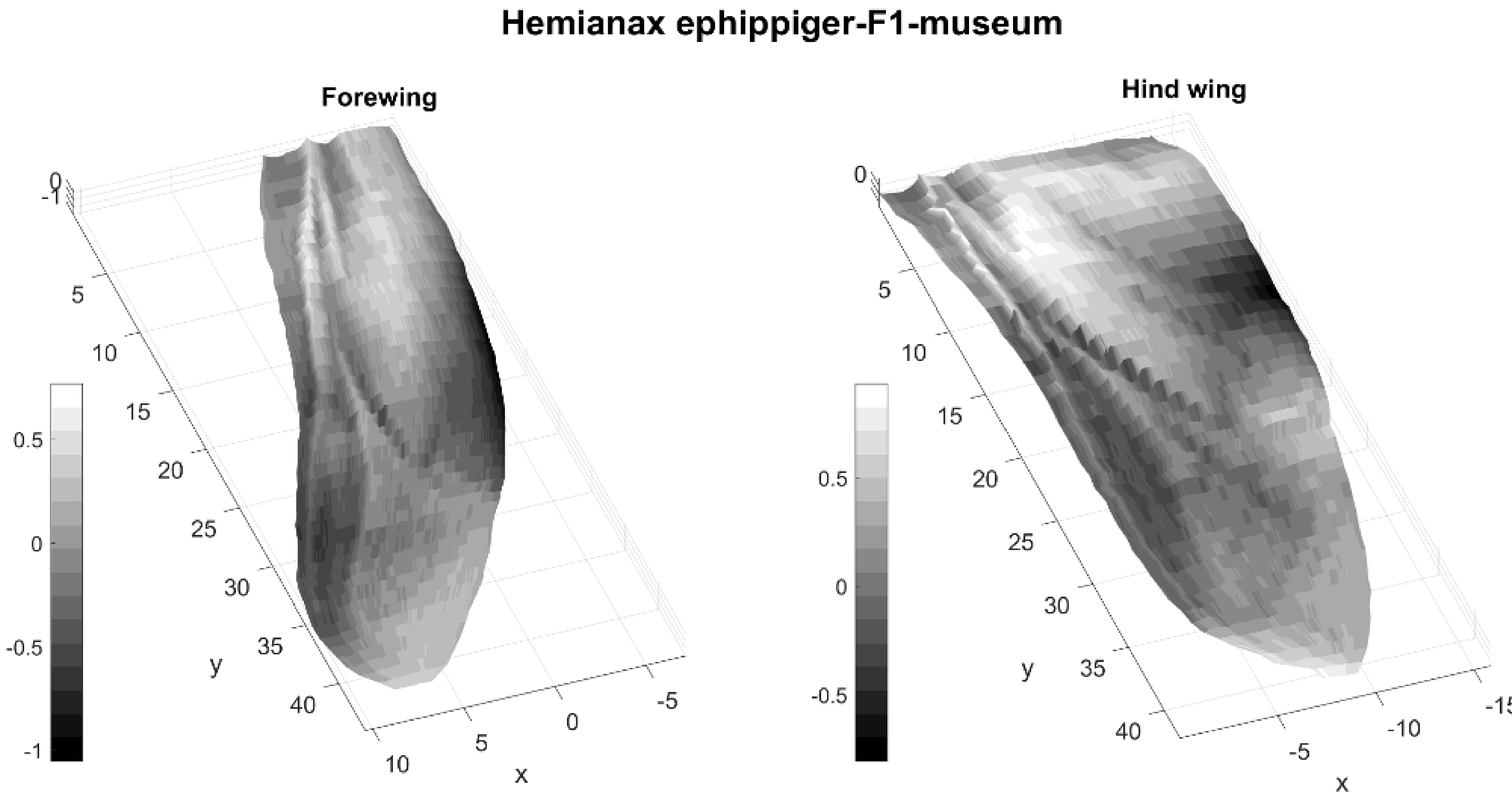

Hemianax ephippiger-F1-museum
Forewing
Hind wing
0
-1
5
10
15
20
25
30
35
40
y
10
5
0
-5
x
0.5
0
-0.5
-1
0
5
10
15
20
25
30
35
40
y
-5
-10
-15
x
0.5
0
-0.5

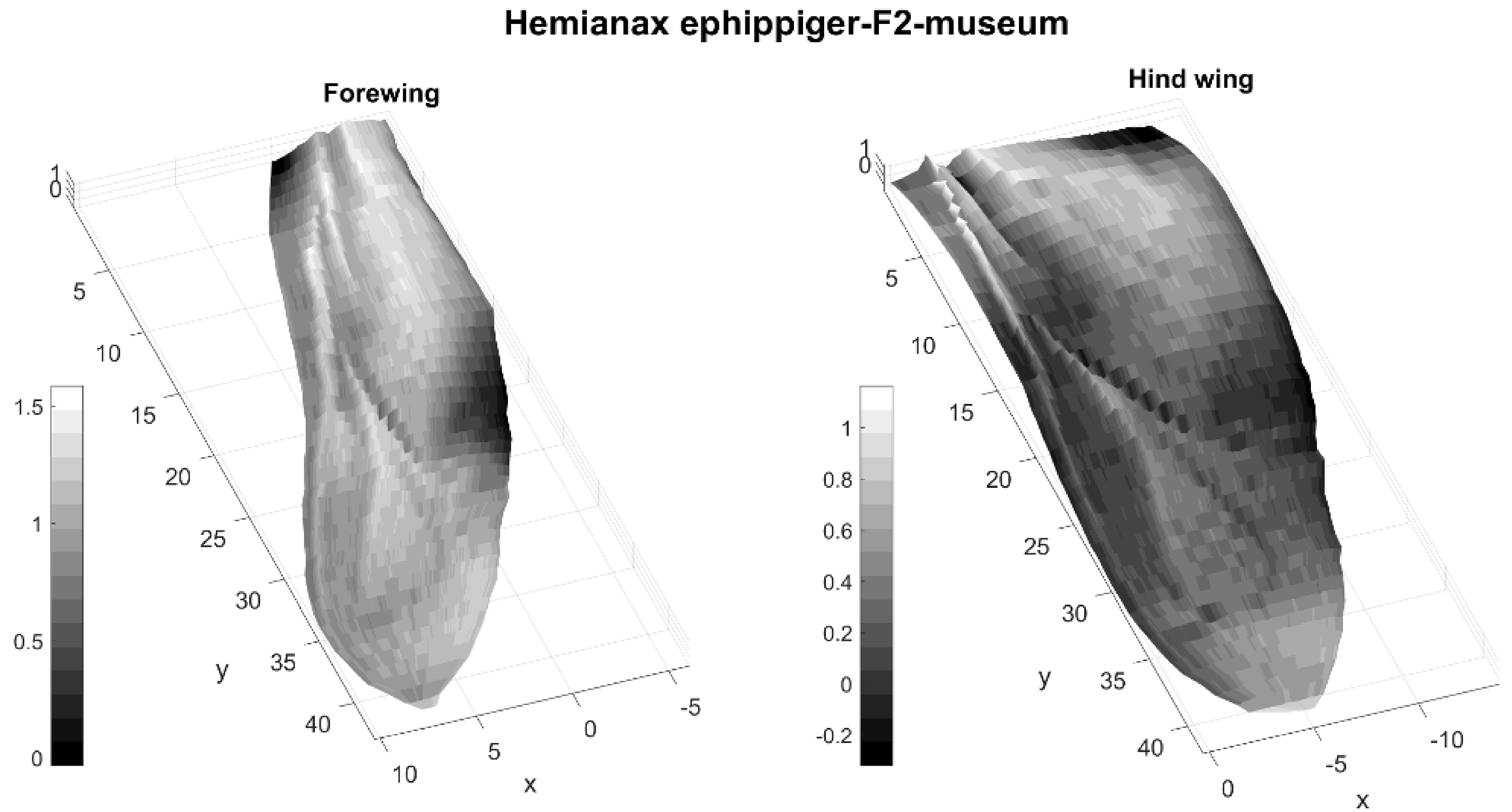
Hemianax ephippiger-F2-museum
Forewing
Hind wing
1
0
5
10
15
20
25
30
35
40
y
10
5
0
-5
x
1.5
1
0.5
0
1
0
5
10
15
20
25
30
35
40
y
0
-5
-10
x
1
0.8
0.6
0.4
0.2
0
-0.2

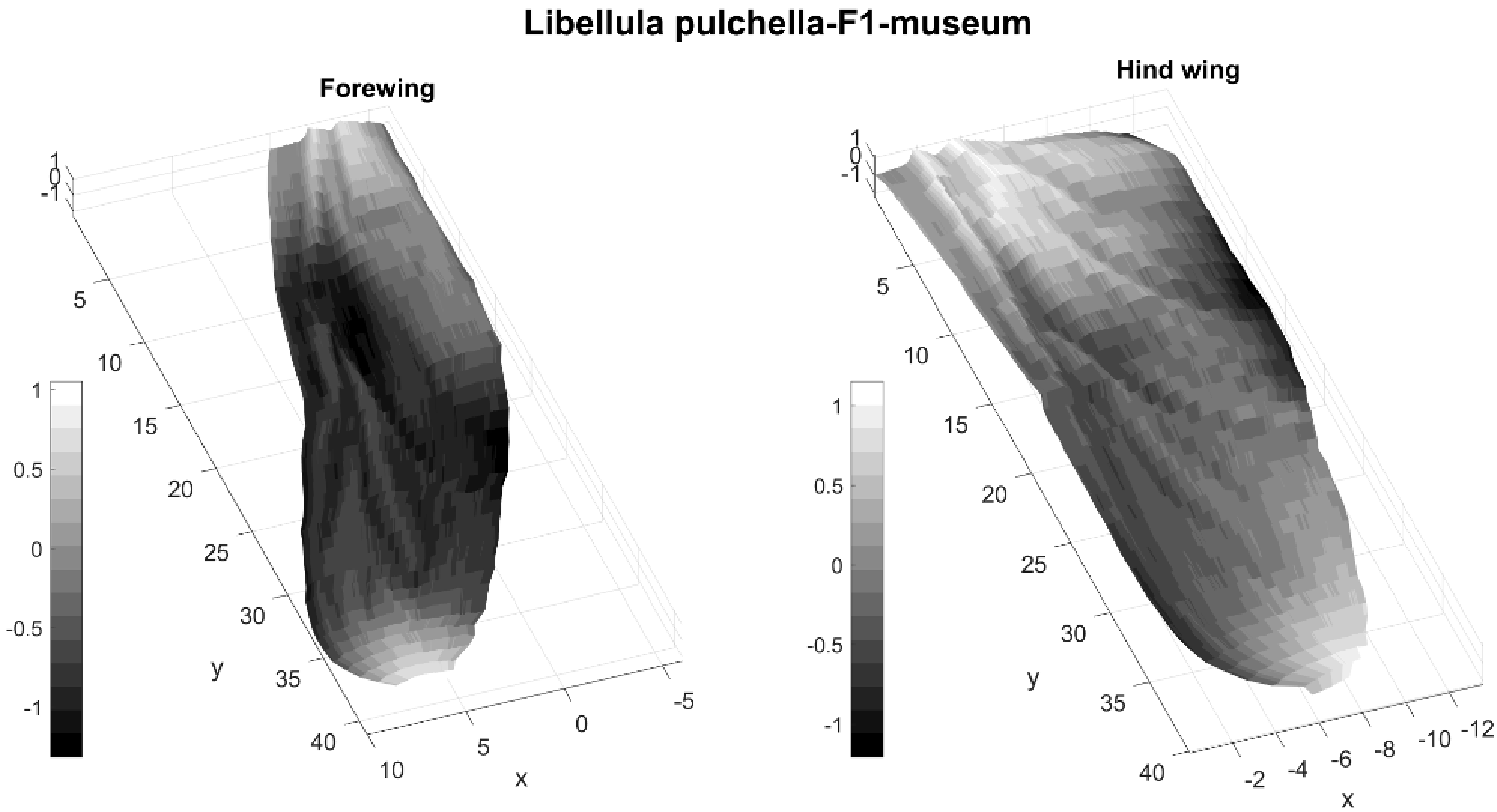
Libellula pulchella-F1-museum
Forewing
Hind wing
x
y
5
10
15
20
25
30
35
40
10
0
-5
1
0
-1
0.5
-0.5
-2
-4
-6
-8
-10
-12

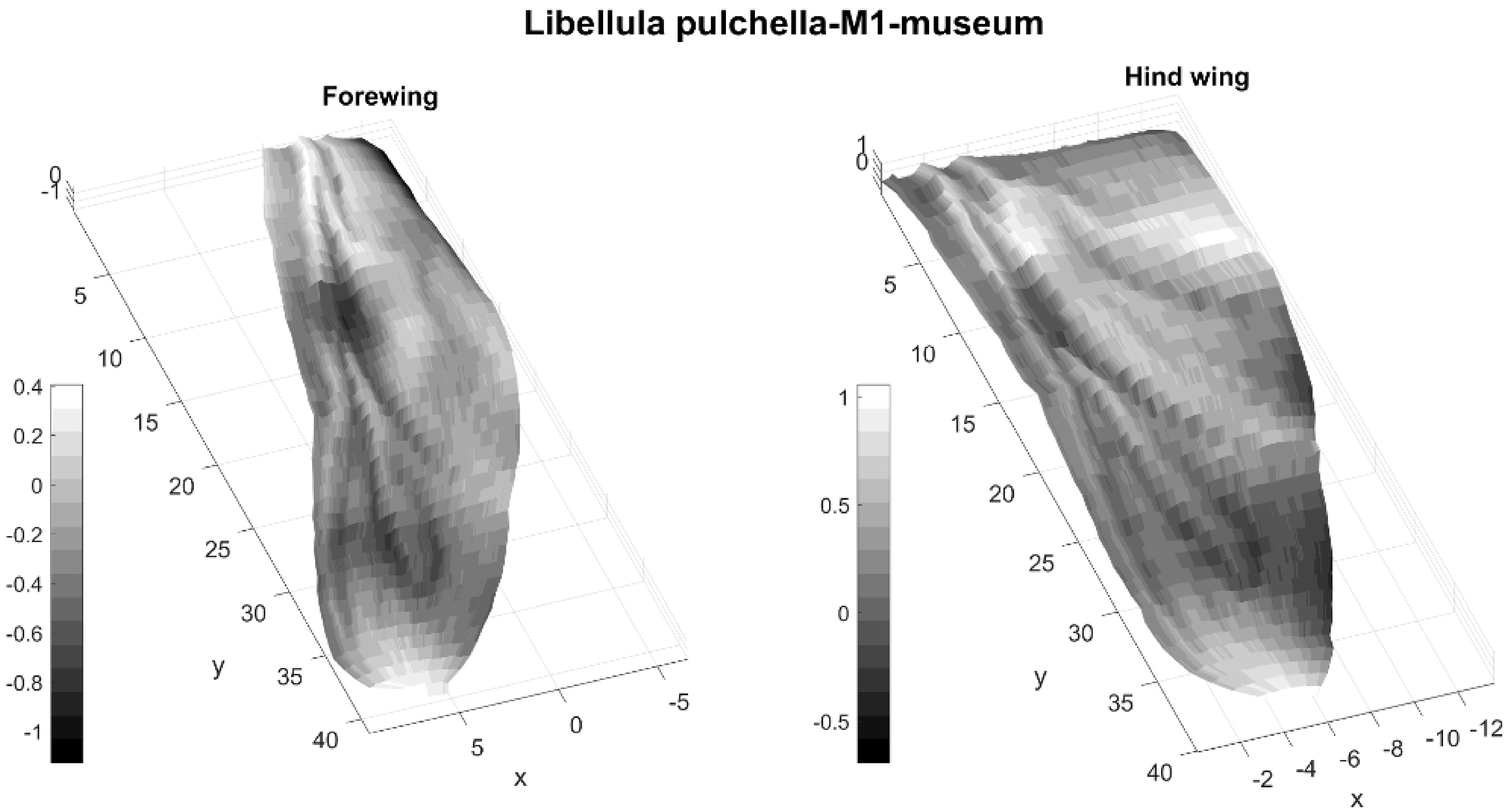

Libellula pulchella-M1-museum
Forewing
Hind wing
y
x

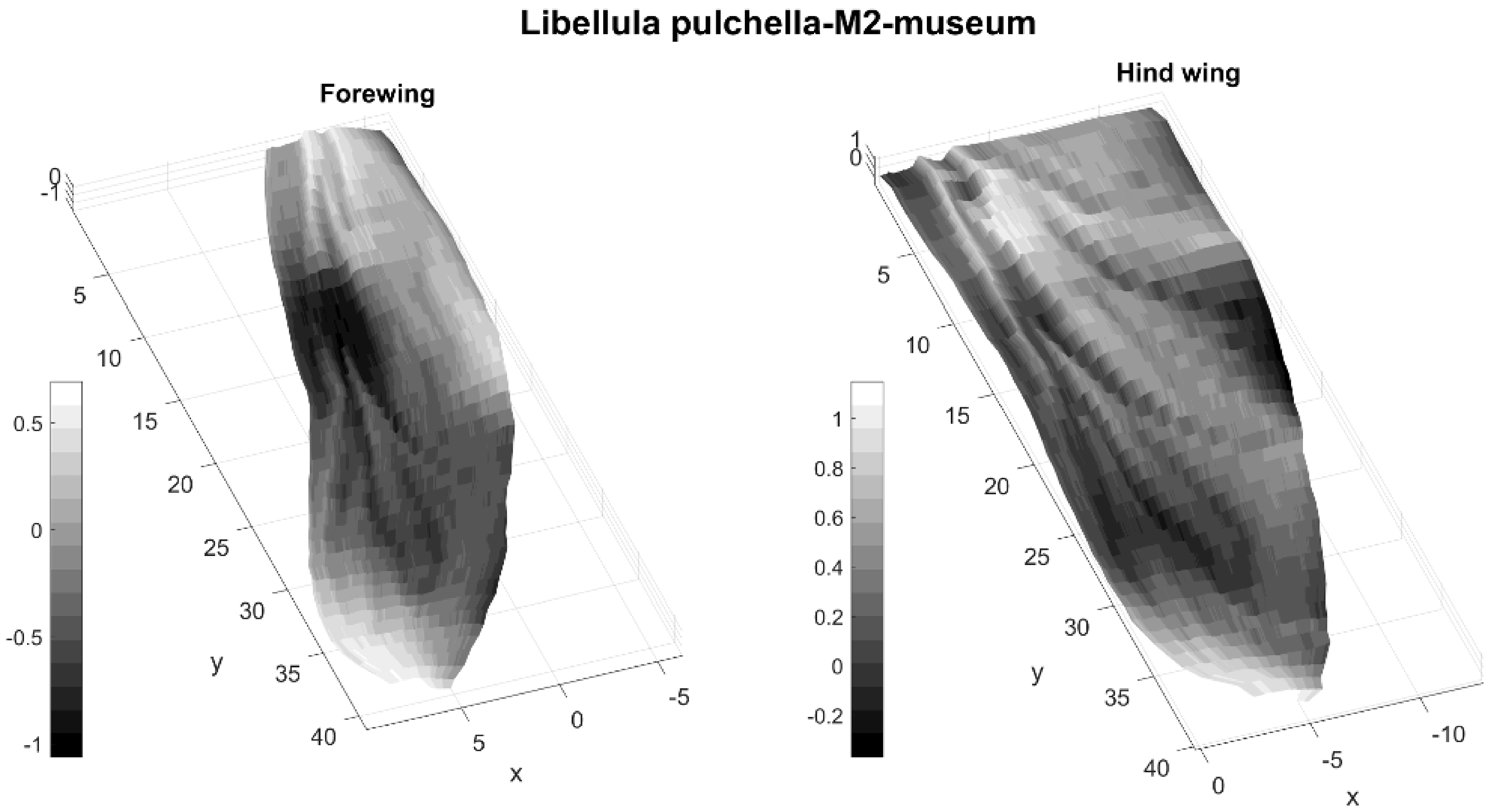

Libellula pulchella-M2-museum
Forewing
Hind wing
x
y

# Libellula variegata-F1-museum

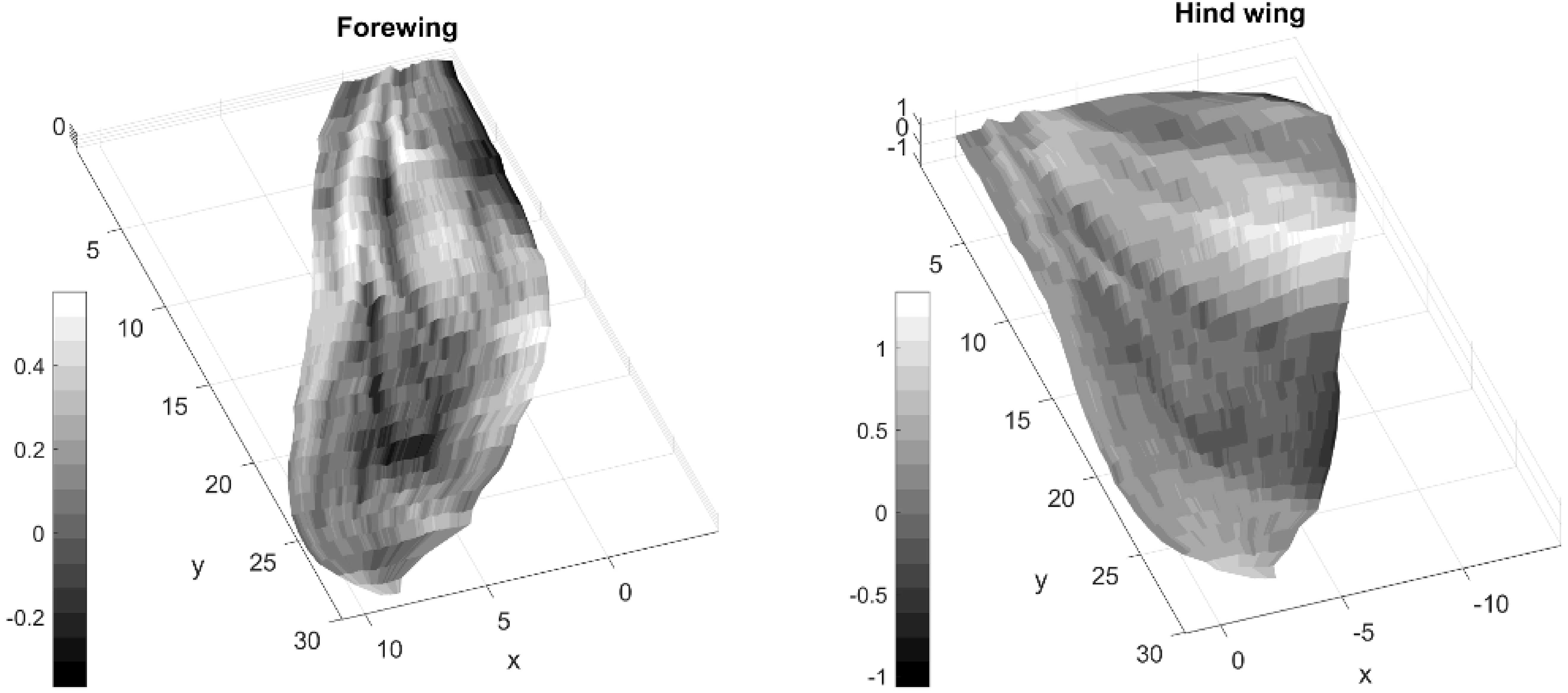

# Orthetrum cancellatum-F1-museum

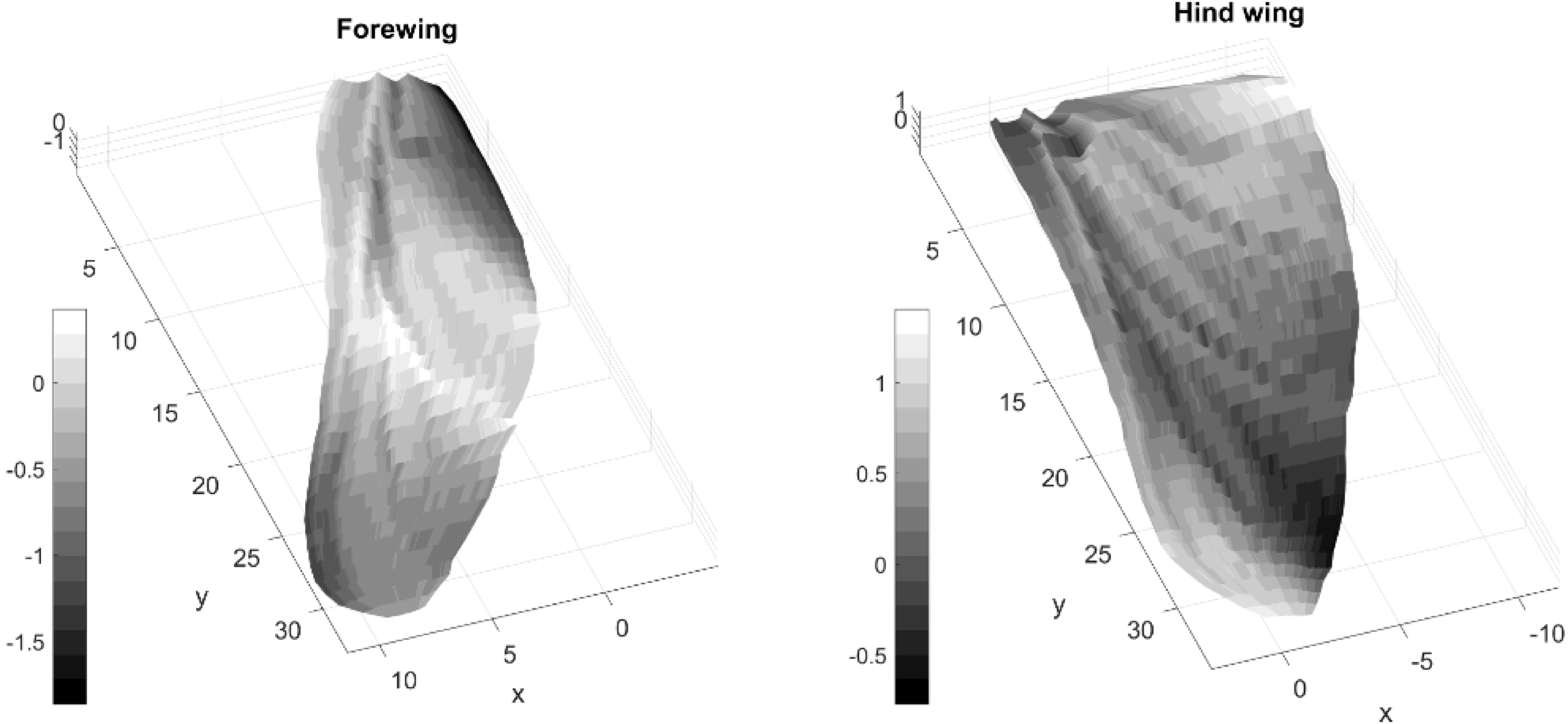

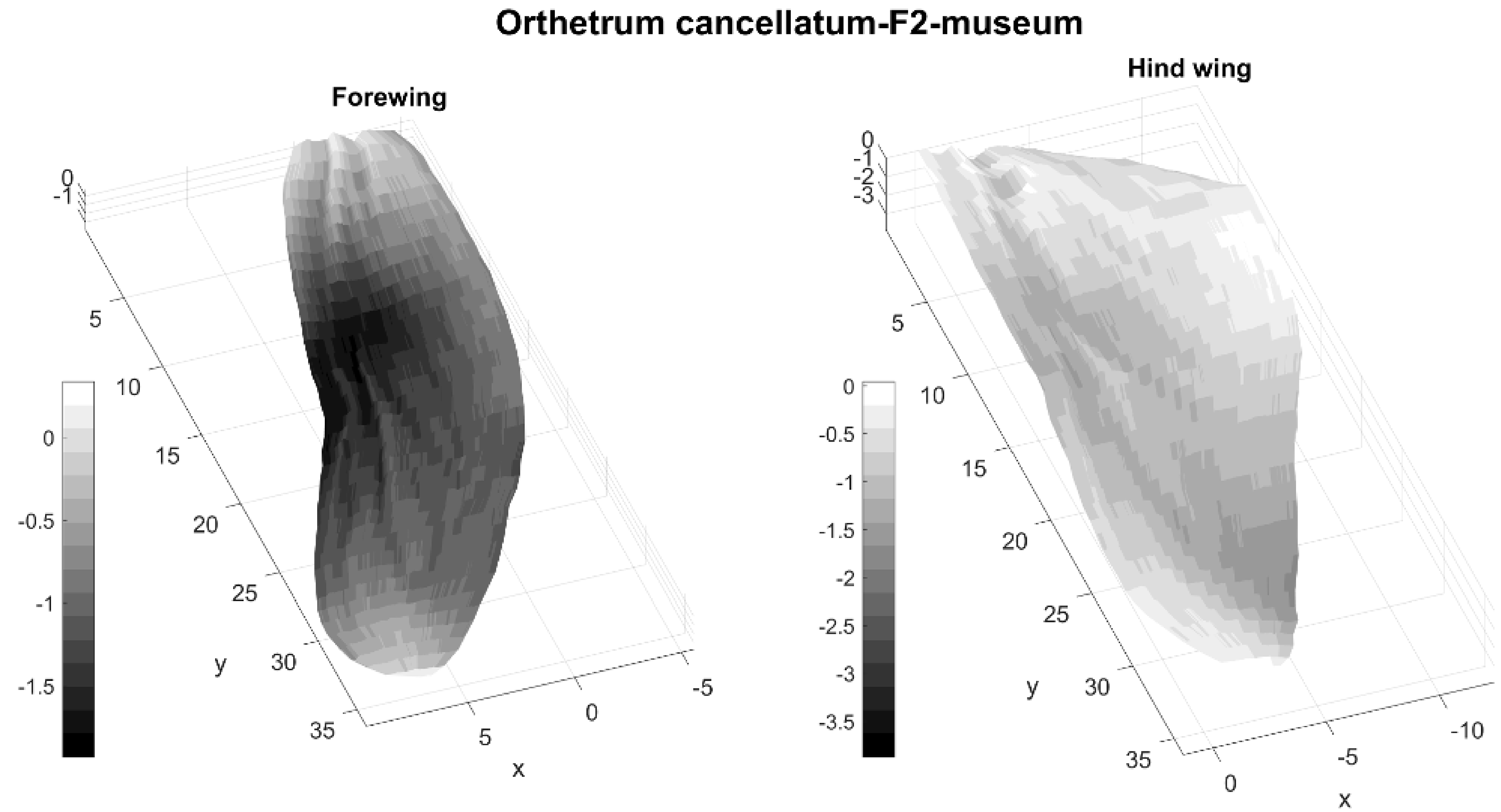
Orthetrum cancellatum-F2-museum
Forewing
Hind wing
0
-1
5
10
15
20
25
30
35
y
x
0
-0.5
-1
-1.5
-5
0
5
-1
-2
-3
-2.5
-3.5
-10

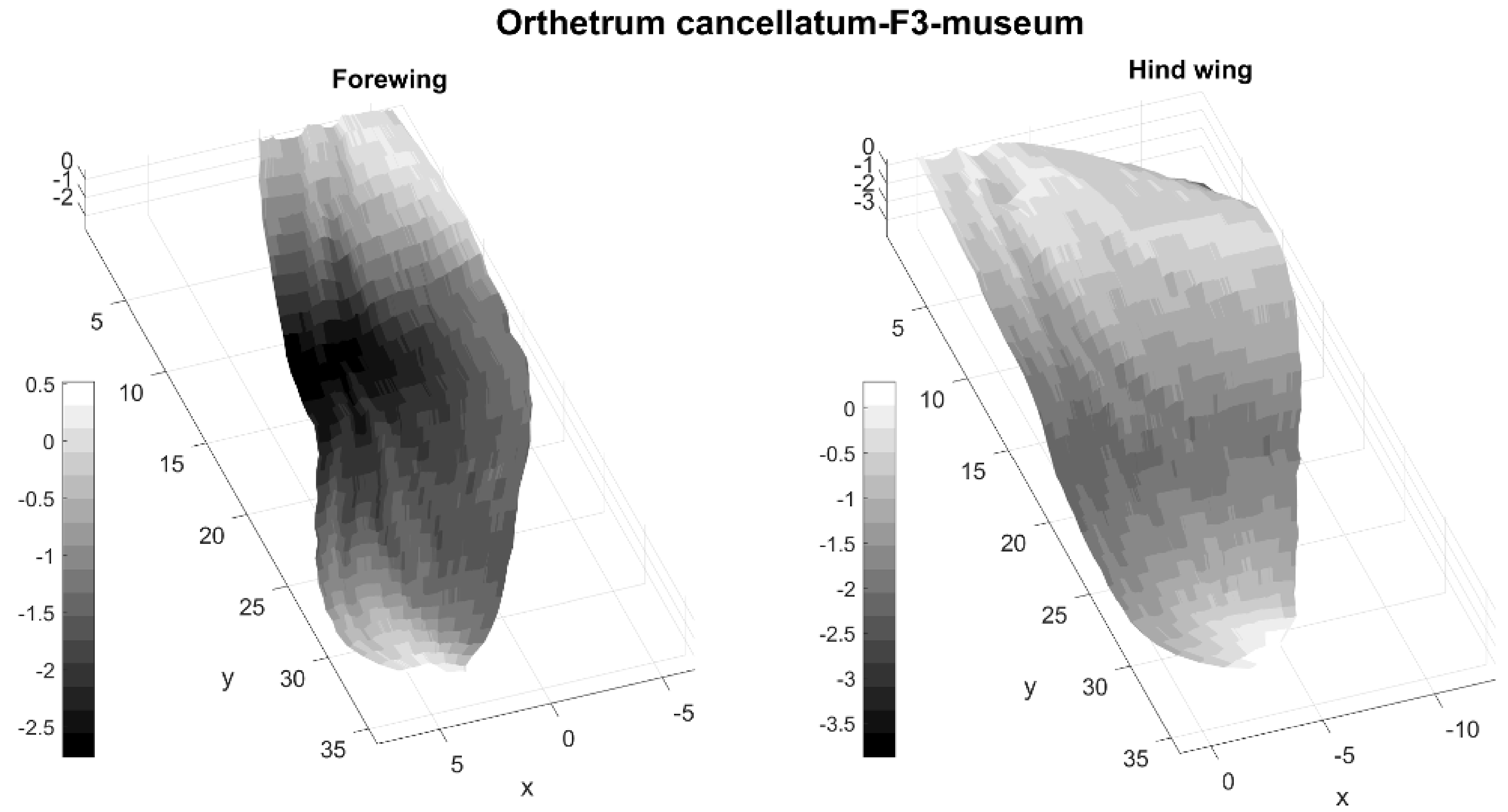
Orthetrum cancellatum-F3-museum
Forewing
Hind wing
0
-1
-2
-3
0.5
0
-0.5
-1
-1.5
-2
-2.5
-3
-3.5
5
10
15
20
25
30
35
y
x
-5
-10

# Pantala flavescens-F1-museum

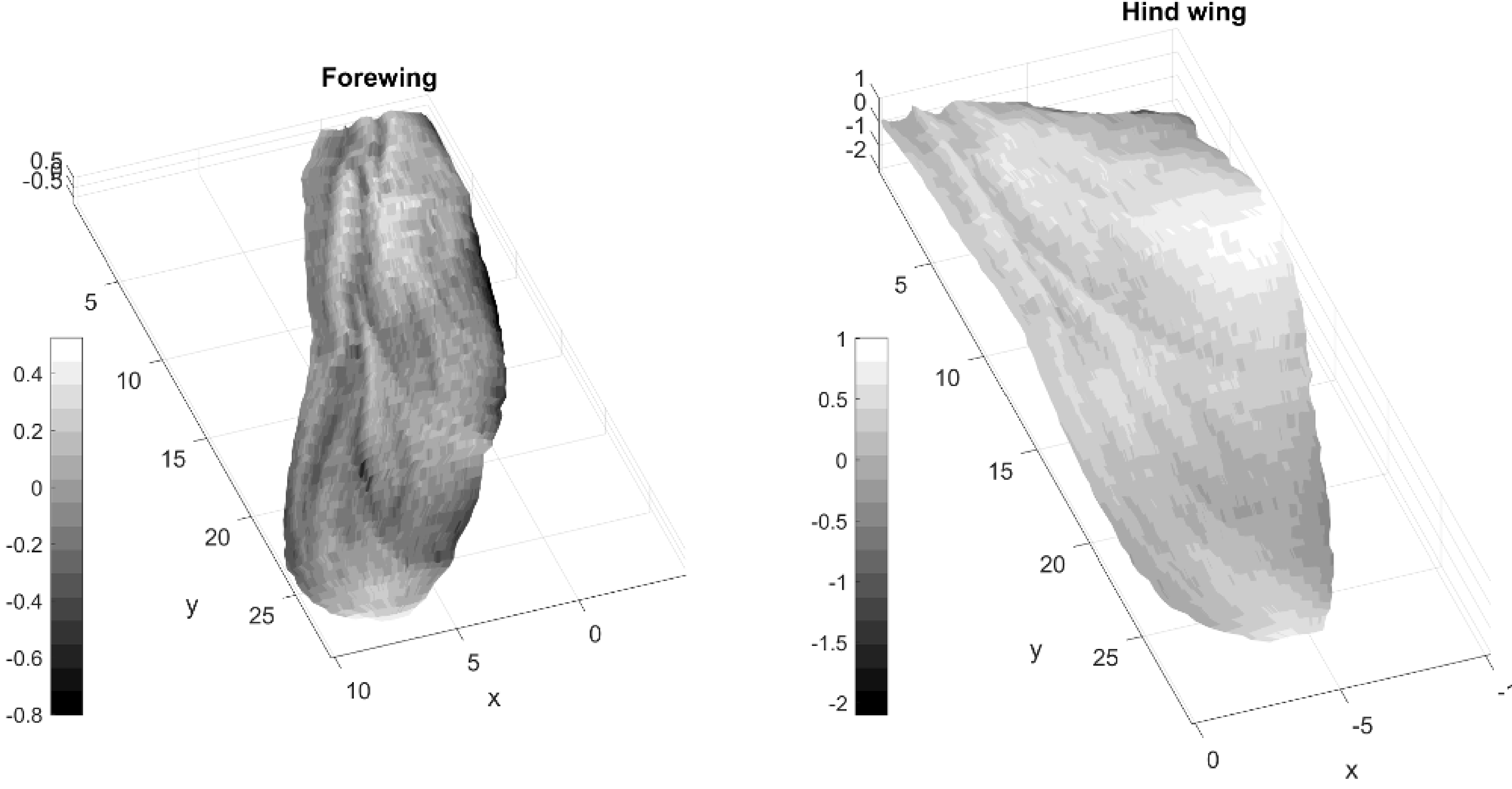

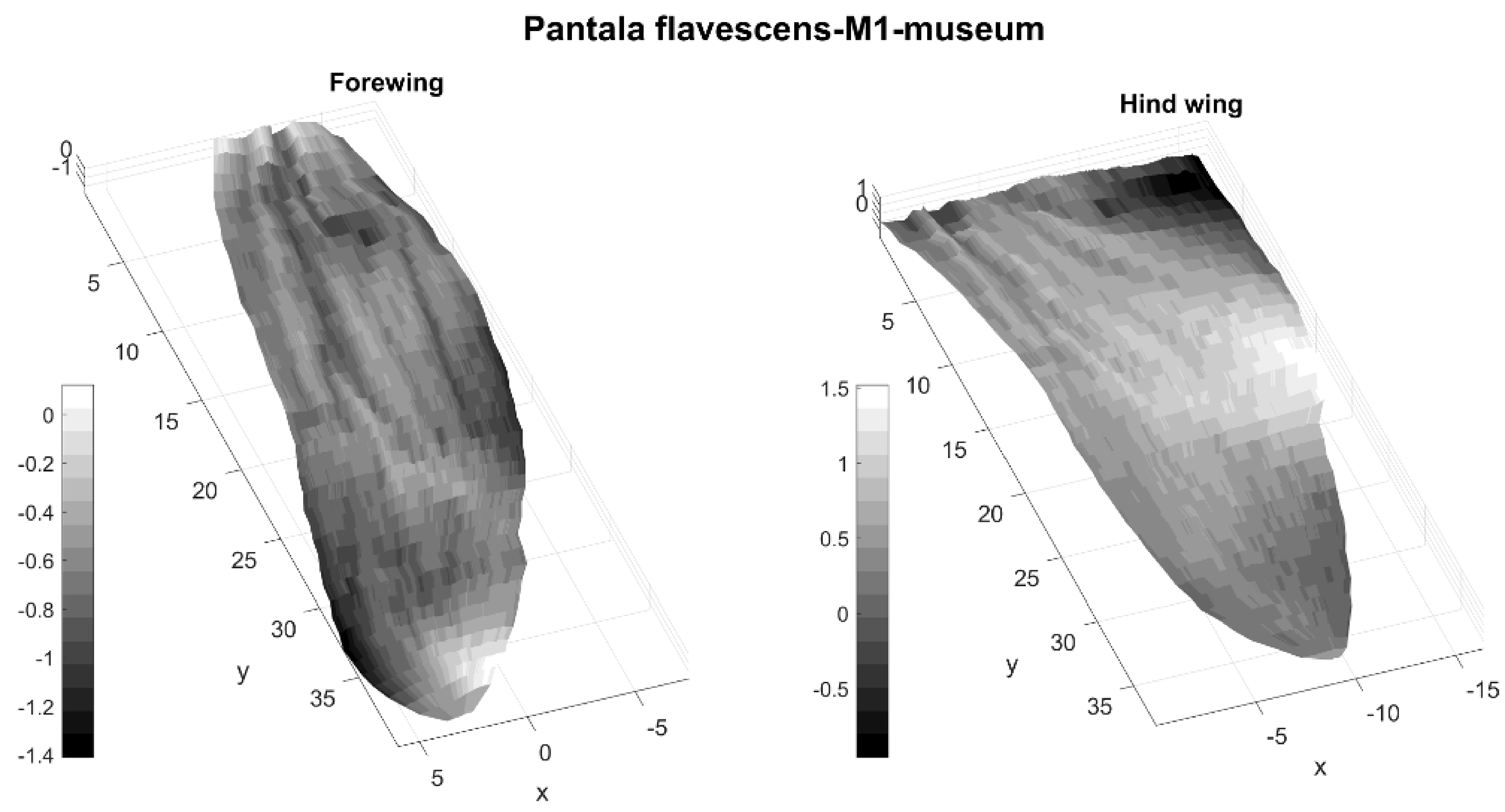

Pantala flavescens-M1-museum
Forewing
Hind wing
0
-1
5
10
15
20
25
30
35
y
x
0
-0.2
-0.4
-0.6
-0.8
-1
-1.2
-1.4
5
0
-5
1
0
1.5
1
0.5
0
-0.5
-5
-10
-15

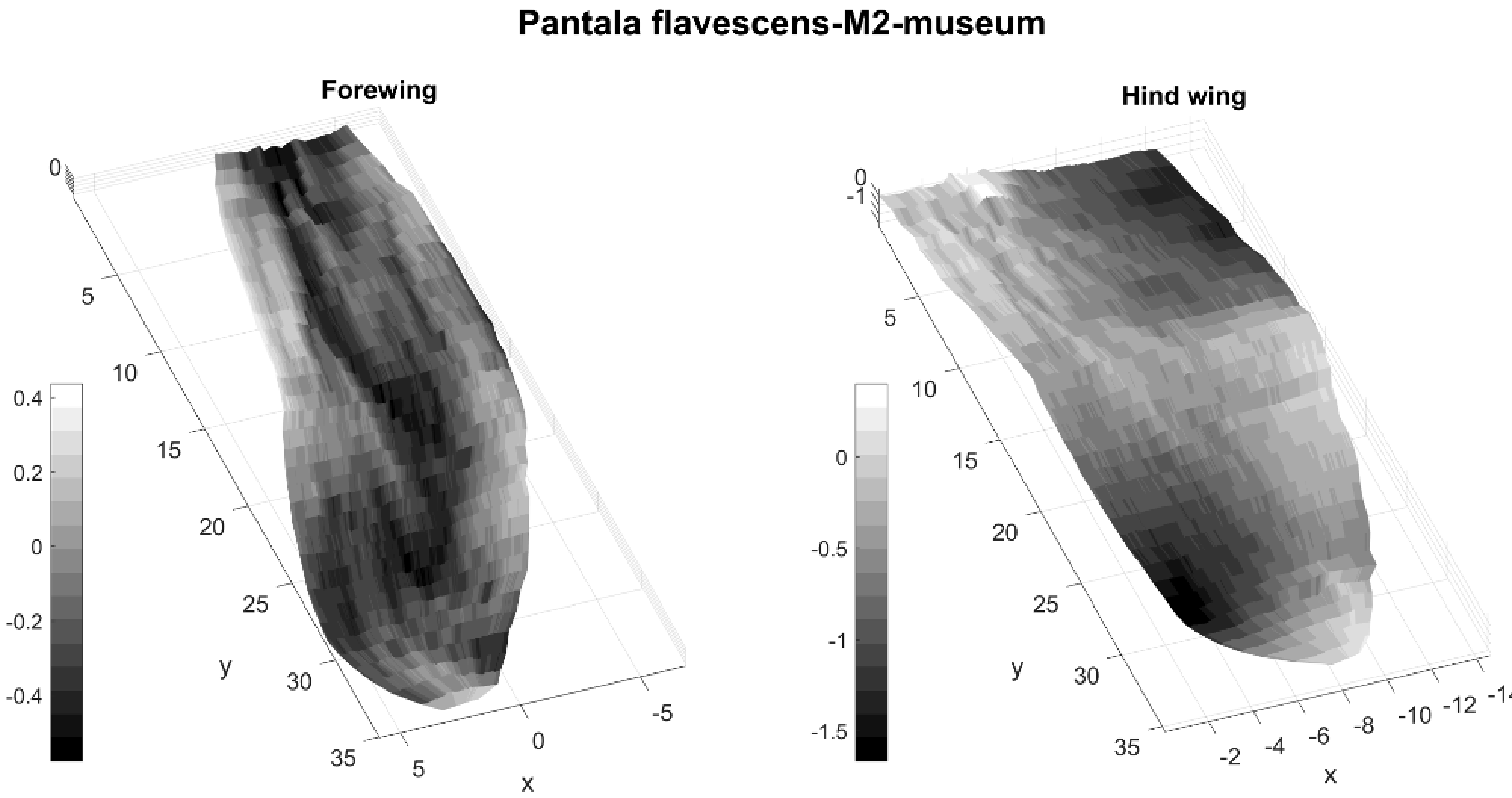

Pantala flavescens-M2-museum
Forewing
Hind wing
x
y

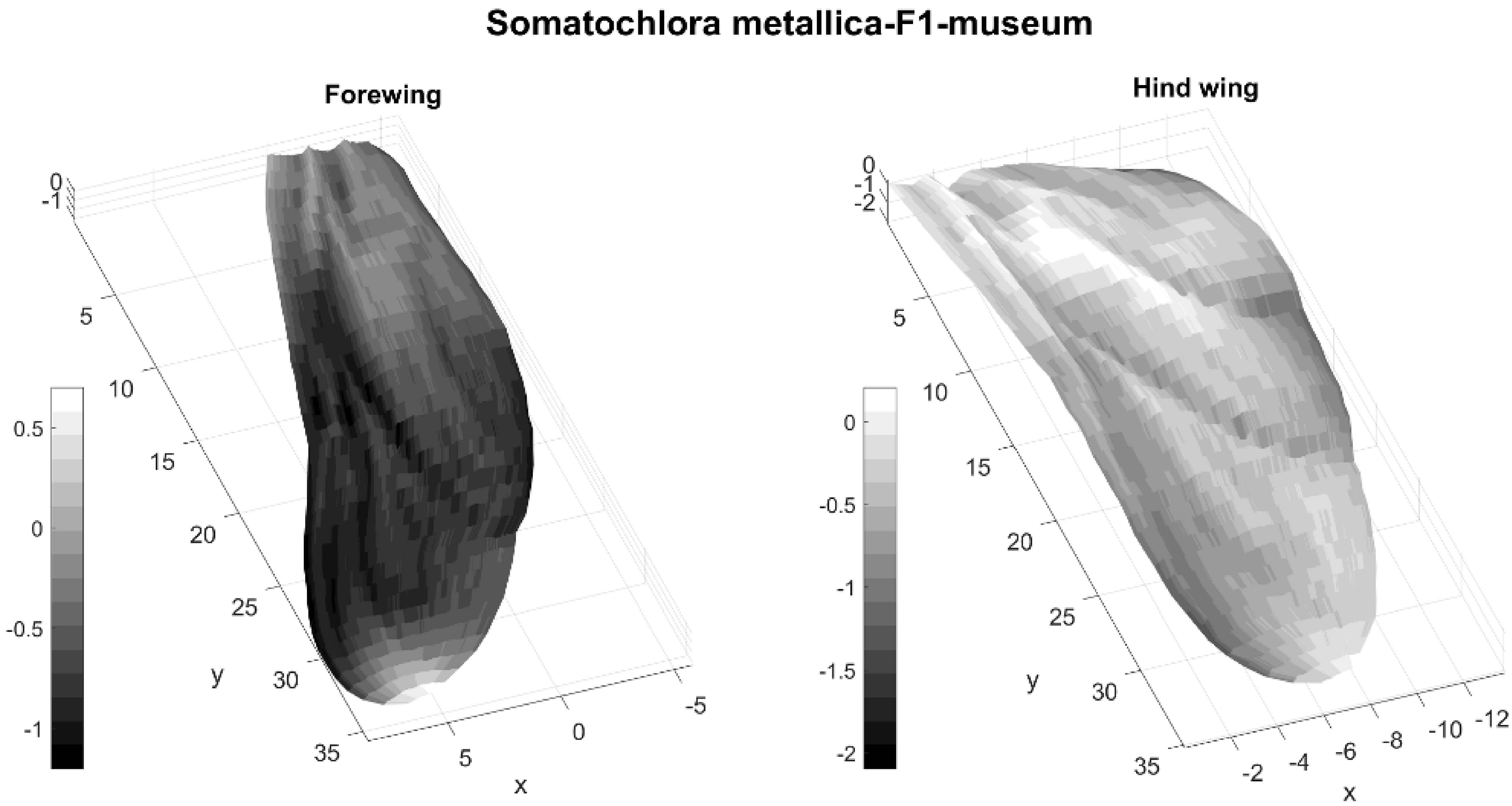
Somatochlora metallica-F1-museum
Forewing
Hind wing
y
x
0
-1
5
10
15
20
25
30
35
0.5
0
-0.5
-1
-5
0
5
-2
-1.5
-2
-4
-6
-8
-10
-12

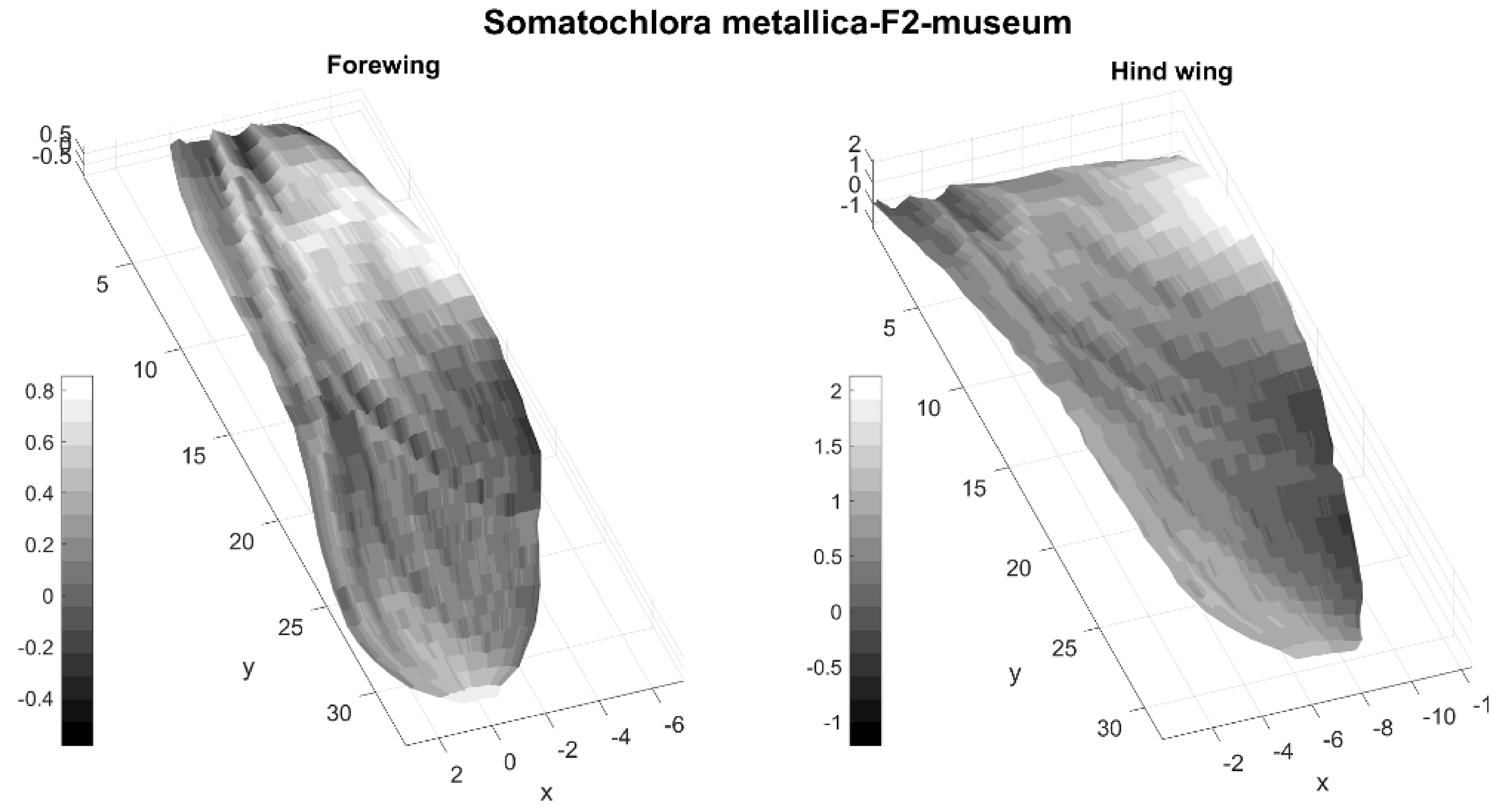

Somatochlora metallica-F2-museum
Forewing
Hind wing
y
x

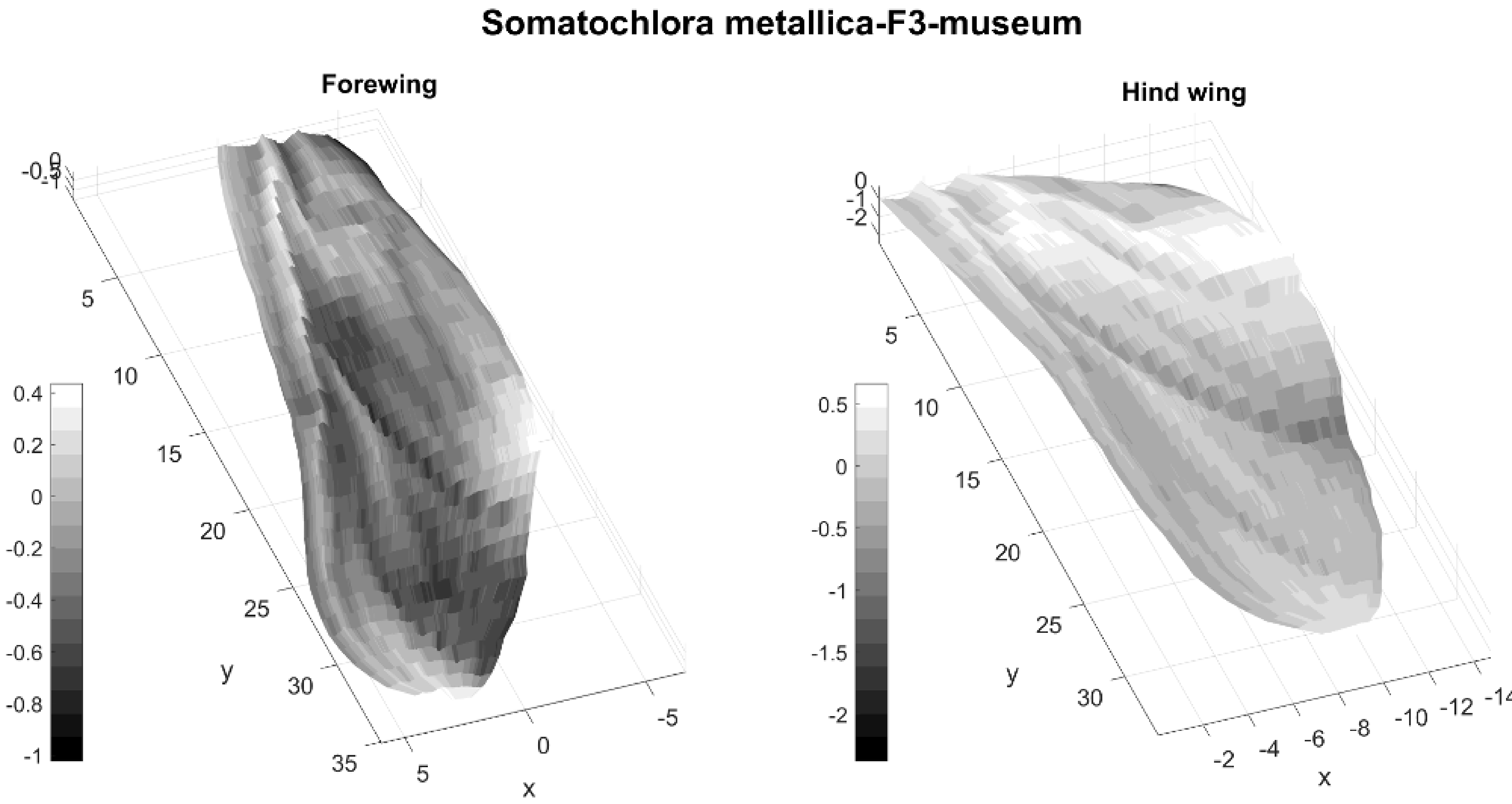

Somatochlora metallica-F3-museum
Forewing
Hind wing
y
x

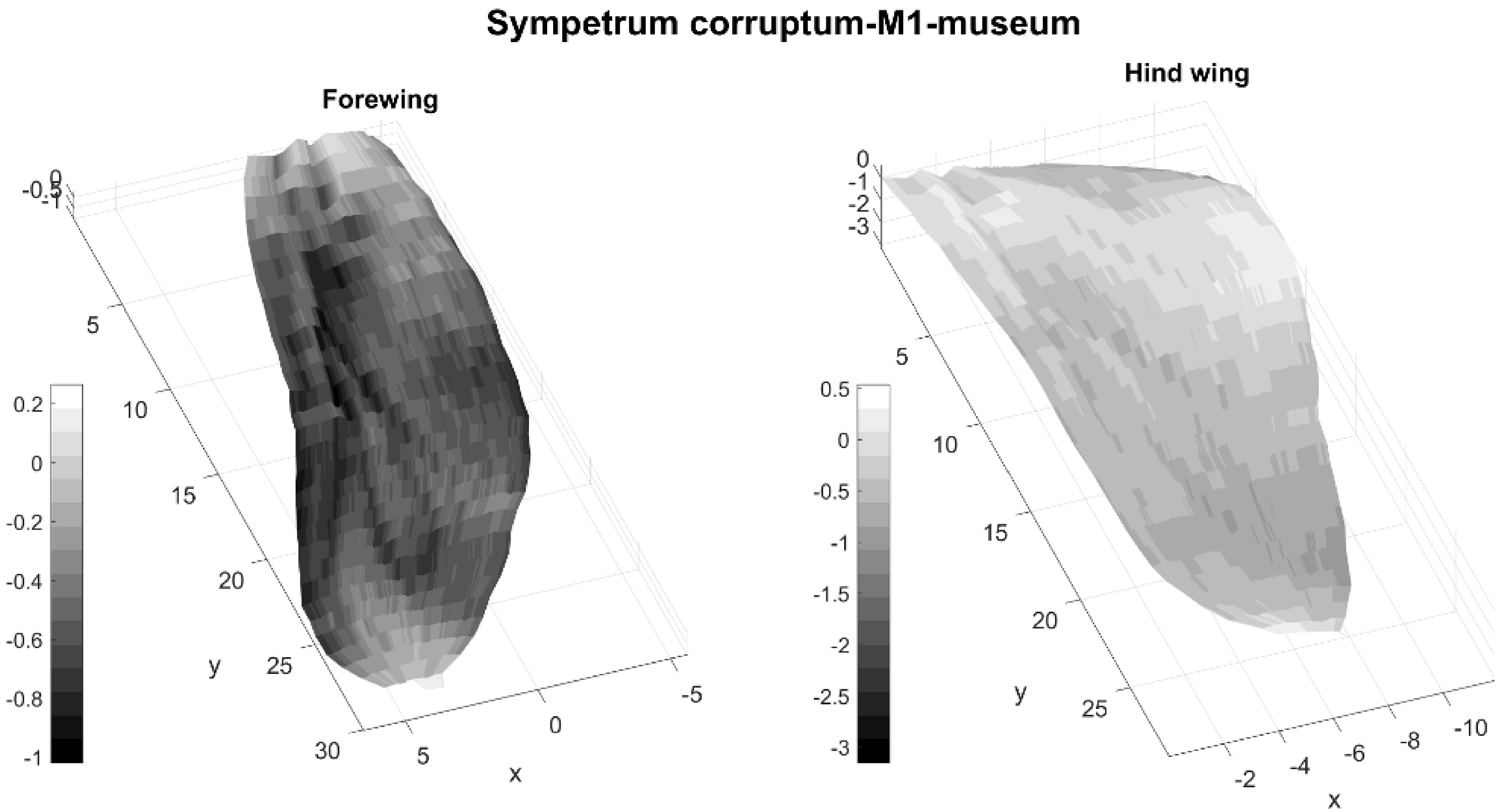
Sympetrum corruptum-M1-museum
Forewing
Hind wing
x
y
x
y

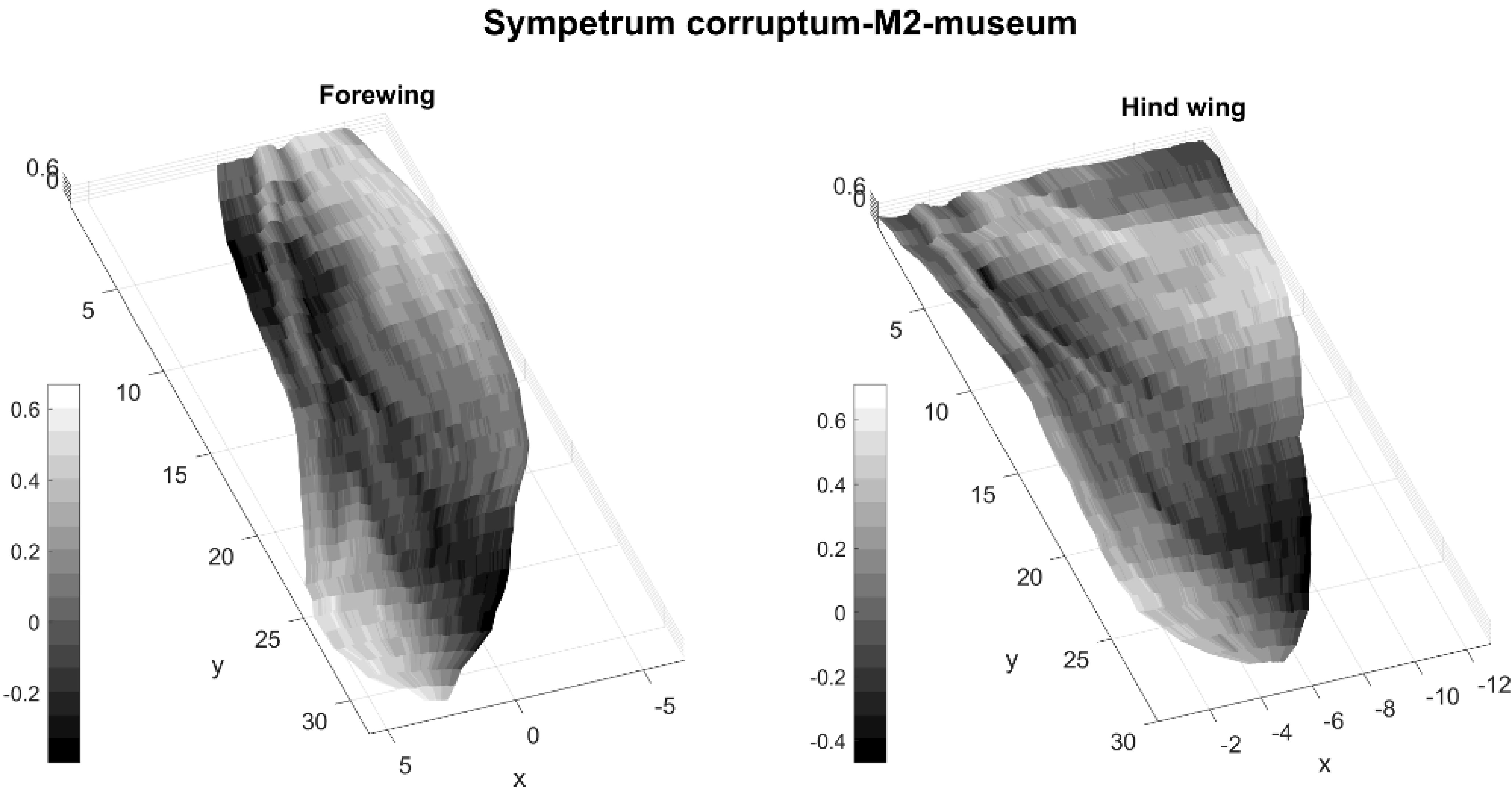

Sympetrum corruptum-M2-museum
Forewing
Hind wing
0.6
0
5
10
15
20
25
30
y
5
0
-5
x
0.6
0.4
0.2
0
-0.2
0.6
0
5
10
15
20
25
30
y
-2
-4
-6
-8
-10
-12
x
0.6
0.4
0.2
0
-0.2
-0.4

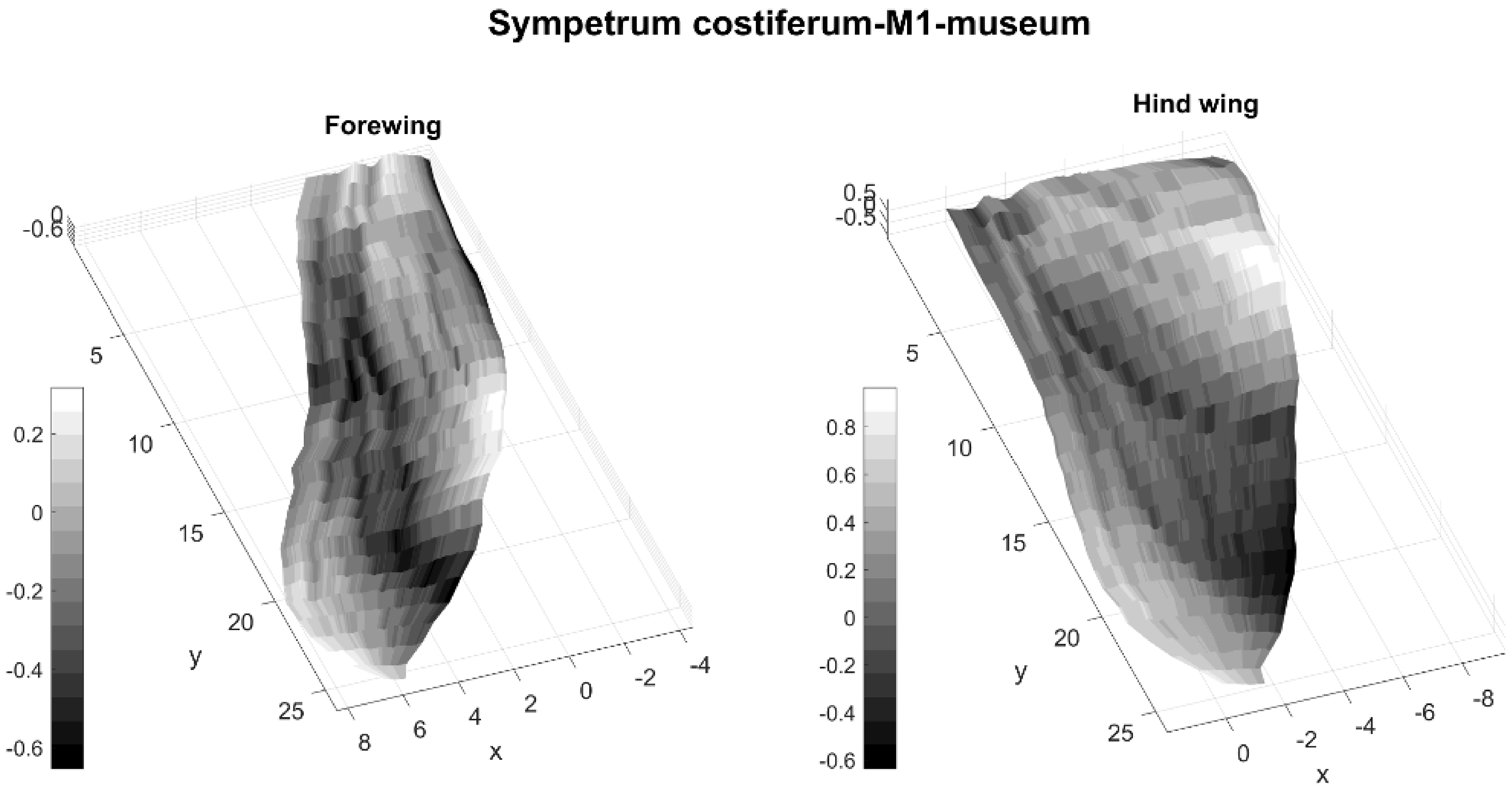
Sympetrum costiferum-M1-museum
Forewing
Hind wing
x
y
0
-0.6
5
10
15
20
25
8
6
4
2
0
-2
-4
0.2
0
-0.2
-0.4
-0.6
0.5
0
-0.5
-6
-8
0.8
0.6
0.4

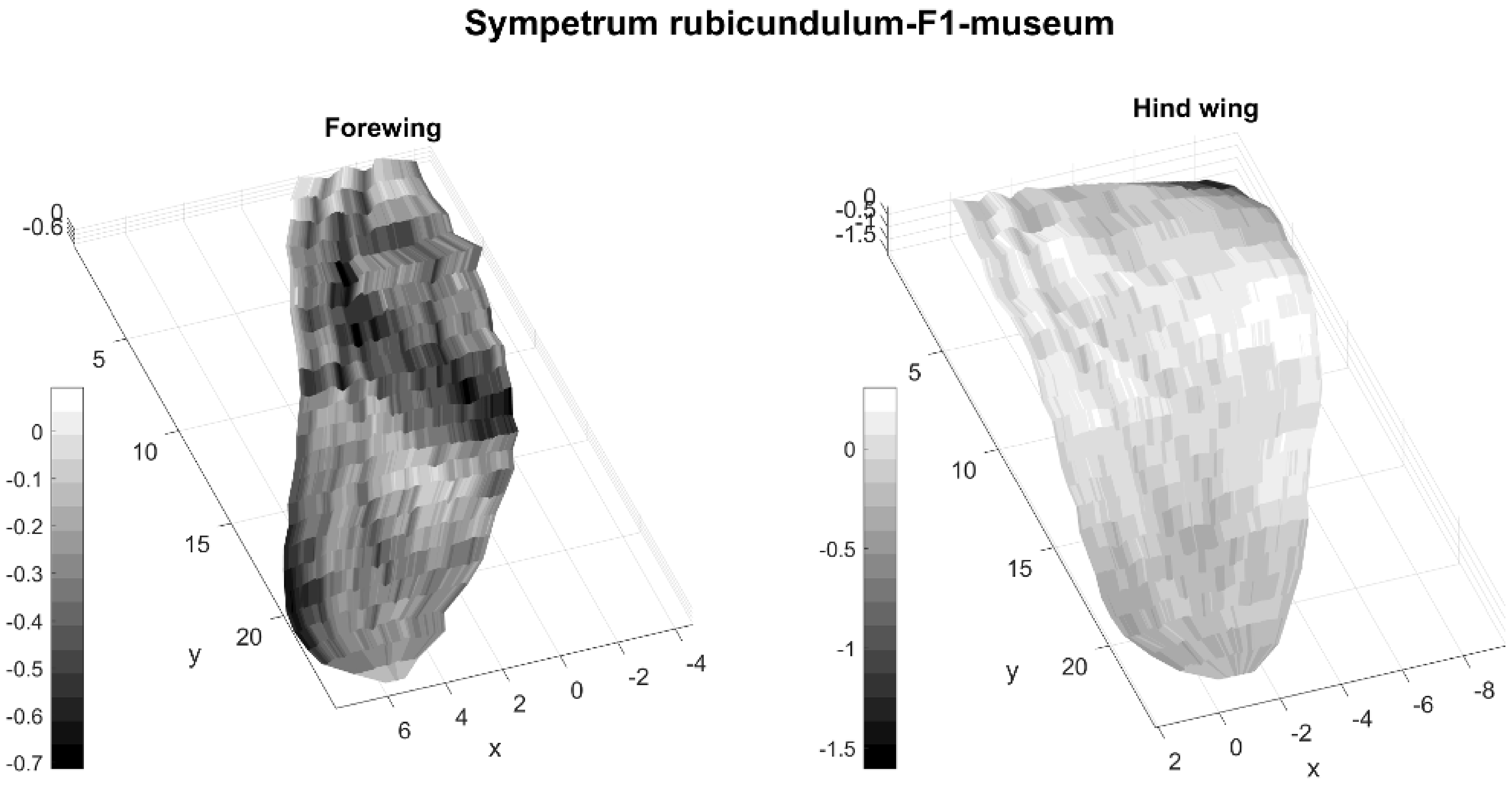
Sympetrum rubicundulum-F1-museum
Forewing
Hind wing
x
y

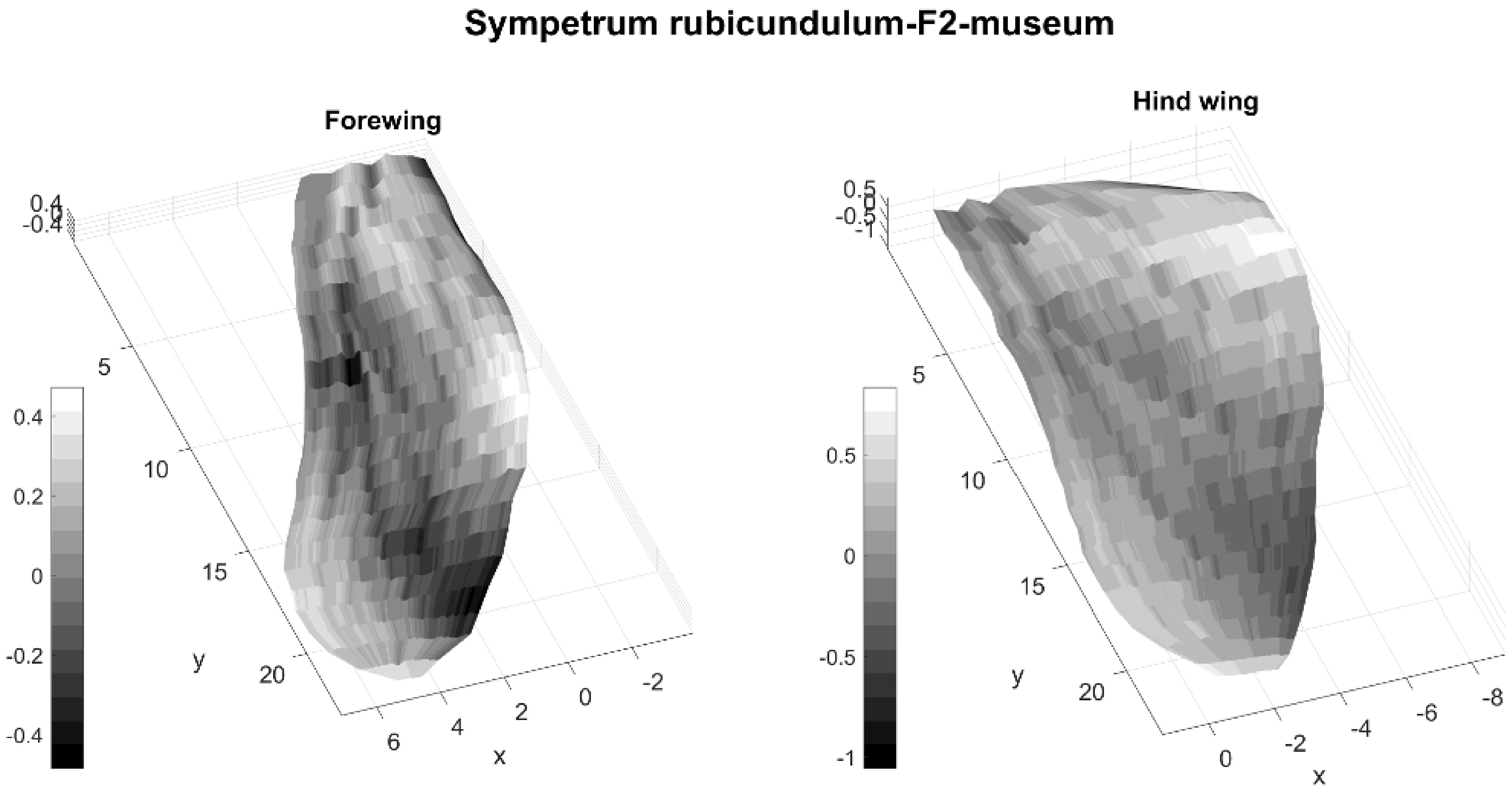

Sympetrum rubicundulum-F2-museum
Forewing
Hind wing
0.4
0
-0.4
5
10
15
20
y
6
4
2
0
-2
x
0.4
0.2
0
-0.2
-0.4
0.5
0
-0.5
-1
5
10
15
20
y
0
-2
-4
-6
-8
x
0.5
0
-0.5
-1

## Sympetrum rubicundulum-F3-museum

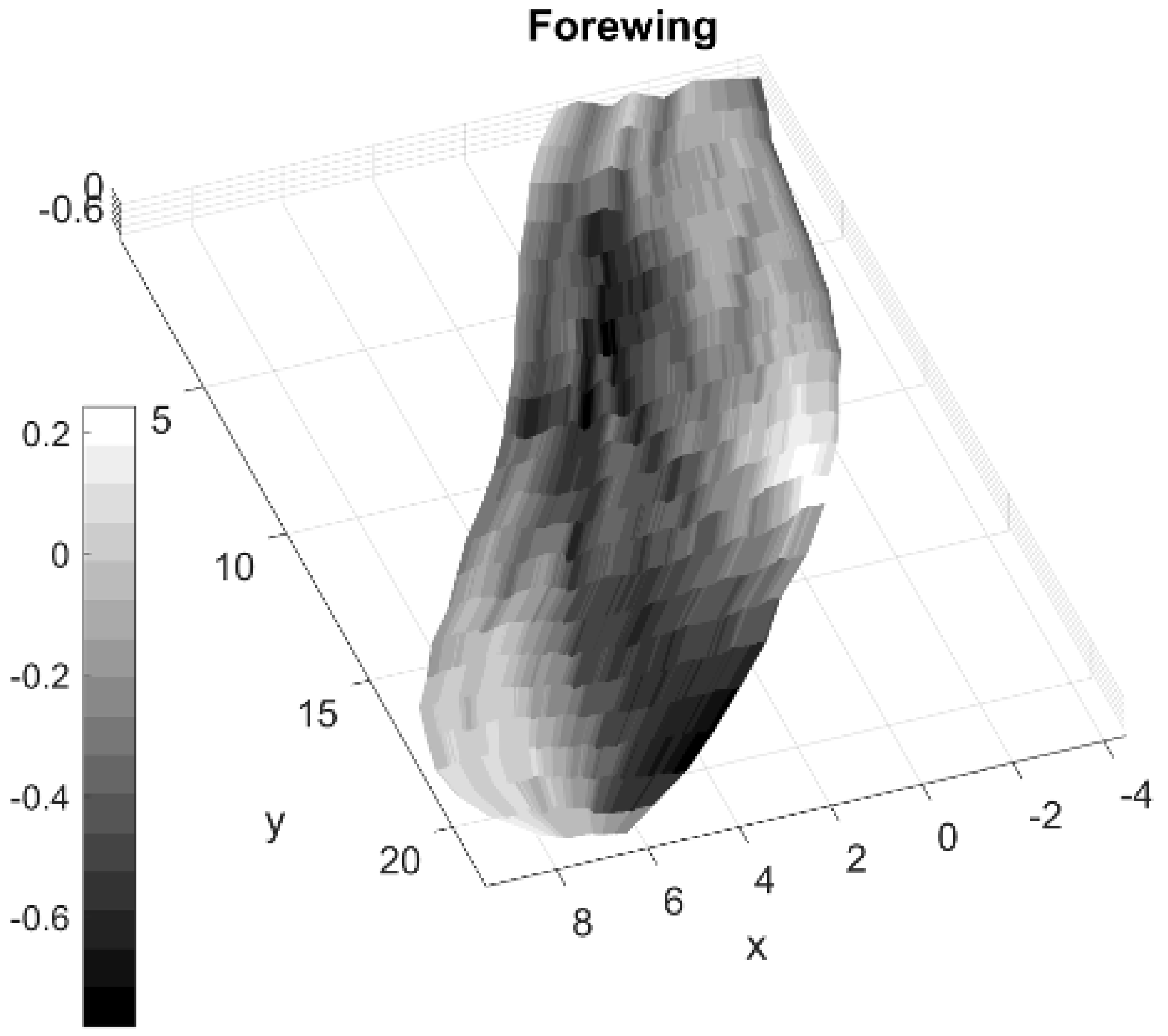


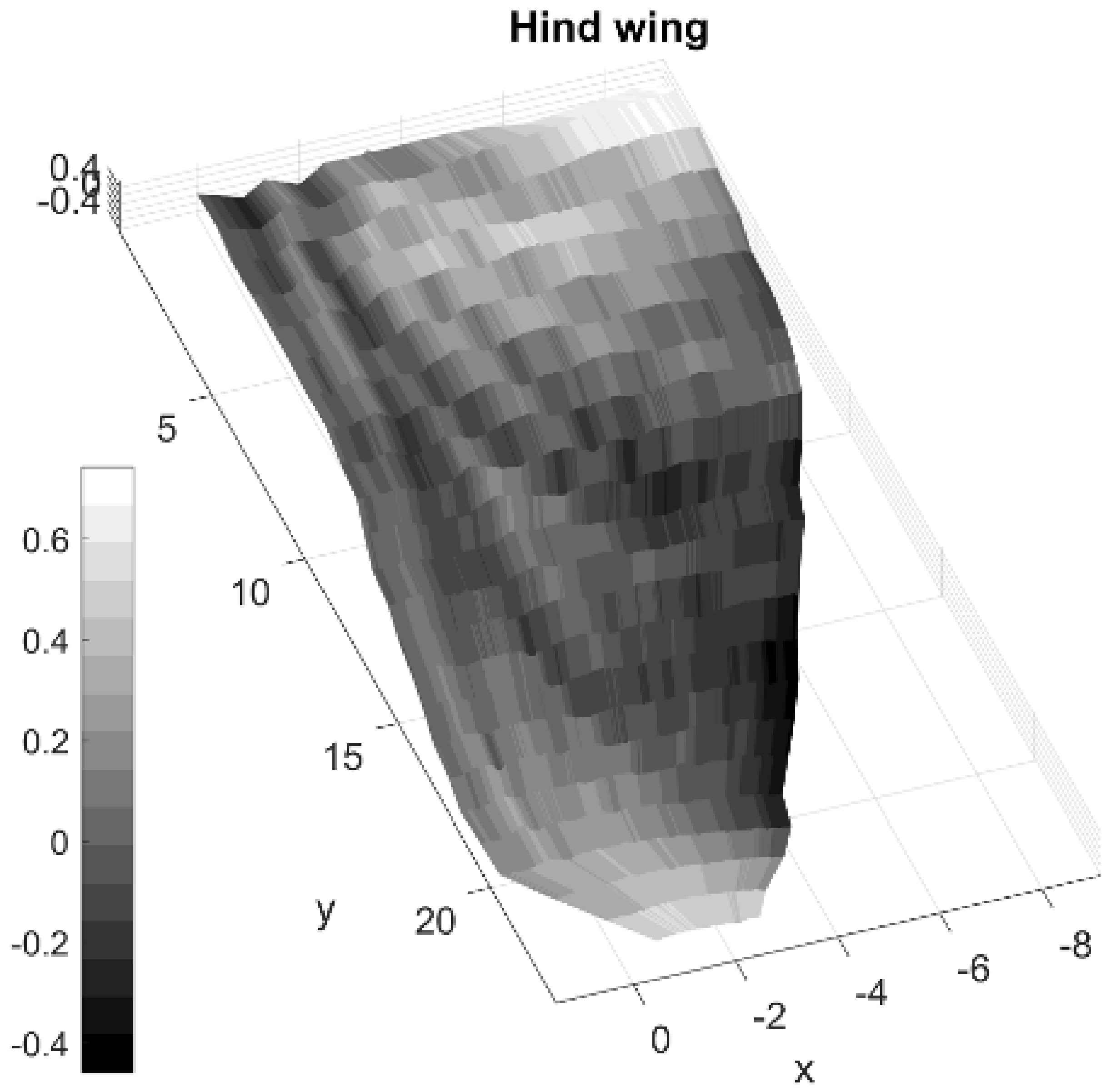

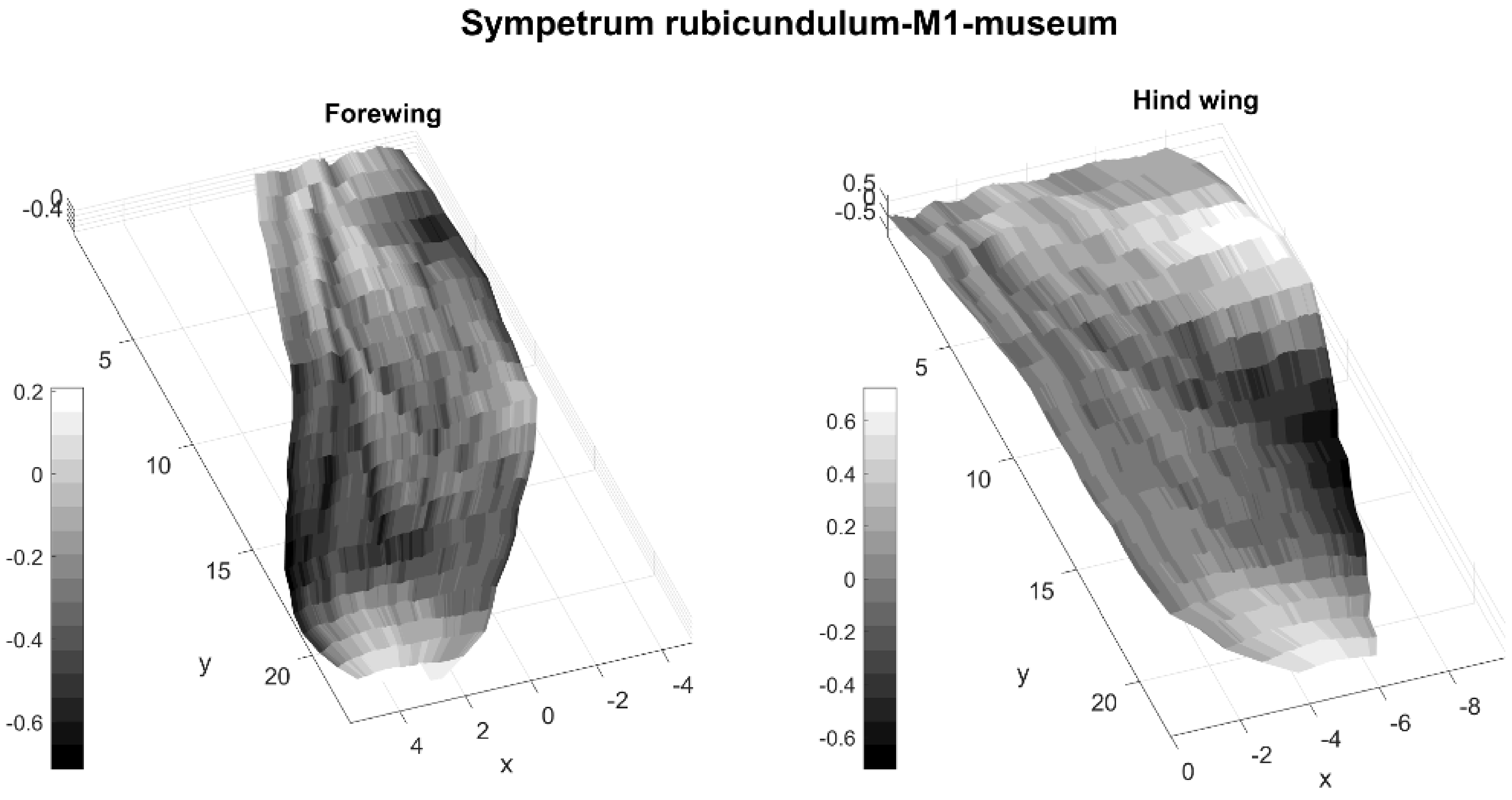
Sympetrum rubicundulum-M1-museum
Forewing
Hind wing
0
-0.4
0.5
0
-0.5
5
10
15
20
y
4
2
0
-2
-4
x
0.2
0
-0.2
-0.4
-0.6
5
10
15
20
y
0
-2
-4
-6
-8
x
0.6
0.4
0.2
0
-0.2
-0.4
-0.6

**Sympetrum rubicundulum-M2-museum**

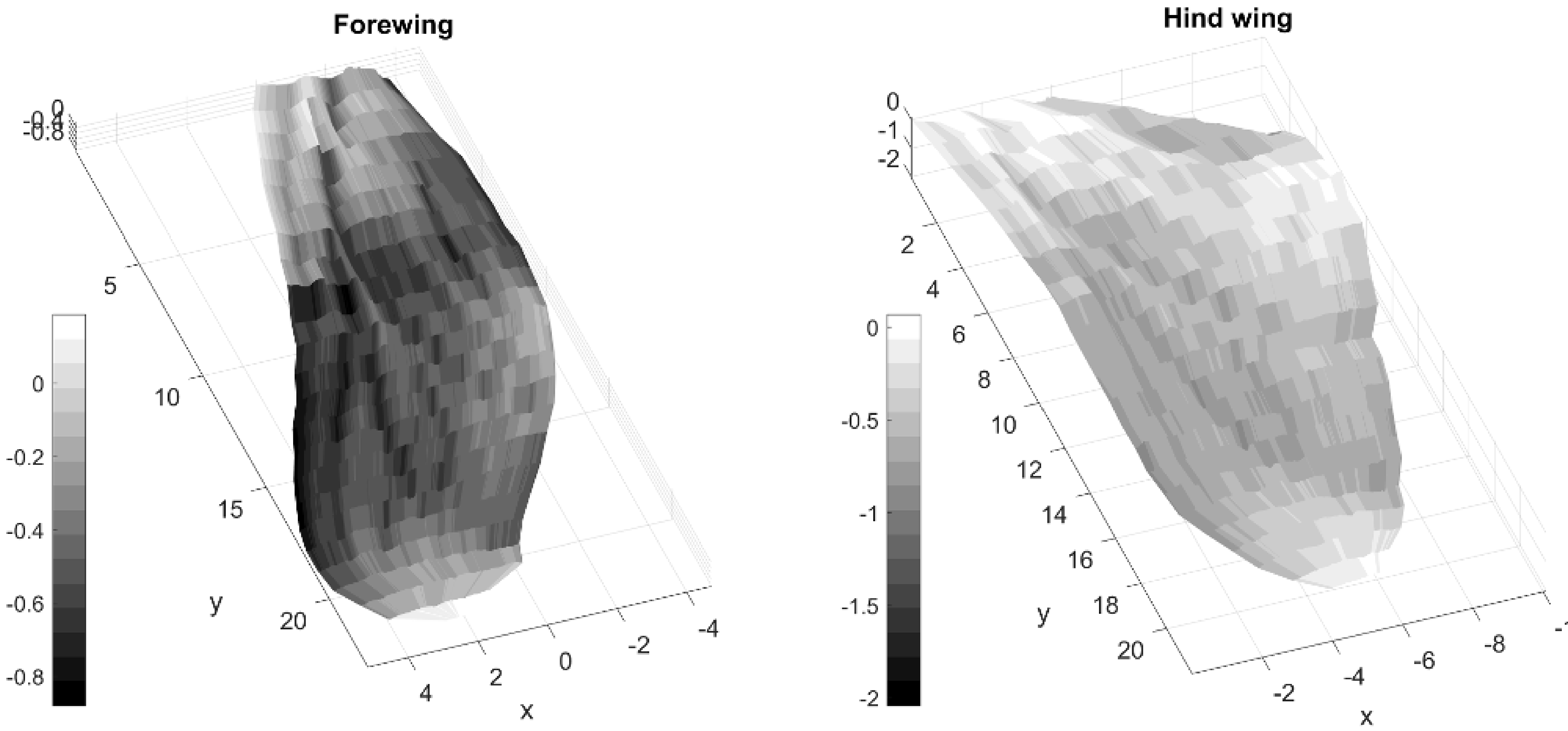

# Sympetrum rubicundulum-M3-museum

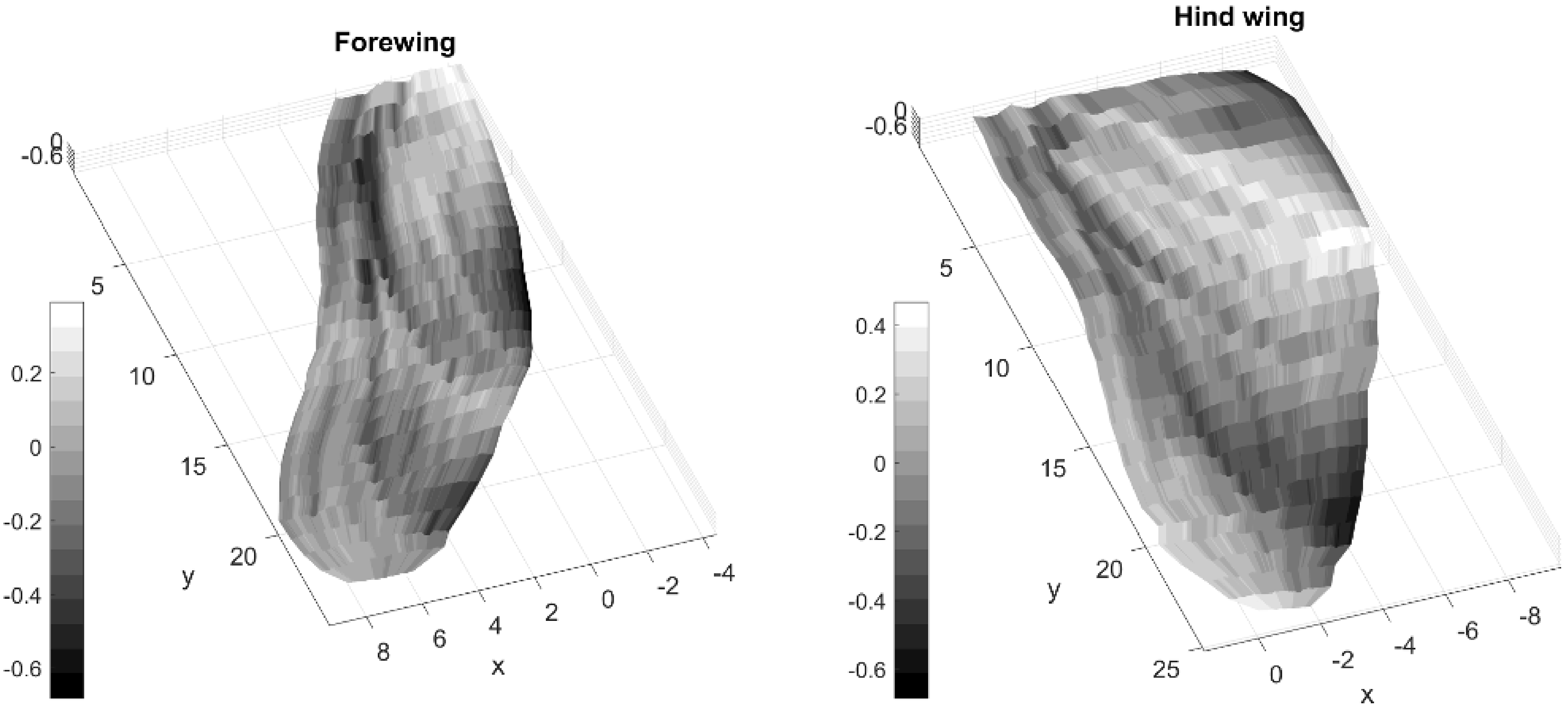

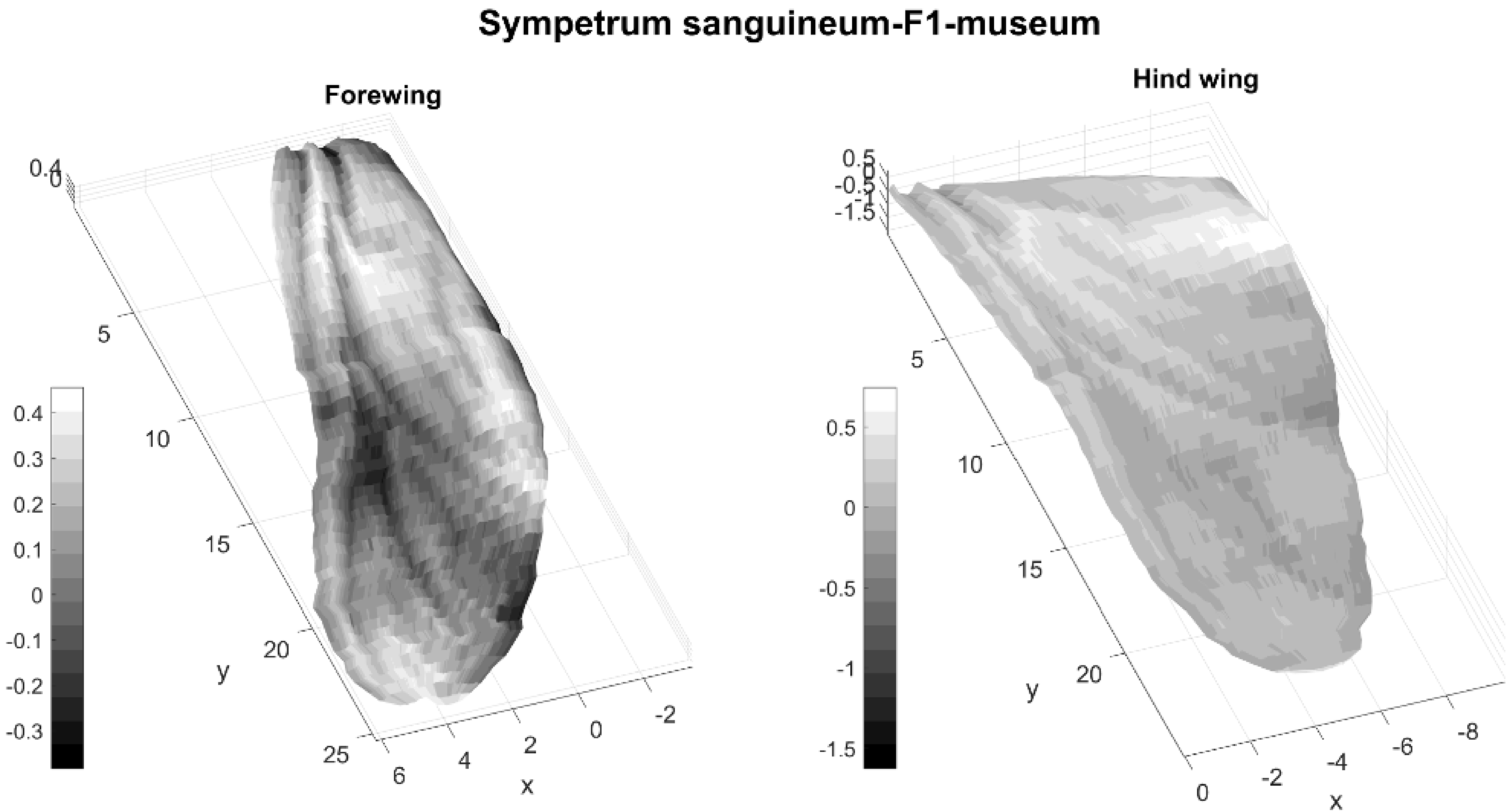

Sympetrum sanguineum-F1-museum
Forewing
Hind wing

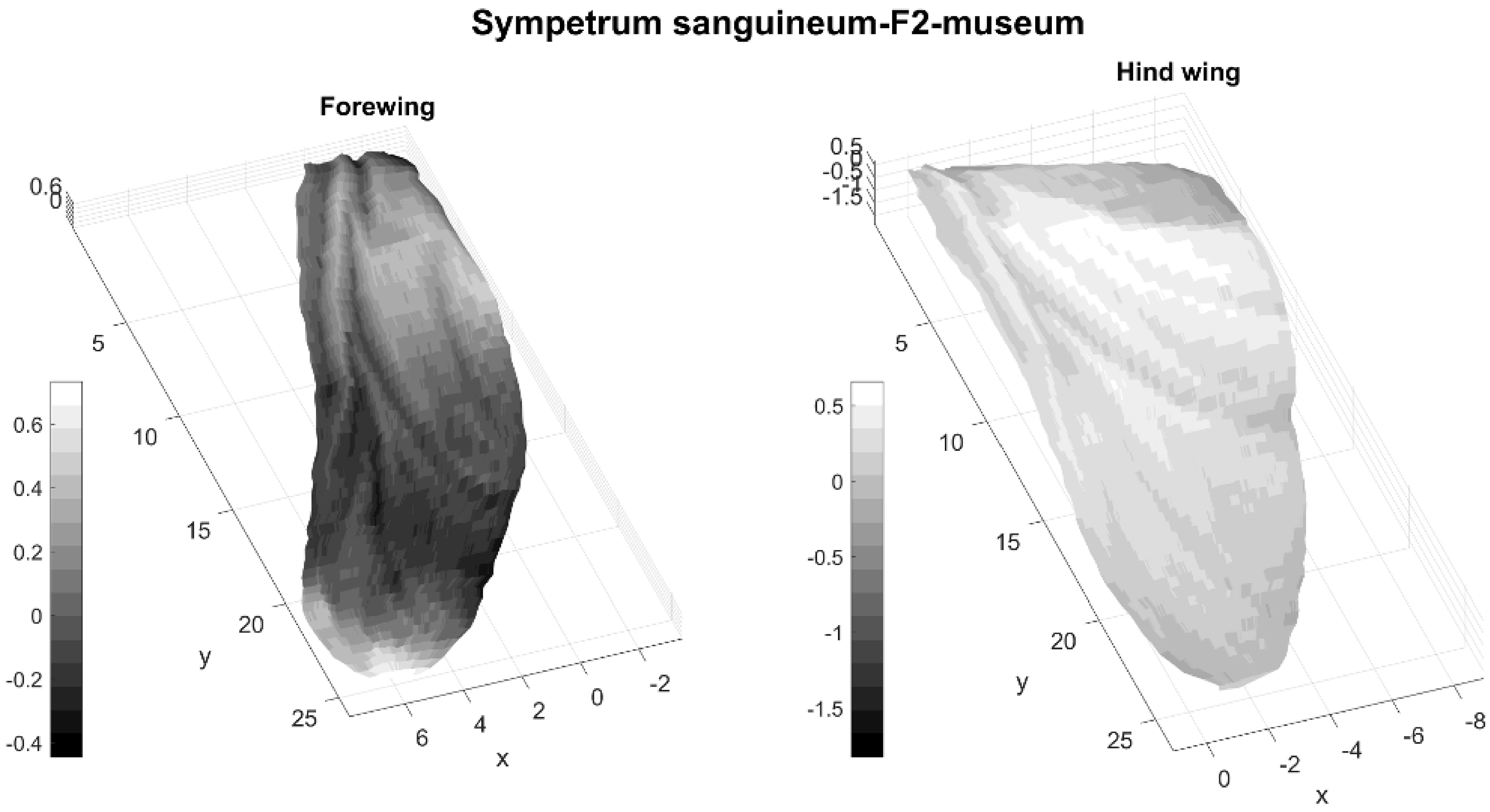
Sympetrum sanguineum-F2-museum
Forewing
Hind wing
0.6
0
5
10
15
20
25
y
6
4
2
0
-2
x
0.6
0.4
0.2
0
-0.2
-0.4
0.5
0
-0.5
-1
-1.5
5
10
15
20
25
y
0
-2
-4
-6
-8
x
0.5
0
-0.5
-1
-1.5

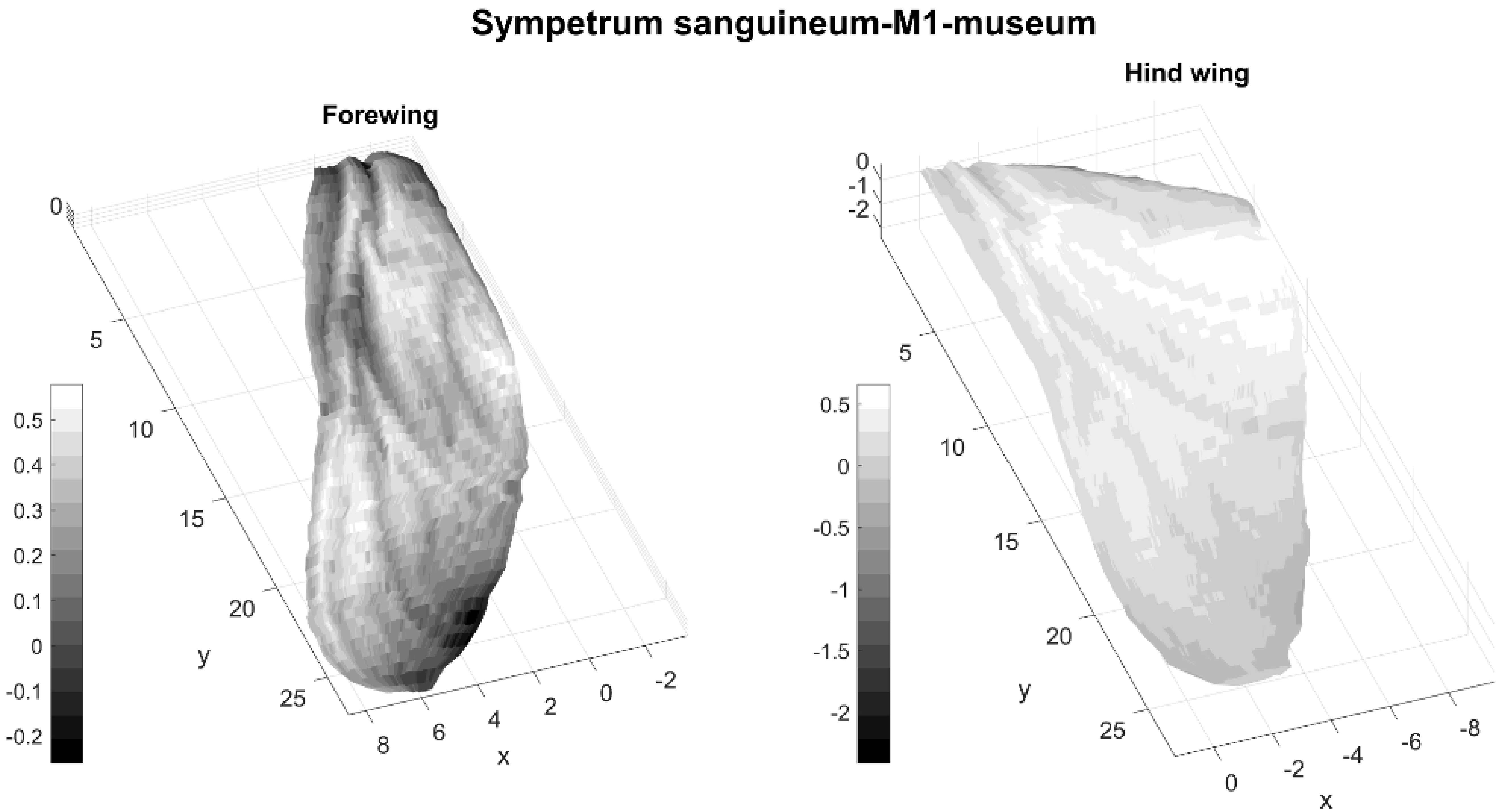
Sympetrum sanguineum-M1-museum
Forewing
Hind wing
x
y

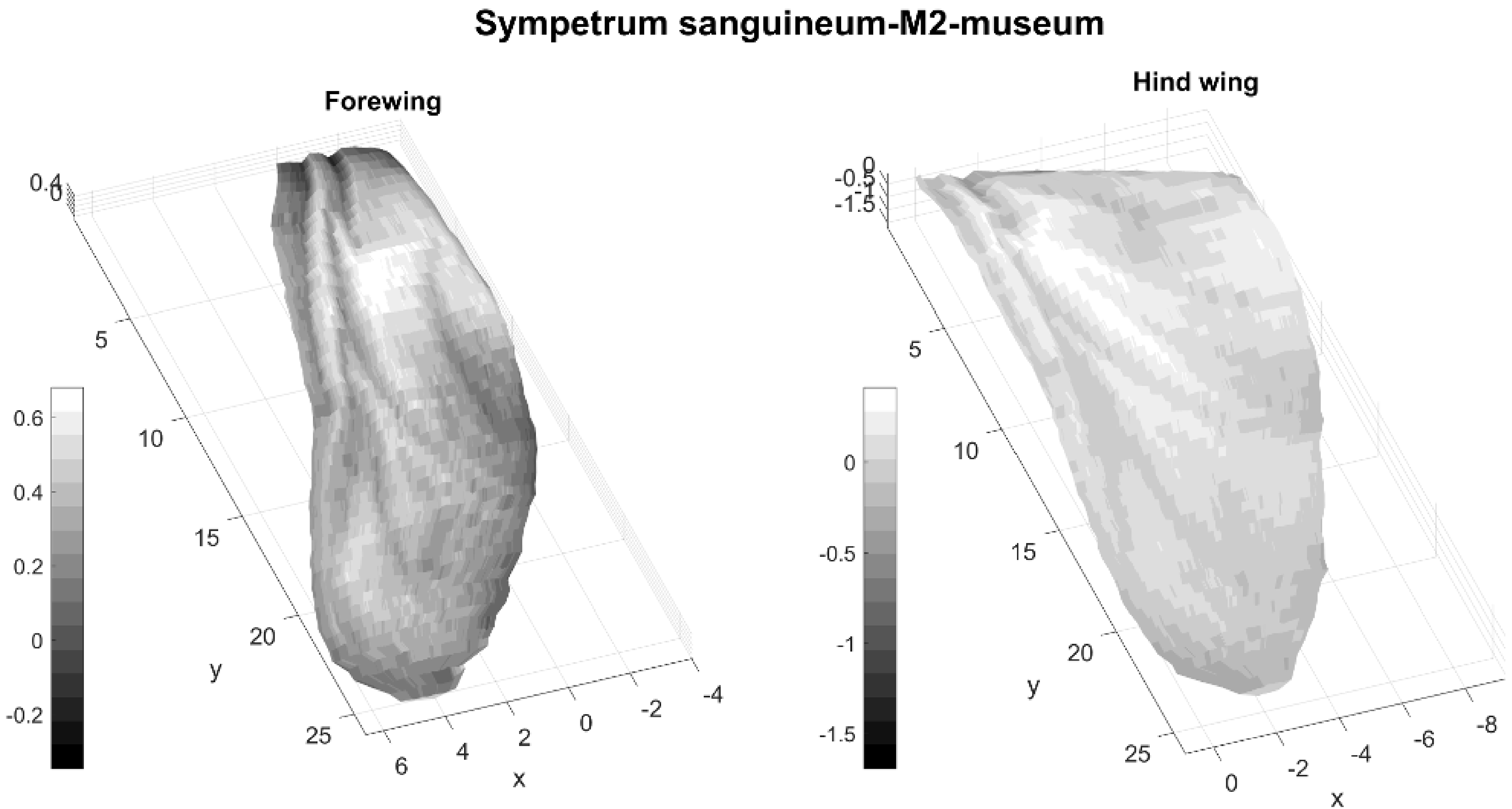

Sympetrum sanguineum-M2-museum
Forewing
Hind wing
x
y

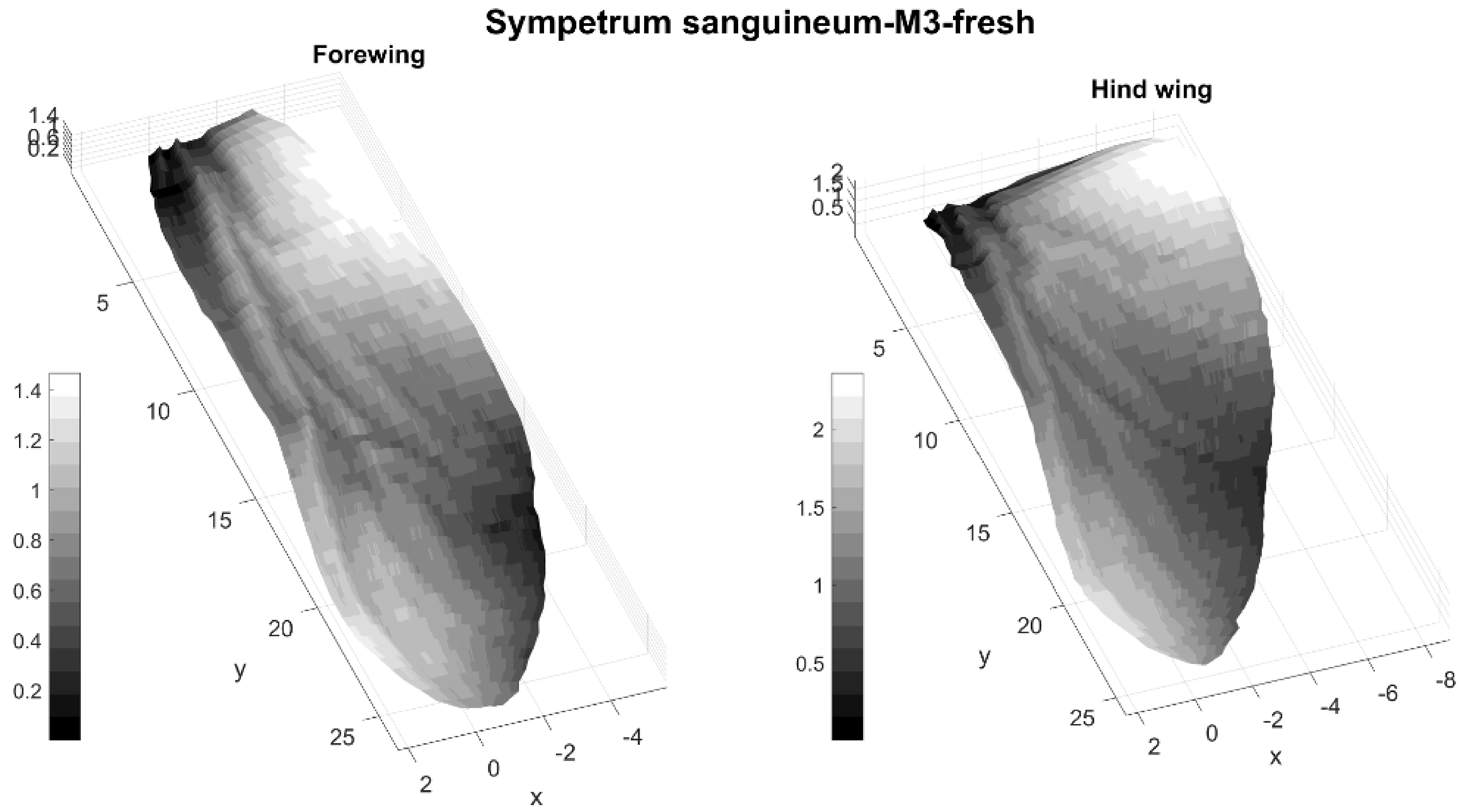

Sympetrum sanguineum-M3-fresh
Forewing
Hind wing
1.4
0.6
0.2
5
10
15
20
25
y
x
2
0
-2
-4
1.4
1.2
1
0.8
0.6
0.4
0.2
2
1.5
0.5
5
10
15
20
25
-6
-8
2
1.5
1
0.5

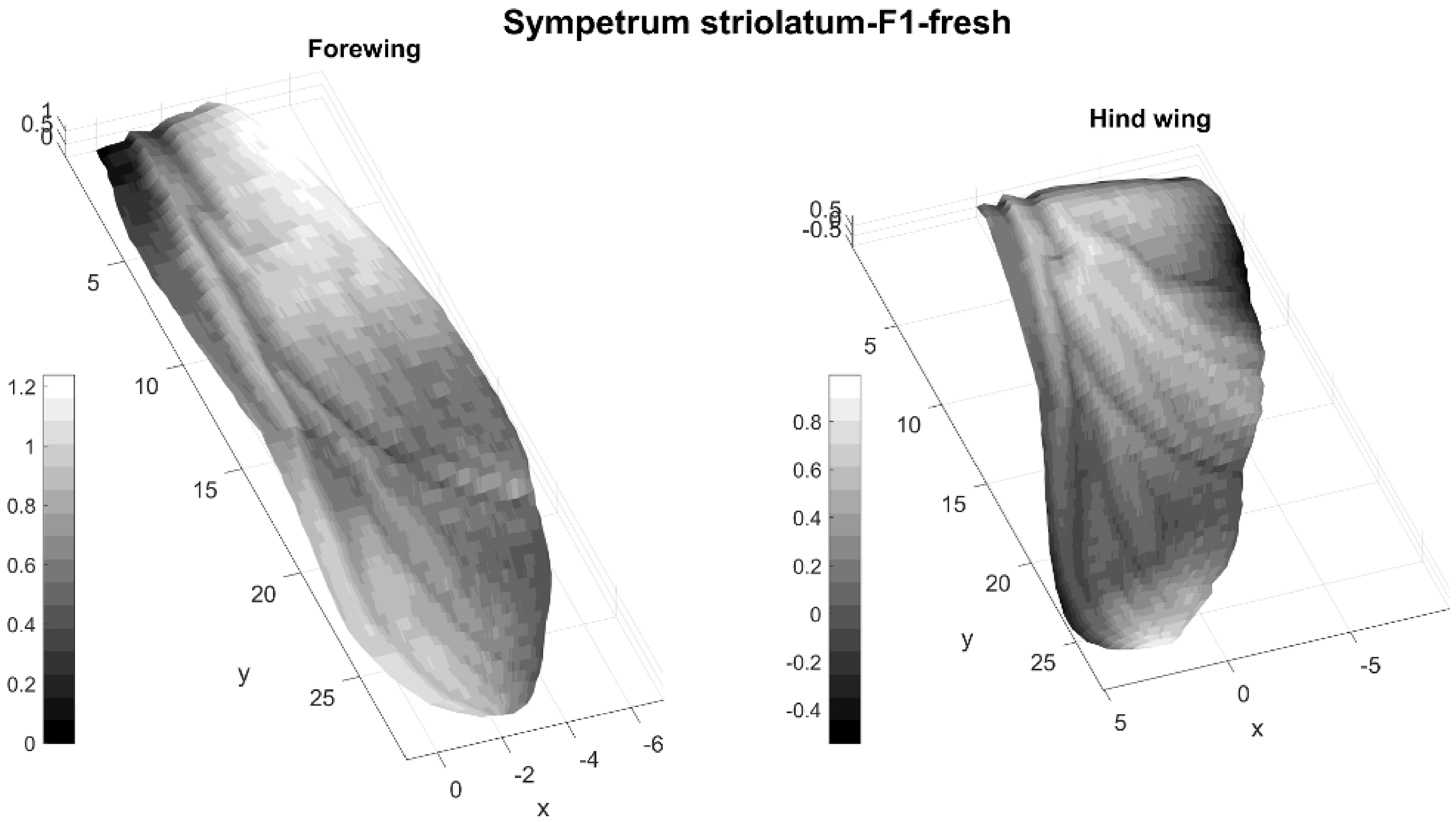

Sympetrum striolatum-F1-fresh
Forewing
Hind wing
x
y

**Tholymis tillarga-F1-museum**

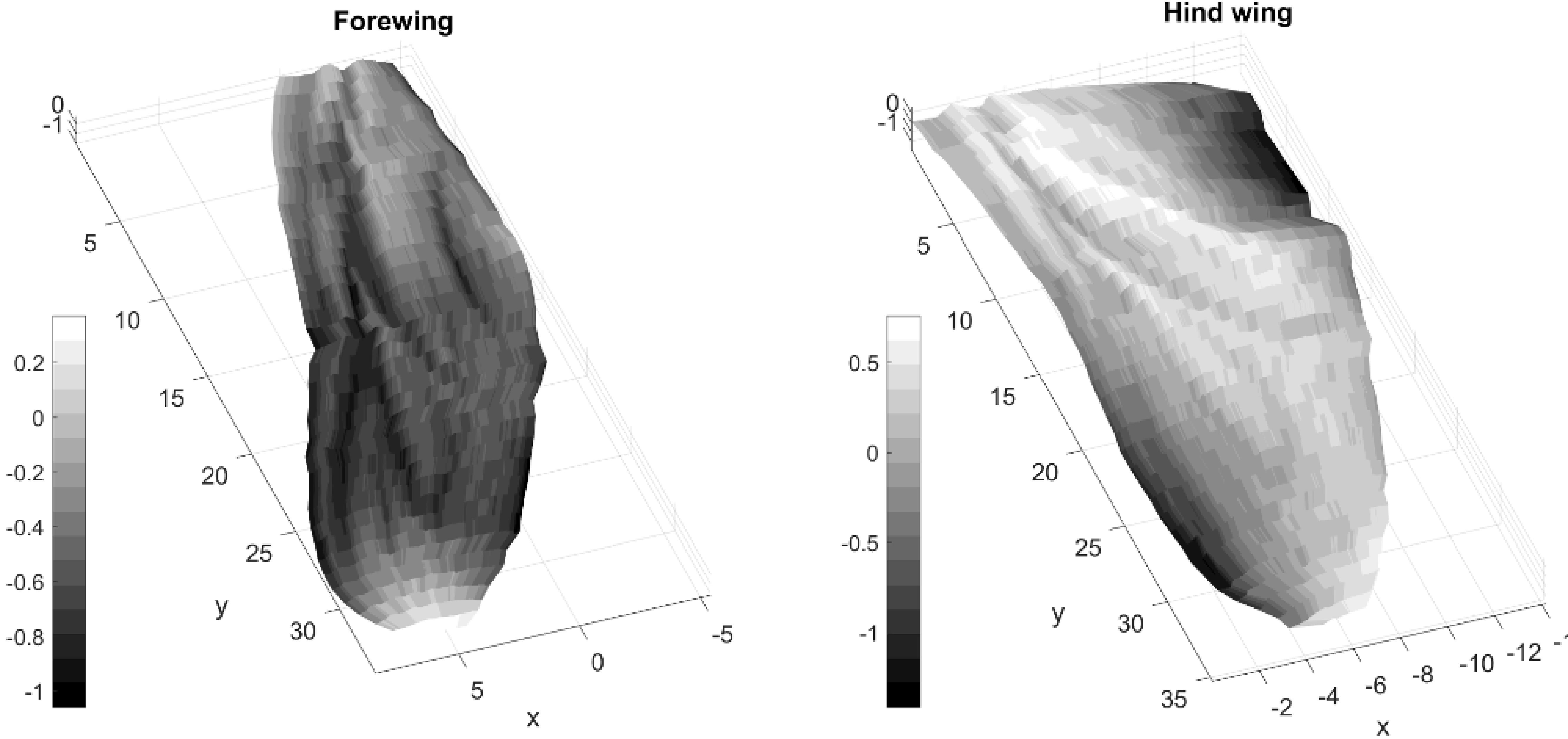

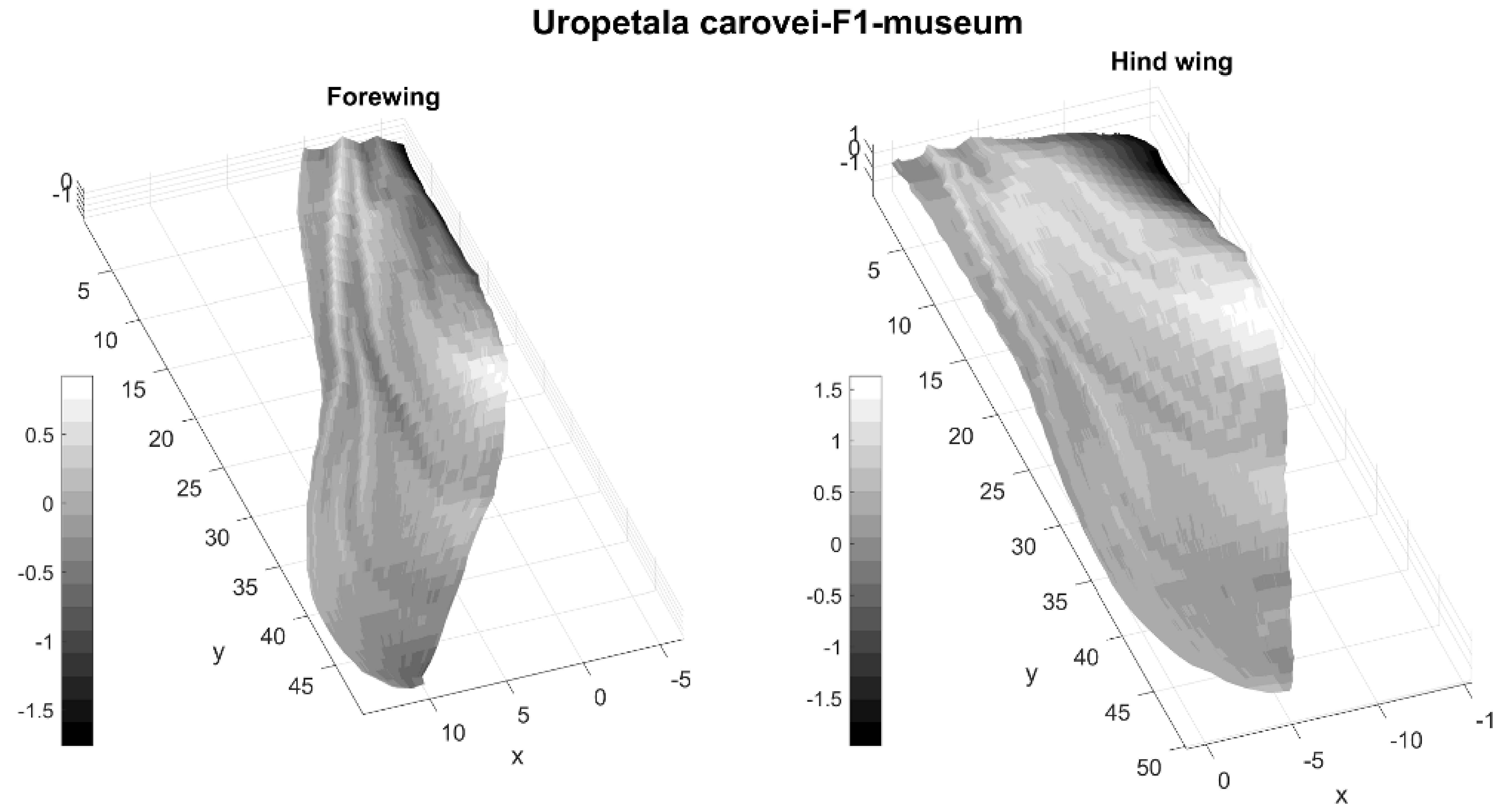
Uropetala carovei-F1-museum
Forewing
Hind wing
x
y
x
y

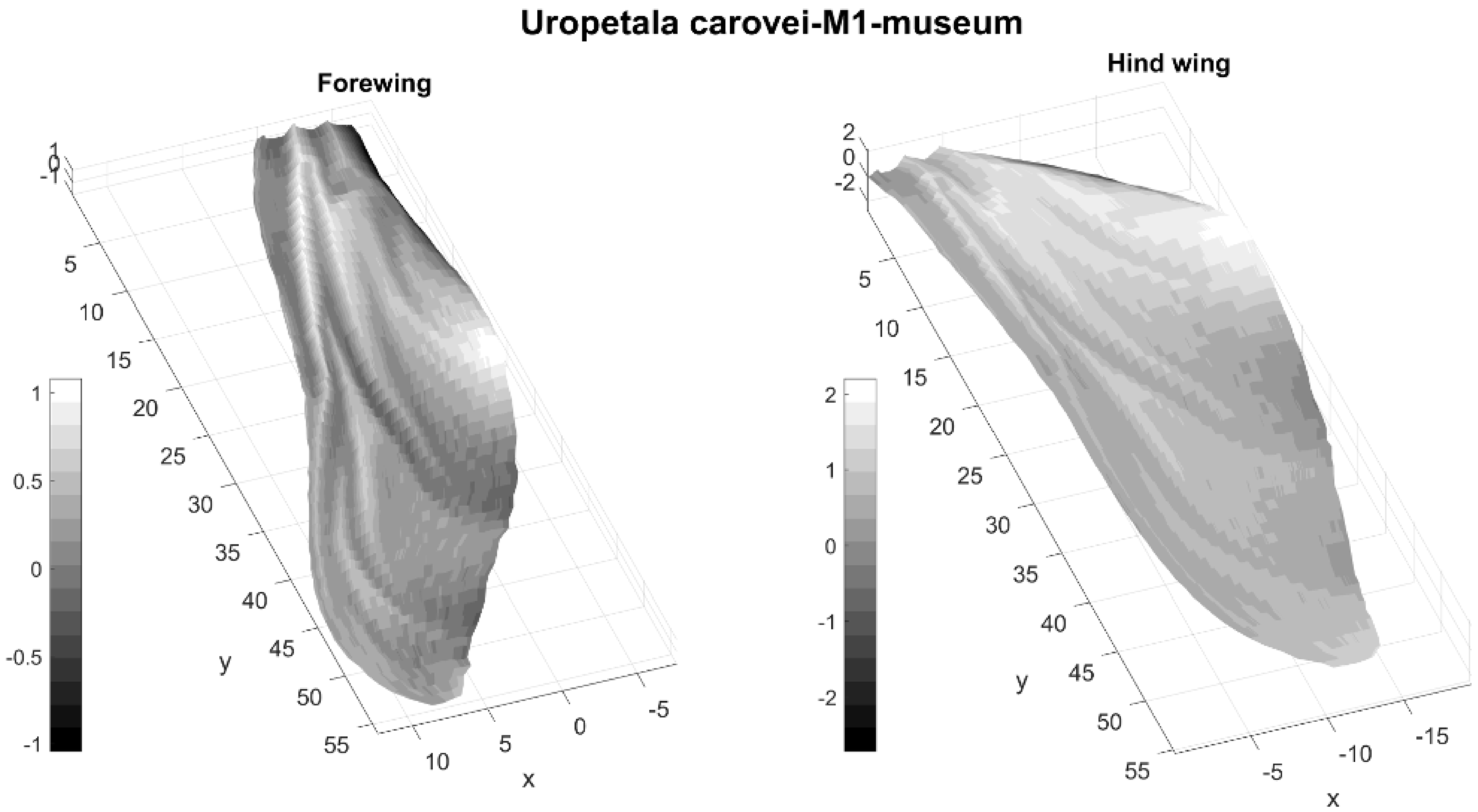

Uropetala carovei-M1-museum
Forewing
Hind wing
y
x
y
x

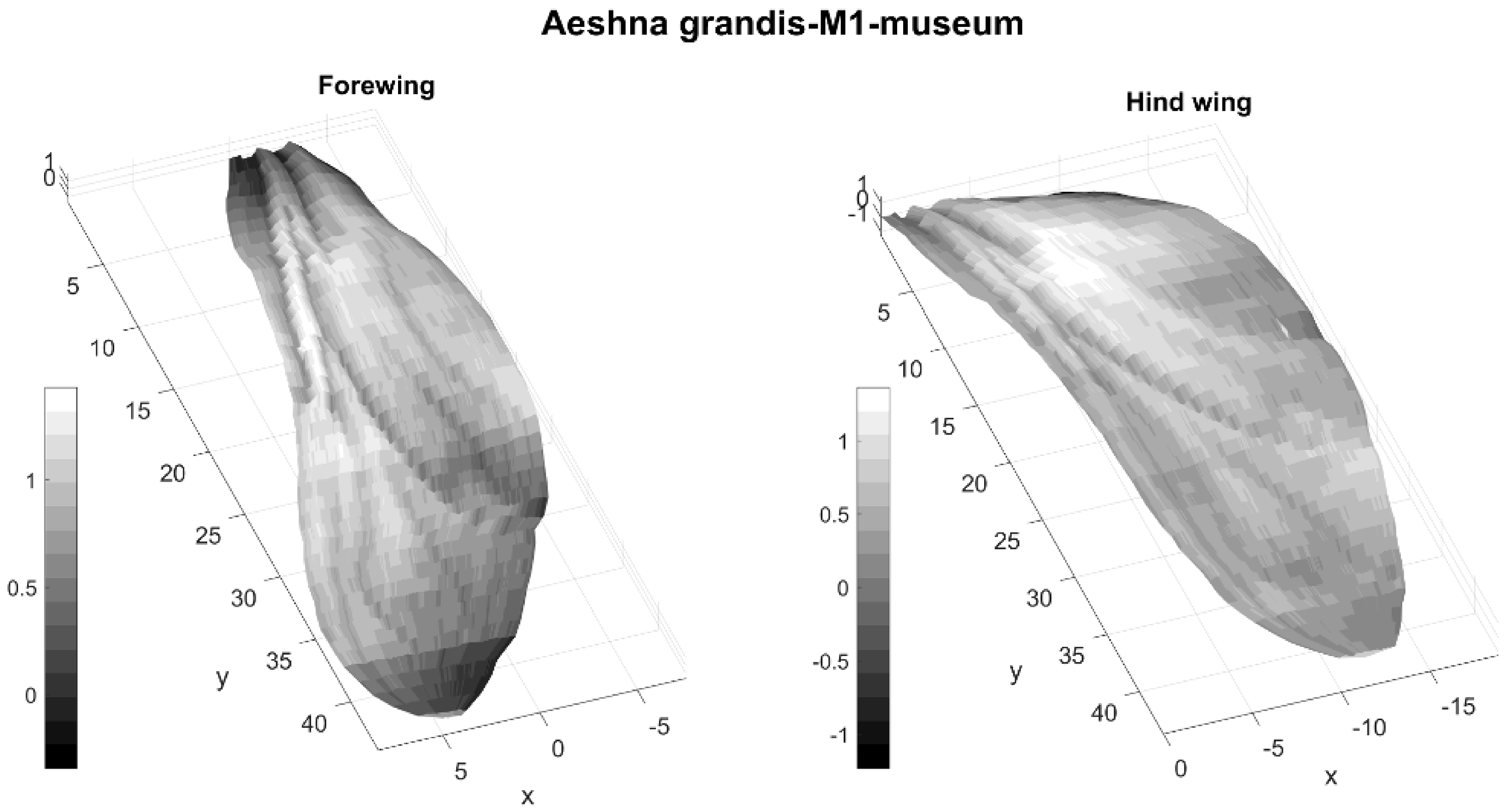
Aeshna grandis-M1-museum
Forewing
Hind wing
y
x
1
0
5
10
15
20
25
30
35
40
-5
0.5
-1
-0.5
-10
-15

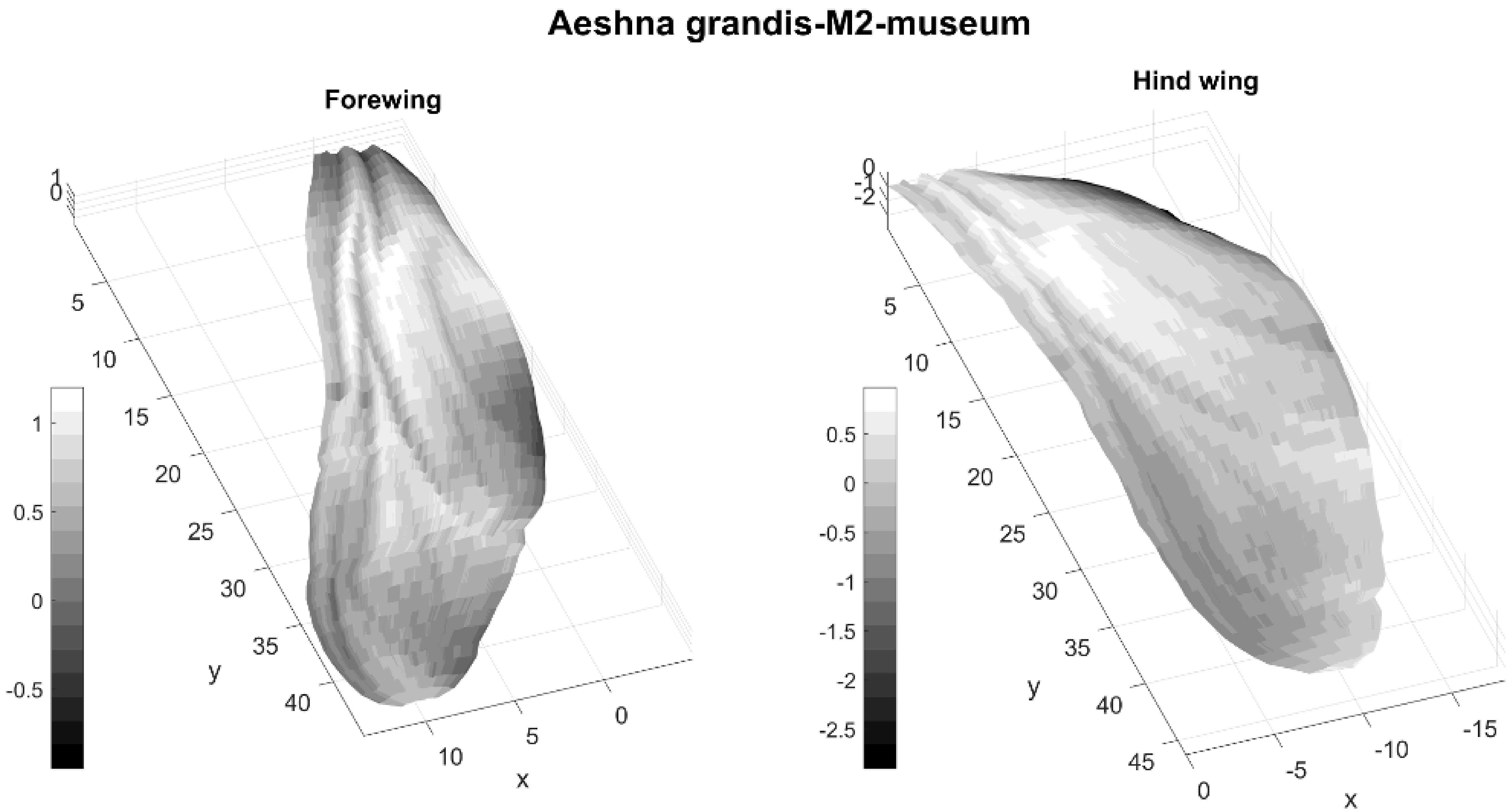
Aeshna grandis-M2-museum
Forewing
Hind wing
x
y
x
y

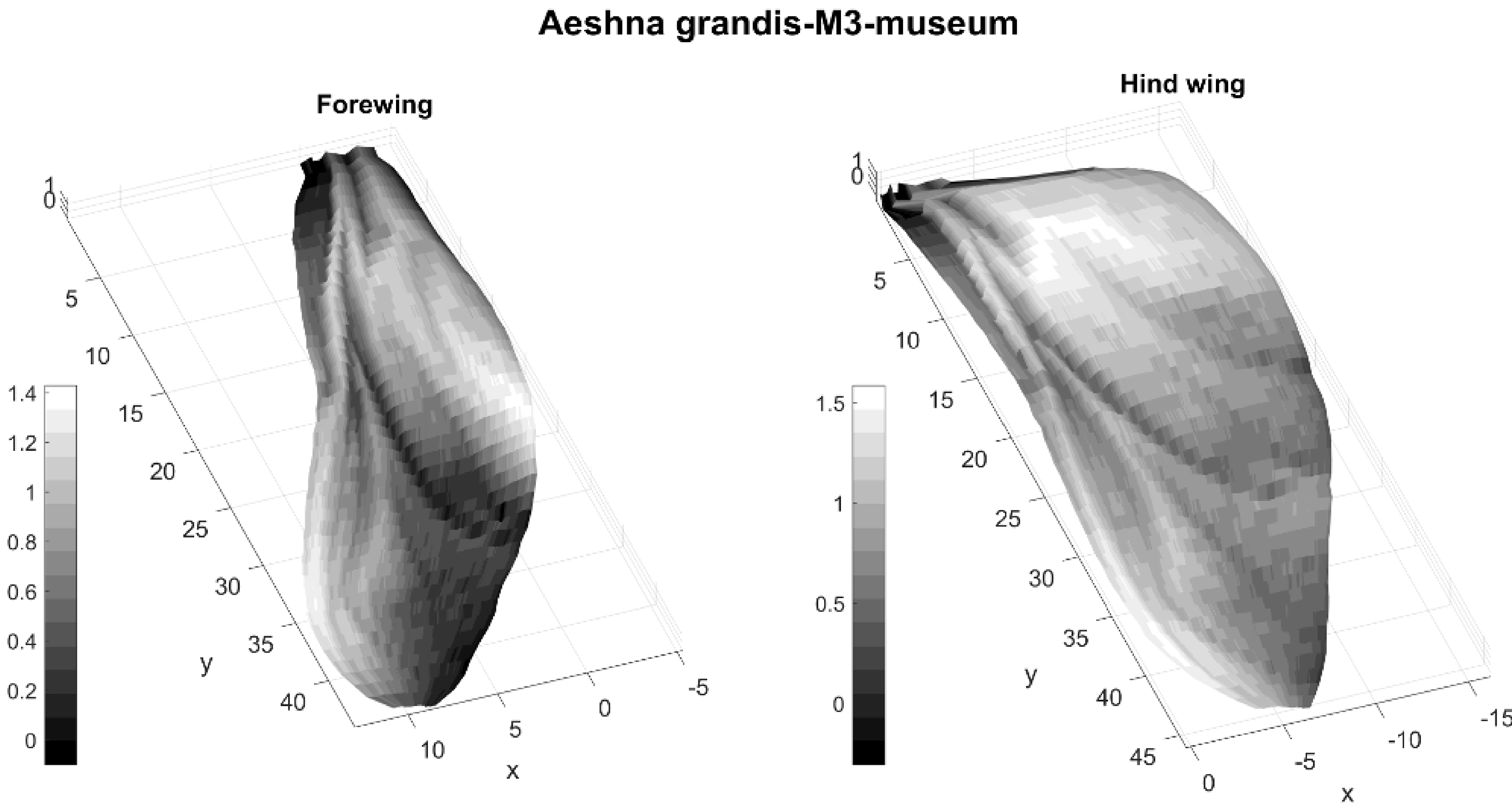

Aeshna grandis-M3-museum
Forewing
Hind wing
x
y

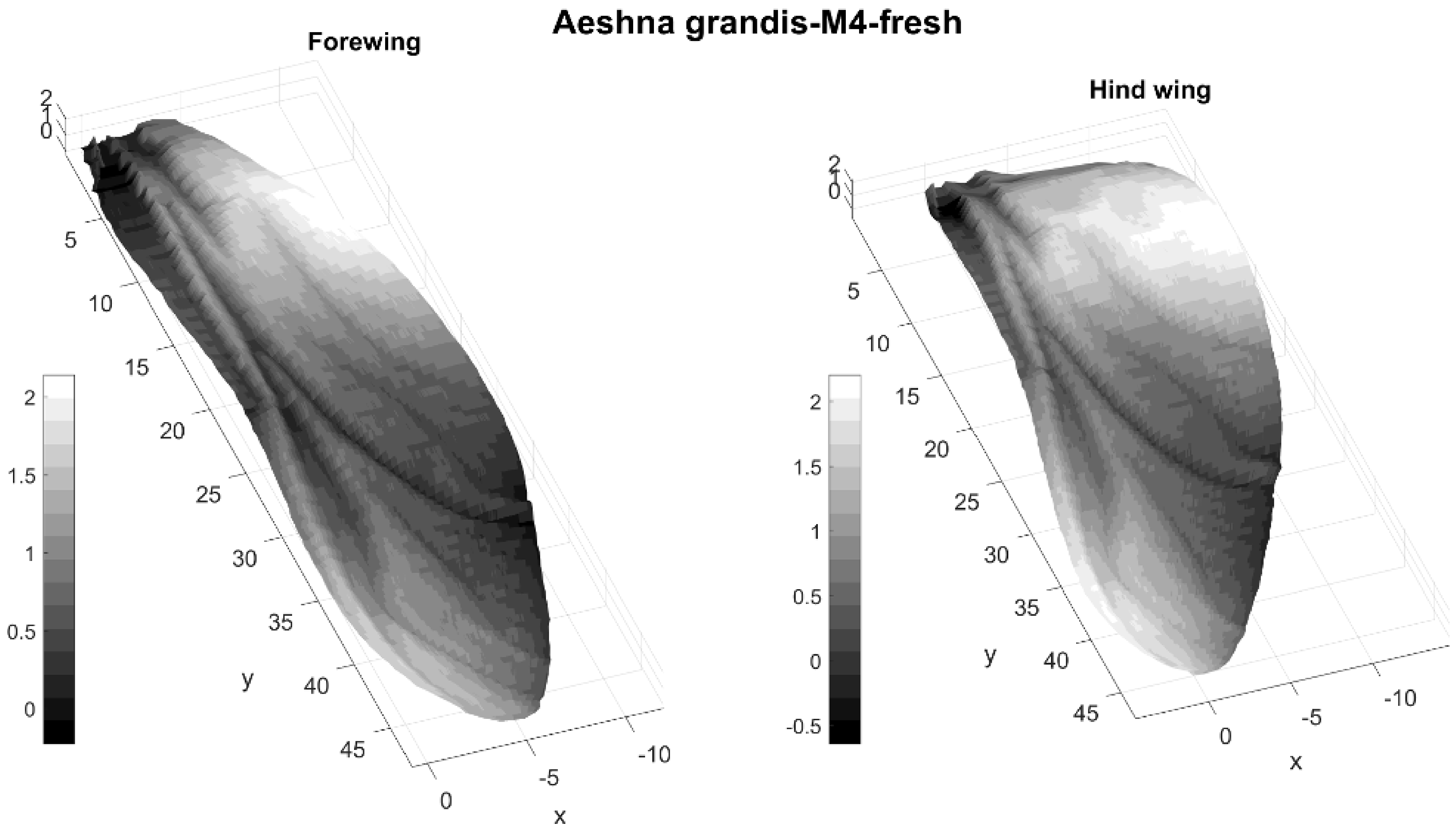
Aeshna grandis-M4-fresh
Forewing
Hind wing
2
1
0
5
10
15
20
25
30
35
40
45
y
0
-5
-10
x
2
1.5
1
0.5
0
-0.5

# Aeshna grandis-M5-fresh

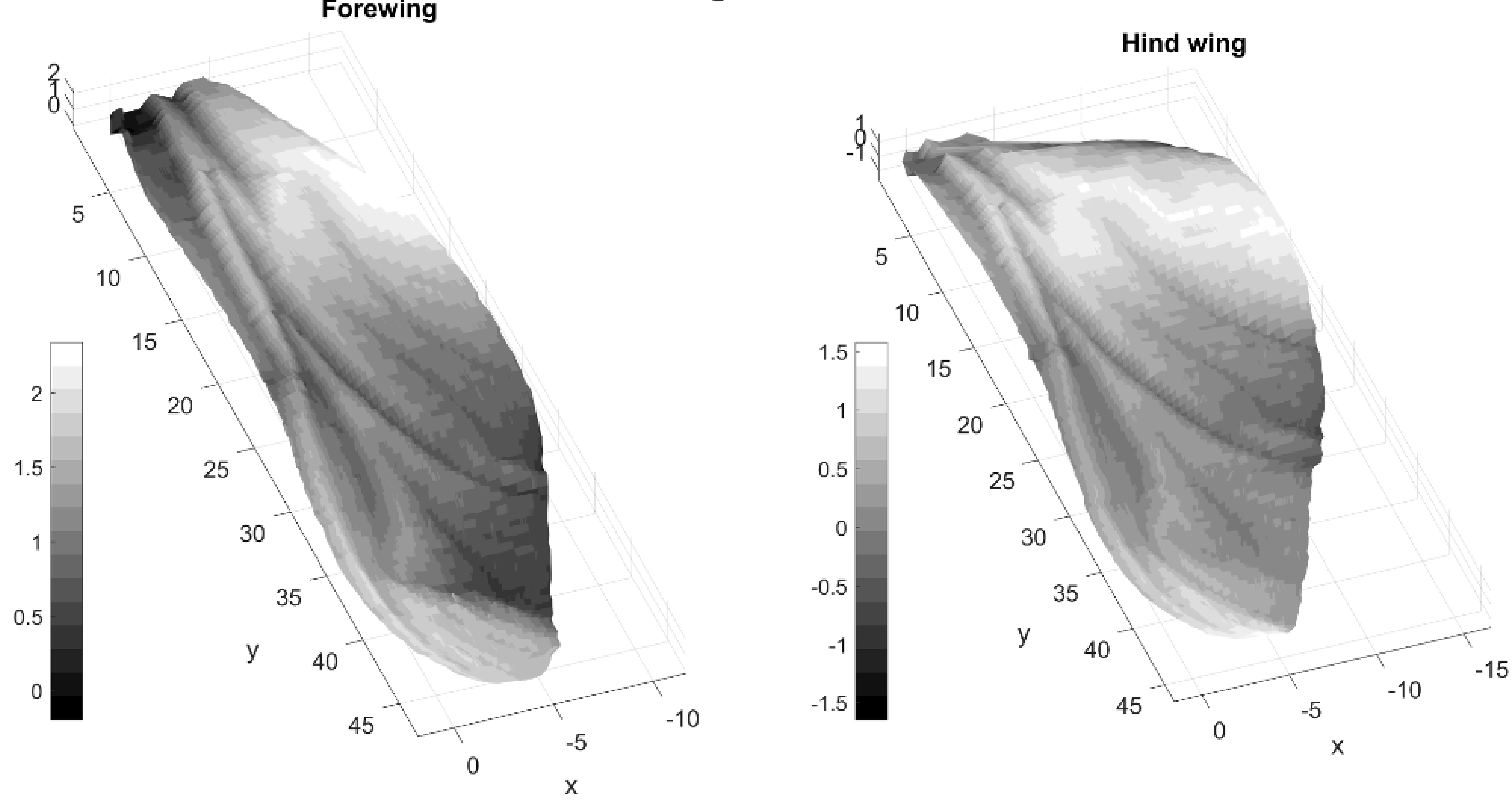

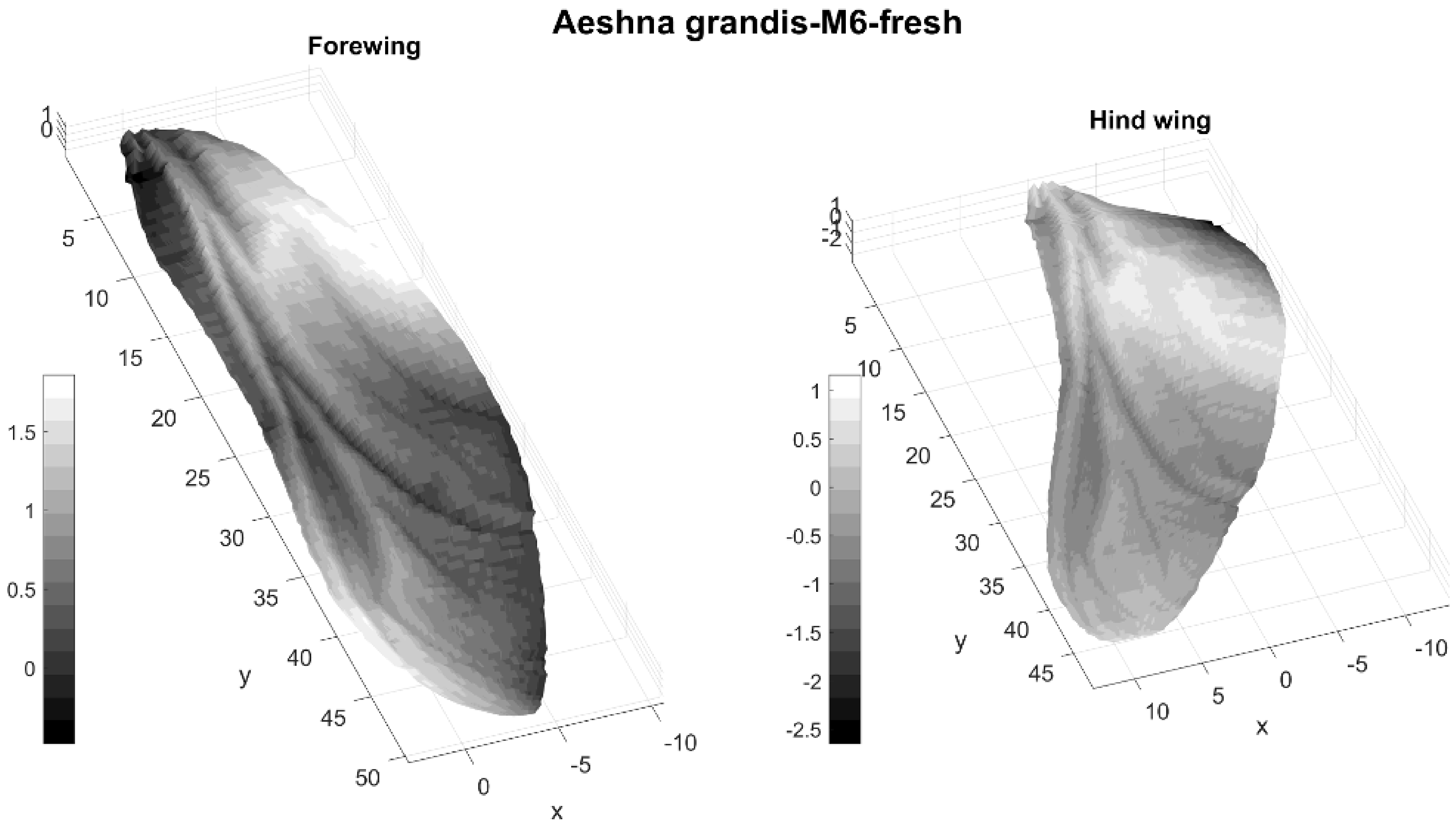
Aeshna grandis-M6-fresh
Forewing
Hind wing
x
y
