# Supplementary material for: Flight of the dragonflies and damselflies
Source: Philos Trans R Soc Lond B Biol Sci. 2016 Sep 26;371(1704):20150389. doi: 10.1098/rstb.2015.0389 (PMC4992713; doi:10.1098/rstb.2015.0389)
Supplement: Supplementary Data 3: Grid convergence data. [file rstb20150389supp4.pdf]

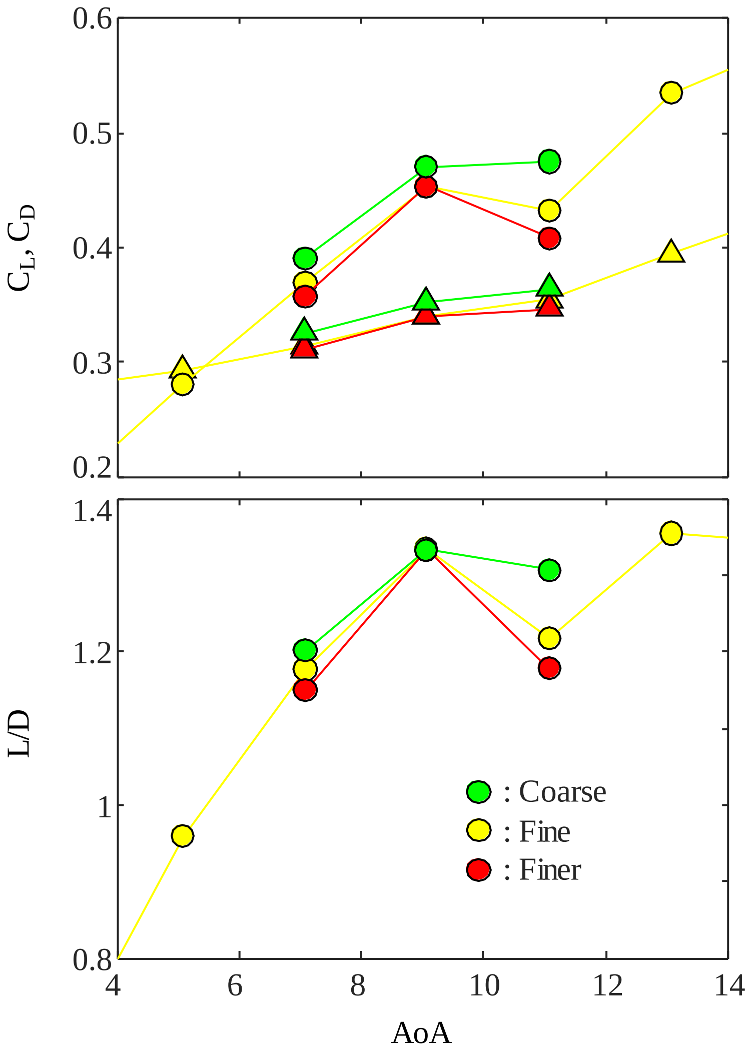

	Number of grid points (global grid) (wing grid)	Mean CL (mean error)	Mean CD (mean error)	Mean L/D (mean error)
Coarse	241 x 251 x 17<br>65 x 41 x 73	0.35<br>(3.3 %)	0.45<br>(6.5 %)	1.28<br>(3.1 %)
Fine	301 x 321 x 21<br>81x51x91	0.34<br>(-)	0.42<br>(-)	1.24<br>(-)
Finer	361 x 385 x 25<br>97 x 61 x 109	0.33<br>(-1.1 %)	0.41<br>(-3.0 %)	1.22<br>(-1.8 %)

Supplementary Data 3. Simulated lift and drag coefficient (C_L and C_D), and lift to drag ratio (L/D) for various levels of grid refinement with the exaggerated corrugation model (X4 in figure 2). Three different angles of attack were tested. Mean C_L, C_D, L/D, and error of the three cases are summarized in table. While the coarse mesh gives slightly higher value especially at higher angle of attack, the fine and finer meshes show small differences in the simulated forces.
